# Photocatalytic Asymmetric C‐C Coupling for CO_2_ Reduction on Cu‐Zn Bimetallic Catalysts with Dipole‐Limiting Effects

**DOI:** 10.1002/advs.202521670

**Published:** 2025-12-08

**Authors:** Peidong Ma, Ying Yu, Fan Wen, Hongyan Guo, Boyu Li, GuoZhi Deng, Xianyang Shi, Daochuan Jiang, Ruting Huang

**Affiliations:** ^1^ Anhui Province Key Laboratory of Wetland Ecosystem Protection and Restoration School of Resources and Environmental Engineering Anhui University Hefei 230601 China; ^2^ Soil and Fertilizer Institute Anhui Academy of Agricultural Sciences Anhui Provincial Key Laboratory of Nutrient Cycling Resources & Environment Hefei Anhui 230031 China; ^3^ School of Materials Science and Engineering Anhui University Hefei 230601 China

**Keywords:** CO_2_ reduction, C‐C coupling, Cu‐Zn bimetallic catalyst, dipole limiting domain field (DLDF), photocatalysis

## Abstract

The photocatalytic reduction of CO_2_ to multicarbon products such as ethylene remains a challenge due to the difficulty in achieving efficient C‐C coupling. This study proposes a novel Cu‐Zn bimetallic catalyst, CuZn‐E‐d‐MOF, synthesized by grafting ethylenediaminetetraacetic acid (EDTA) onto a defect‐rich Ti‐MOF framework (d‐Ti‐BPDC). The catalyst exhibits excellent photocatalytic CO_2_ reduction performance, with an ethylene yield of 308.72 µmol g^−1^ h^−1^ and an electron selectivity of 98.4%. The Cu‐Zn dual sites enhance the adsorption and activation of CO_2_ intermediates through asymmetric charge distribution and the formation of a dipole‐limited domain field (DLDF), facilitating multi‐electron transfer and C‐C coupling. Density functional theory (DFT) calculations show that Cu^2+^/Cu^+^ pairs stabilize *CO intermediates and promote C─C bond formation, while Zn enhances orbital hybridization, improving electron transfer between Cu‐Zn sites and *CO‐*CO intermediates. In situ FTIR analysis confirms the formation of the *CO‐*CO coupling intermediate, elucidating the high selectivity for C_2_ products. This work highlights the role of asymmetric bimetallic sites and localized electric fields in CO_2_ photoreduction, providing a promising strategy for efficient multicarbon product synthesis.

## Graphical abstract

Asymmetric Cu–Zn dual sites on EDTA‐grafted Ti‐MOF amplify the dipole‐limiting domain field (DLDF), facilitating C–C coupling. This strategy yields an exceptional C_2_H_4_ production rate of 308.72 µmol g^−1^ h^−1^ with 98.4% electron selectivity in photocatalytic CO_2_ reduction, highlighting the potential of asymmetric site engineering and local field modulation for multicarbon product synthesis.

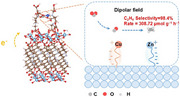

## 1 Introduction

Using solar energy and carbon dioxide as a carbon source for the production of valuable chemicals offers a promising strategy to reduce fossil fuel consumption and mitigate CO_2_ greenhouse gas emissions.^[^ ^1^, ^2^ ^]^ Significant research has focused on the conversion of CO_2_ into C_1_ products such as CO, CH_4_, HCOOH, and CH_3_OH.^[^ ^3^, ^4^, ^5^ ^]^ However, attention is increasingly turning toward the photocatalytic conversion of CO_2_ into C_2_^+^ compounds, such as ethylene and ethane, owing to their higher energy density, greater commercial value, and broader applicability as industrial feedstocks.^[^ ^6^, ^7^ ^]^ Despite this potential, the process remains highly challenging because of the similar reduction potentials among various multi‐electron transfer products and the substantial energy barriers involved in C–C coupling.^[^ ^8^, ^9^ ^]^

Conventional photocatalytic CO_2_ reduction often struggles to effectively facilitate multielectron processes owing to the presence of a weak and uniformly distributed interfacial electric field (IEF), which fails to adequately activate the catalytic sites.^[^ ^10^, ^11^ ^]^ However, by deliberately engineering asymmetric charge distributions or introducing specific vacancy structures within the photocatalyst, a localized electric field can be generated that significantly enhances CO_2_ activation efficiency.^[^ ^12^, ^13^, ^14^ ^]^ In particular, the formation of such localized electric fields strengthens the interaction between the photocatalyst surface and CO_2_ molecules, thereby increasing their polarization and reactivity. Unlike the conventional uniform electric field, an asymmetric charge distribution creates a strong electric field gradient on the catalyst surface. This gradient drives the accumulation of photogenerated electrons at specific active sites, increasing local electron density and promoting efficient multi‐electron transfer reactions.^[^ ^15^ ^]^ This concept has been validated through the dipole‐limiting domain field (DLDF), where enhanced localization and stronger field strength stabilize C_1_ intermediates, promote dipole–dipole interactions, and ultimately favor C–C coupling reactions.^[^ ^16^ ^]^ Although the DLDF effect has shown promise in facilitating CO_2_ activation, current research remains limited and is mainly focused on vacancy‐induced mechanisms.^[^ ^16^, ^17^ ^]^ The application of DLDF in inducing C–C coupling is still largely unexplored, and its functional role in this process is not yet fully understood. Notably, the formation of asymmetric bi‐atomic sites (especially Cu‐containing dual sites) can amplify the DLDF effect by increasing local electron density at active sites, enhancing the stabilization of C_1_ intermediates, and facilitating the formation of *CO–CO coupling intermediates.^[^ ^18^ ^]^ Cu(I) species, recognized for their favorable thermodynamic properties in C–C coupling reactions, play a key role in the efficient production of C_2_ products such as C_2_H_4_.^[^ ^19^, ^20^ ^]^ Therefore, the rational design of stable, asymmetric Cu‐based dual sites—combined with the DLDF effect—holds significant potential to enhance the photocatalytic CO_2_ reduction process and accelerate the C–C coupling reactions essential for C_2_ product formation.

According to this approach, we successfully grafted flexible ethylene diamine tetraacetic acid (EDTA) molecules onto a novel two‐dimensional Ti‐BPDC (Ti‐MOF) with abundant structural defects. EDTA, an organic ligand with multiple coordination sites, forms stable chelating structures with metal ions through its carboxyl groups and nitrogen atoms, thereby achieving strong anchoring of bimetallic atoms. In addition, the flexible framework of the EDTA molecule allows it to adapt to the heterogeneous surface sites of the metal organic framework (MOF), promoting a more uniform spatial distribution of the bimetallic species.^[^ ^3^, ^21^, ^22^ ^]^ This strategy not only effectively prevents the agglomeration and migration of metal species but also enhances the synergistic interaction between the biatomic sites. Moreover, the grafting of EDTA induces a reconfiguration of the local electronic environment on the defect‐rich Ti‐MOF surface, creating favorable conditions for the formation of electric dipoles between Cu‐containing diatomic sites.^[^ ^23^ ^]^ The resulting dipole field facilitates the activation of CO_2_ molecules by enhancing their polarization and reactivity.^[^ ^24^ ^]^

In this study, Cu and Zn were individually anchored as single‐atom centers within a Ti‐based MOF (Ti‐BPDC) via chelation with grafted EDTA ligands, to form an asymmetric bimetallic system denoted as CuZn‐E‐d‐MOF. This catalyst demonstrated an exceptional C_2_H_4_ production rate of 308.72 µmol g^−1^ h^−1^, with a remarkable electron selectivity of 98.4% toward C_2_H_4_. Notably, under the influence of the DLDF, the asymmetric Cu–Zn dual sites enabled the complete utilization of key *CO intermediates for C–C coupling, effectively suppressing CO formation as a byproduct. These findings highlight the critical role of the DLDF effect in steering the reaction pathway toward C–C bond formation, thereby enabling highly selective and efficient multicarbon product generation from CO_2_ reduction. Furthermore, density‐functional theory (DFT) calculations and experimental verification revealed that Cu(II) centers predominantly facilitated the activation and conversion of CO_2_ to C_1_ intermediates, while Cu(I) species mainly governed the dimerization process that led to C_2_ product formation. The coexistence of Cu in multiple oxidation states enabled a stepwise and synergistic catalytic process. Notably, the spatially asymmetric configuration of Cu and Zn at the dual active sites further amplified the dipole confinement field effect, enhanced local charge polarization, and accelerated the activation of CO_2_ molecules. Moreover, Zn doping effectively shortened the d–p orbital center distance, strengthened orbital hybridization, and facilitated efficient electron transfer. This synergistic modulation of the electronic structure and local field dynamics is crucial for directing the reaction pathway toward C–C coupling, highlighting the advantages of asymmetric bimetallic sites in photocatalytic systems.

## 2 Results and Discussion

### 2.1 Synthesis and Structure of CuZn‐E‐d‐MOF

**Figure 1:**
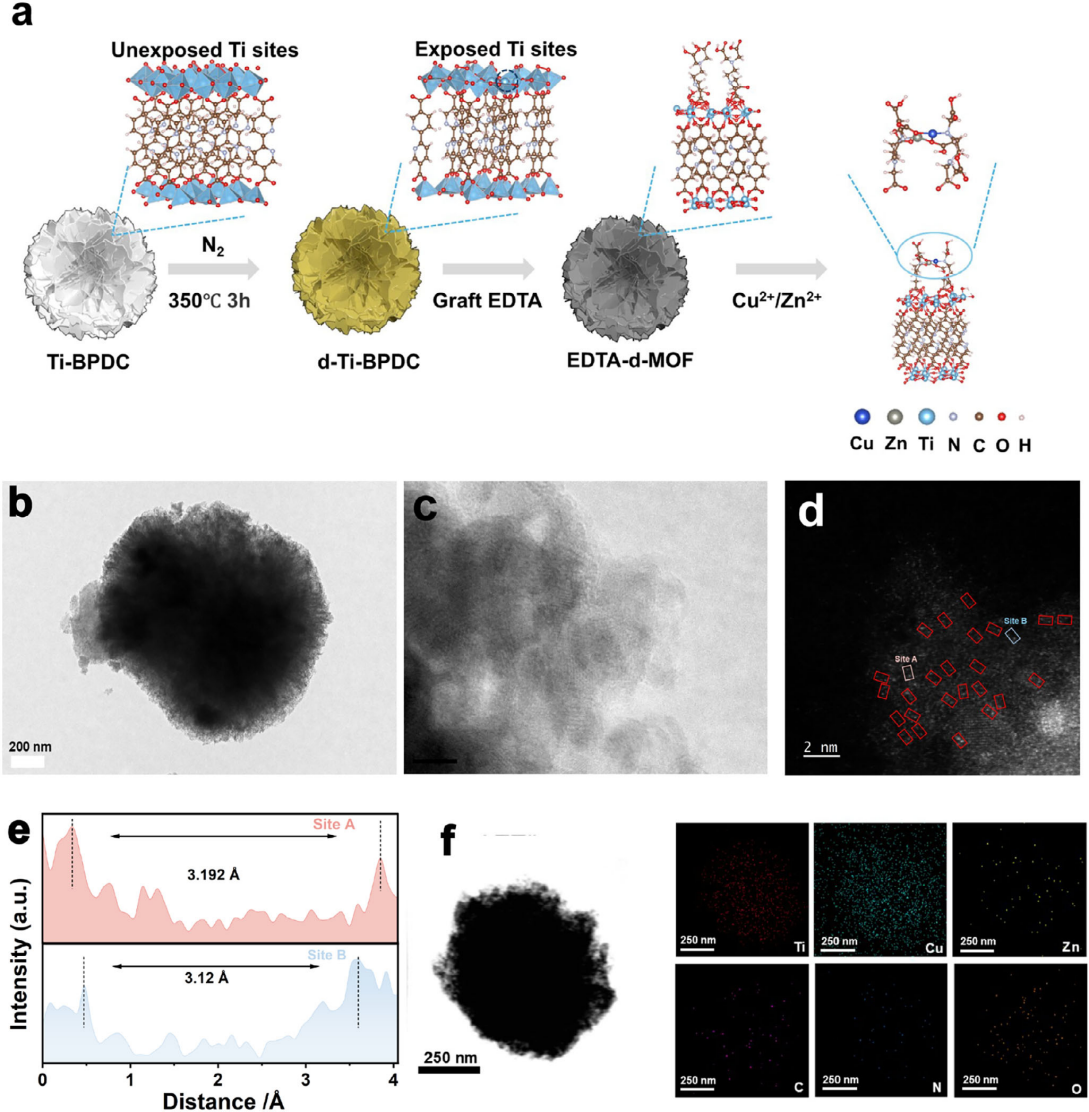
a) Synthesis process of the CuZn‐E‐d‐MOF photocatalyst. b) TEM and c) HRTEM images of CuZn‐E‐d‐MOF. d) High‐angle annular dark field scanning transmission electron microscopy (HAADF‐STEM) image, e) dual‐atom spacing measurements at site A and site B, f) EDS elemental mapping of CuZn‐E‐d‐MOF.

The synthesis of CuZn‐E‐d‐MOF is illustrated in **Figure** **1**a. Ti‐BPDC was prepared via a simple solvothermal method using H_2_BPDC and titanium tetraethoxide as precursors and has been previously reported as a promising candidate for photocatalysis.^[^ ^25^ ^]^ Following synthesis, Ti‐BPDC was thermally activated at 350 °C to remove excess thermally unstable BPDC ligands, thereby exposing a large number of Ti–oxo clusters while preserving the MOF crystal structure and porosity.^[^ ^26^ ^]^ A polymer was then grafted onto the exposed Ti–oxo clusters via a simple stirring method to yield E‐d‐MOF. Scanning electron microscopy (SEM) and transmission electron microscopy (TEM) images (Figures S1 and S2, Supporting Information) revealed that the surface of the MOF, after EDTA modification, displayed strip‐like polymeric features. In addition, the crystal structure of E‐d‐MOF was confirmed via powder X‐ray diffraction (XRD), as illustrated in Figure S3 (Supporting Information). The XRD patterns revealed that the diffraction peak positions of E‐d‐MOF closely matched those of Ti‐BPDC, indicating that the MOF crystal structure remained stable during the EDTA modification process. However, the intensity of certain diffraction peaks in E‐d‐MOF was reduced compared with those in Ti‐BPDC, which may be attributed to the introduction of the polymer reducing the exposure of some crystal surfaces or inducing a degree of lattice distortion.^[^ ^27^ ^]^ In addition, the slight broadening of the peaks suggests that EDTA modification caused microstructural changes on the MOF surface without disrupting its overall long‐range order. The successful grafting of the polymer onto Ti‐BPDC was further confirmed by Fourier‐transform infrared (FTIR) spectroscopy (Figure S4, Supporting Information). The characteristic absorption bands provided valuable insight into the structural changes following functionalization. In the spectrum of d‐Ti‐BPDC, strong absorption bands between 1500 and 1400 cm^−1^ corresponded to the asymmetric and symmetric stretching vibrations of carboxylate (‐COO^−^) groups.^[^ ^28^ ^]^ Notably, after modification to E‐d‐Ti‐BPDC, these peaks shifted and broadened (marked with *), indicating alterations in coordination and bonding environments. Additionally, the appearance of a new absorption band near 1120 cm^−1^, attributed to ‐CN stretching vibrations,^[^ ^29^ ^]^ confirmed the successful incorporation of the functional moiety. Furthermore, absorption bands observed below 700 cm^−1^, assigned to Ti–O vibrations, confirmed the presence of metal–ligand interactions within the framework.^[^ ^30^ ^]^ Compared with d‐Ti‐BPDC, E‐d‐Ti‐BPDC exhibited a weakened Ti–O peak, which further confirmed the successful grafting of EDTA onto the Ti–oxo clusters, indicating interactions between the functional groups and titanium‐based clusters. Characteristic peaks corresponding to the carboxyl and amine groups of EDTA appeared in the spectra of E‐d‐MOF, confirming EDTA functionalization. Furthermore, thermogravimetric analysis (TGA) (Figure S5, Supporting Information) showed that the thermal stability of E‐d‐MOF was slightly reduced compared with pristine Ti‐BPDC, likely owing to the decomposition of the grafted polymer at elevated temperatures. X‐ray photoelectron spectroscopy (XPS) analysis further verified the successful coordination of EDTA with the Ti–oxo clusters. The Ti 2p spectra (Figure S6, Supporting Information) exhibited a binding energy shift following polymer grafting, reflecting a strong interaction between the polymer and titanium sites. Furthermore, the N 1s and O 1s spectra confirmed the successful incorporation of N‐ and O‐ containing functional groups derived from EDTA. Nitrogen adsorption–desorption isotherms (Figure S7, Supporting Information) demonstrated that E‐d‐MOF possessed a higher specific surface area than pristine Ti‐BPDC, indicating that the polymer modification improved surface accessibility without significantly blocking the pores. Moreover, the pore size distribution shifted toward smaller pores, further validating the surface structural changes in the MOF. The electron paramagnetic resonance (EPR) spectra (Figure S8, Supporting Information) highlighted differences in spin‐active species between E‐d‐Ti‐BPDC and d‐Ti‐BPDC. The main signal observed near 3400 G corresponded to a g‐factor of ≈2.003, characteristic of unpaired electrons associated with oxygen vacancies. Notably, the E‐d‐Ti‐BPDC sample showed a significantly enhanced EPR signal intensity compared with d‐Ti‐BPDC, indicating a higher concentration of oxygen vacancies. This increase was likely due to the structural properties of EDTA, which may promote defect formation through its chelating effect on titanium sites.^[^ ^31^ ^]^ Overall, these results confirmed the successful grafting of EDTA onto the unsaturated Ti–oxo clusters within the MOF framework.

CuZn‐E‐d‐MOF was synthesized using a solvent‐assisted linker exchange method (Figure 1a), resulting in a material with a Brunauer‐Emmett‐Teller (BET) surface area of 70.1 m^2^ g^−1^ (Figure S7, Supporting Information). Transmission electron microscopy (TEM) images of the CuZn‐E‐MOF sample (Figure 1b,c) showed no visible evidence of Cu or Zn nanoparticles. In addition, XRD patterns (Figure S9, Supporting Information) revealed no characteristic peaks corresponding to metallic phases. Despite the absence of Cu or Zn nanoparticles, the presence of Cu and Zn in CuZn‐E‐MOF was confirmed via elemental analysis (Figure S10, Supporting Information). To further verify the successful synthesis of an atomically dispersed Cu‐Zn dual‐atom catalyst, aberration‐corrected high‐angle annular dark‐field scanning transmission electron microscopy (AC HAADF‐STEM) was employed. The single‐atom sites are visible in Figure 1d, highlighted by contrast differences between the metal species and the supporting material. The bright dots, marked by red squares, indicate metal pairs. Notably, the measured distances in two representative regions were 3.192 Å and 3.12 Å (Figure 1e), closely matching the 3.181 Å distance obtained from geometry optimization. Energy‐dispersive X‐ray spectroscopy (EDS) mapping in Figure 1f illustrates a homogeneous distribution of Cu, Zn, Ti, C, N, and O in the CuZn‐E‐d‐MOF. Inductively coupled plasma‐atomic emission spectrometry (ICP‐AES) measured metal contents of 11.8 wt.% Cu and 1.25 wt.% Zn (Table S2, Supporting Information), which may be related to the differences in coordination chemistry between Cu and Zn. HAADF‐STEM imaging (Figure S11, Supporting Information) further confirmed the atomic dispersion of Cu and Zn, indicating a uniform distribution of all elements throughout the catalyst. The +2 oxidation states of Cu and Zn species in CuZn‐E‐MOF were further confirmed via X‐ray photoelectron spectroscopy (XPS) (Figure S12, Supporting Information). The observed shifts and disappearance of peaks in the O 1s and N 1s spectra strongly indicated interactions between the Cu/Zn atoms and the nitrogen and oxygen atoms of EDTA (Figure S13, Supporting Information).

**Figure 2:**
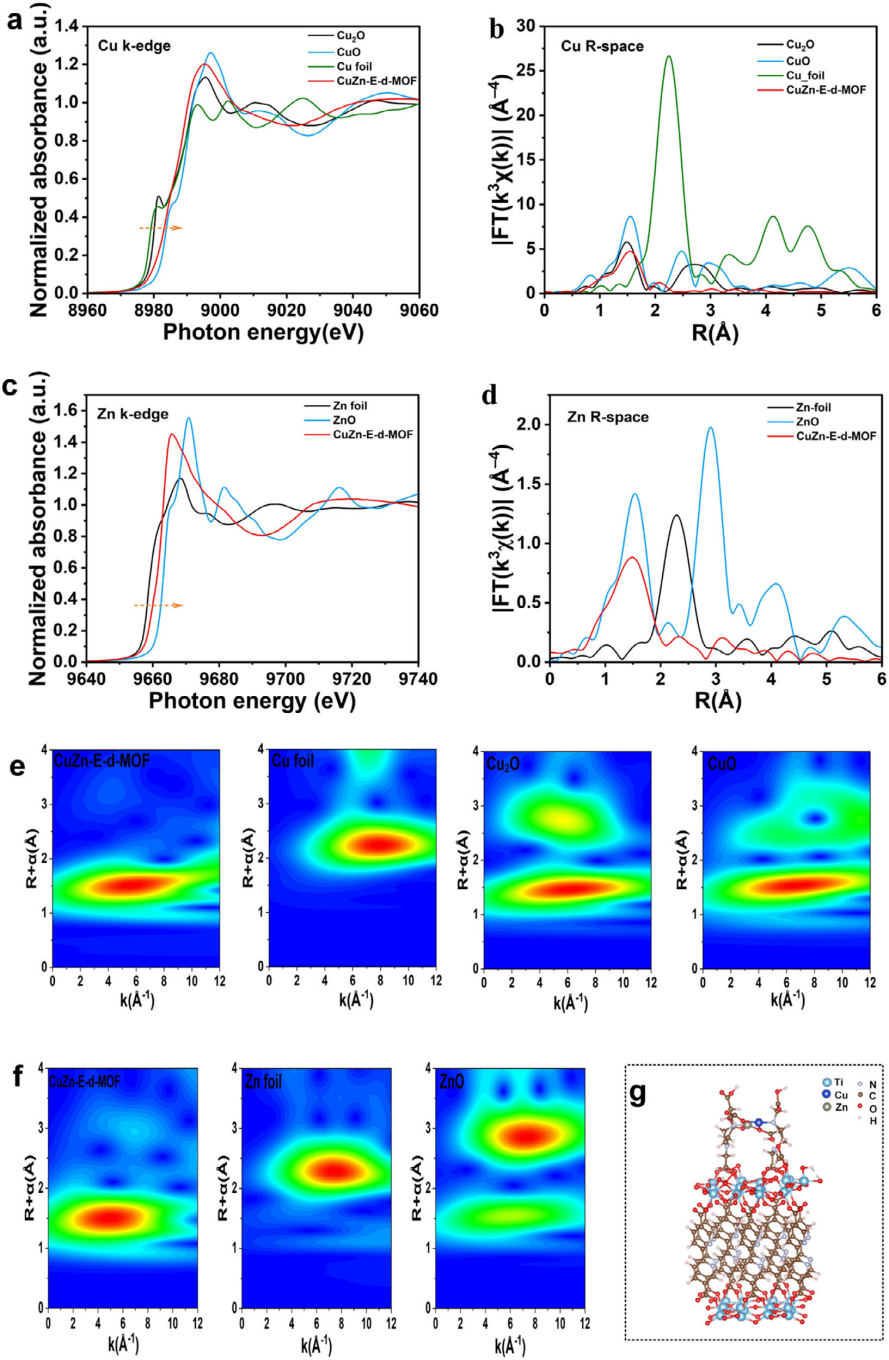
a) Normalized Cu K‐edge EXAFS spectra and b) Fourier transform k^3^‐weighted EXAFS for Cu_2_O, CuO, Cu foil, and CuZn‐E‐d‐MOF. c) Normalized Zn K‐edge EXAFS spectra and d) Fourier‐transform k^3^‐weighted EXAFS for Zn foil, ZnO, and CuZn‐E‐d‐MOF. e) Wavelet transform of the k^3^‐weighted EXAFS for Cu_2_O, CuO, Cu foil, and CuZn‐E‐d‐MOF. f) Wavelet transform of the k^3^‐weighted EXAFS for Zn foil, ZnO, and CuZn‐E‐d‐MOF. g) DFT‐calculated structural models of CuZn‐E‐d‐MOF.

X‐ray absorption spectroscopy (XAS) was used to investigate the local electronic structure and coordination environment of Cu and Zn in CuZn‐E‐d‐MOF. The X‐ray absorption near‐edge structure (XANES) spectra offer key insights into the oxidation states and bonding nature of the metal centers, while extended X‐ray absorption fine structure (EXAFS) analysis reveals details about atomic dispersion and coordination environments. As shown in the Cu K‐edge XANES spectra (**Figure** **2**a), the absorption edge of CuZn‐E‐d‐MOF was between those of Cu_2_O and CuO, indicating the coexistence of Cu^+^ and Cu^2+^ oxidation states, with Cu^2+^ as the dominant species, consistent with the XPS fitting analysis (Figure S12, Supporting Information). The Cu(II) leading edge shoulder observed ≈8970–8985 eV, closely matching the CuO reference spectrum, further confirmed a significant presence of Cu^2+^ species within the framework. Similarly, the Zn K‐edge XANES spectrum of CuZn‐E‐d‐MOF (Figure 2c) closely matched that of ZnO, confirming the presence of Zn–O coordination. Moreover, no characteristic signals indicative of metallic Zn─Zn bonding were detected, suggesting that Zn was atomically dispersed within the MOF and mainly coordinated with oxygen atoms. This conclusion was further supported by the Fourier‐transformed EXAFS spectra in R‐space (Figure 2d), where CuZn‐E‐d‐MOF showed a distinct Zn–O coordination peak similar to ZnO but lacked the Zn─Zn metallic bonding feature observed in Zn foil.

EXAFS analysis further confirmed the atomic dispersion of Cu and Zn in CuZn‐E‐d‐MOF. The Cu R‐space spectrum (Figure 2b) lacked the characteristic strong Cu–Cu scattering peak found in metallic Cu foil and instead displayed features consistent with Cu–O and Cu–N coordination. This observation indicated that Cu was present in a highly dispersed state rather than as aggregated clusters or nanoparticles. Moreover, the wavelet transform (WT) EXAFS analysis (Figure 2e,f) provided additional evidence of the local coordination environment, revealing distinct intensity distributions consistent with the coordination of Cu and Zn within the structure.

Finally, the atomic structure of CuZn‐E‐d‐MOF (Figure 2g) highlighted the coordination environment of Cu and Zn within the framework. The well‐dispersed Cu and Zn sites, along with their distinct oxidation states, indicated that the structure effectively stabilized these metal species. The corresponding EXAFS fitting results (Figure S14; Table S1, Supporting Information) revealed that both Cu and Zn were chelated by EDTA in tetra‐coordinated configurations of Cu‐N‐2O and Zn‐N‐2O. The Cu─O bond length was measured at 1.96 Å, falling between the 1.92 Å reported for Cu‐N_3_ and the 2.00 Å for Cu‐N_4_ coordination in the literature. This intermediate value is attributable to the presence of Cu atoms in a mixed valence state of Cu^2+^/Cu^+^. According to the XAS results, DFT calculations were performed to model the coordination environments of Cu and Zn in the catalyst. As illustrated in Figure 2g, the DFT‐calculated bond lengths were 1.95 Å for Cu‐N and 1.92 Å for Cu‐O in the Cu‐N‐O2 configuration, and 1.96 Å for Zn‐N and 1.92 Å for Zn‐O in the Zn‐N‐O2 configuration.

### 2.2 Photocatalytic CO_2_ Reduction Performance

**Figure 3:**
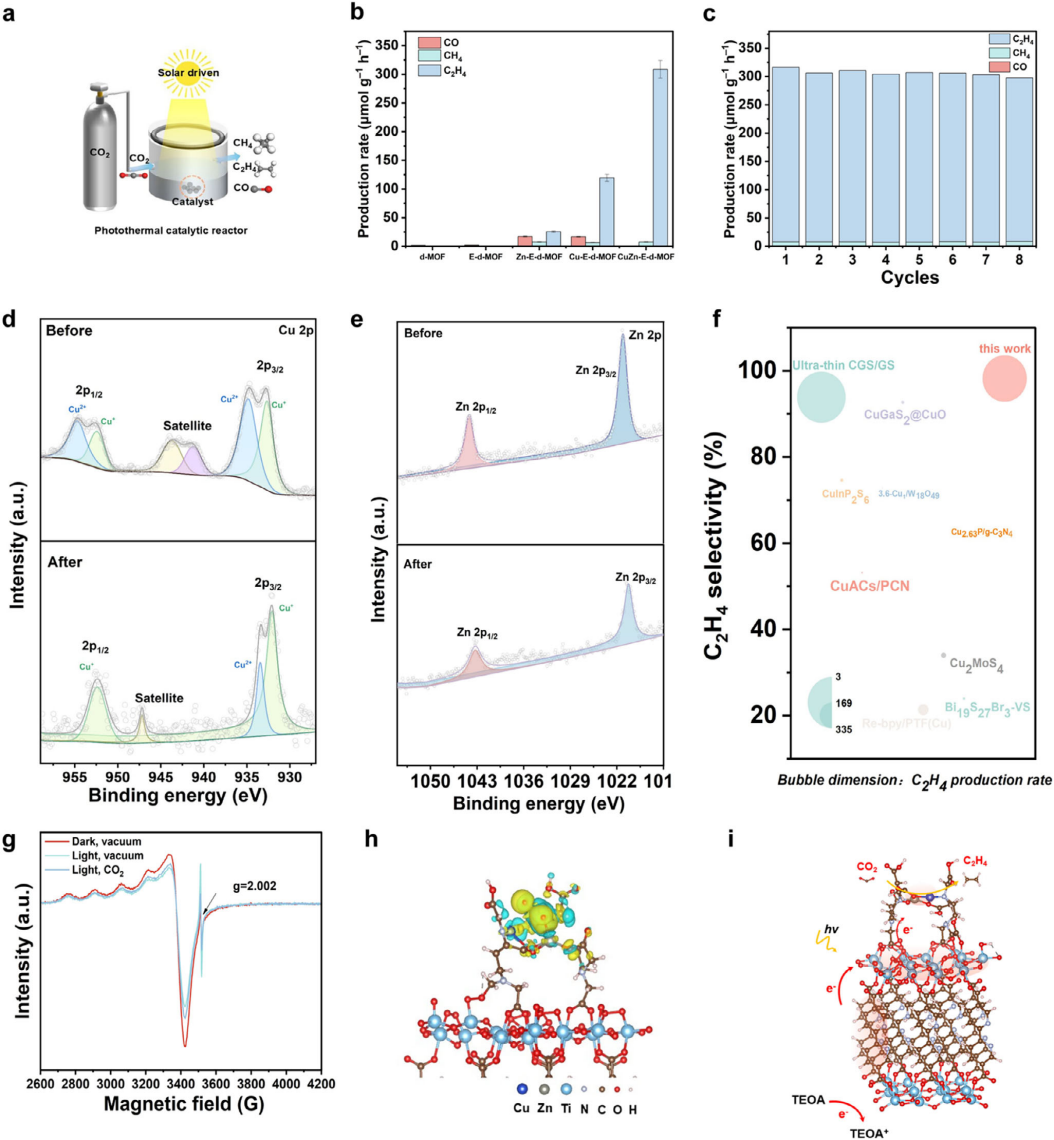
a) Schematic illustration of the photothermal catalytic reaction setup. b) Comparison of CO_2_ reduction product generation rates for different catalysts. c) Cyclic stability testing of the CuZn‐E‐MOF catalysts. d, e) Cu 2p and Zn 2p XPS spectra of the CuZn‐E‐d‐MOF sample under dark and illuminated conditions, respectively. f) Comparison of the photocatalytic performance and selectivity for CO_2_‐to‐C_2_H_4_ conversion with previously reported catalysts. g) Quasi in‐situ EPR spectra of CuZn‐E‐d‐MOF. h) Isosurfaces of charge density differences on CuZn‐E‐d‐MOF with CO_2_ molecules. Yellow indicates electron accumulation, and blue indicates depletion. i) Schematic process of charge transfer in the photocatalytic CO_2_ reaction over CuZn‐E‐d‐MOF.

The photocatalytic CO_2_ reduction reactions were conducted in a gas–solid catalytic system (**Figure** **3**a). Under these conditions, the photoreduction of CO_2_ over the CuZn‐E‐d‐MOF catalyst produced several gaseous products, including ethene (C_2_H_4_), CO, and CH_4_, with ethene being the dominant product. As illustrated in Figure 3a, although all MOFs modified with EDTA exhibited activity for ethylene production after metal ion incorporation, CuZn‐E‐d‐MOF demonstrated significantly higher activity than the others. Specifically, CuZn‐E‐d‐MOF achieved an ethene production rate of 308.72 µmol g^−1^ h^−1^, outperforming Cu‐E‐d‐MOF (119.52 µmol g^−1^ h^−1^) and Zn‐E‐d‐MOF (25.53 µmol g^−1^ h^−1^). No other gaseous products (e.g., C_2_H_6_) or liquid products (e.g., HCOOH, CH_3_OH) were detected (Figure S15, Supporting Information). Notably, CO production over the CuZn‐E‐d‐MOF catalyst was nearly undetectable. To gain deeper insights into the light utilization efficiency, the apparent quantum yield (AQY) of CuZn‐E‐d‐MOF was assessed across a range of wavelengths (Figure S16 and Table S3, Supporting Information). It achieved a remarkable maximum AQY of 2.15% at 400 nm and maintained considerable values even at longer wavelengths, manifesting its superior visible‐light harvesting capability. To unequivocally confirm that CO_2_ served as the exclusive carbon source for the products, a control photocatalytic reaction was performed using Ar in lieu of CO_2_ as the feed gas. As shown in Figure S17 (Supporting Information), no carbon‐containing products were detected in the absence of CO_2_. To further verify these findings, an isotope labeling experiment was conducted with ^13^CO_2_ under identical conditions. The results confirmed that the mass spectrometry signals at *m*/*z* 30 correspond to ^13^C_2_H_4_ (Figure S18, Supporting Information). Figure S19 (Supporting Information) presents the product and electron selectivities of the catalysts, clearly demonstrating that CuZn‐E‐d‐MOF exhibited excellent performance, with a product selectivity of 97.4% for ethylene and an electron selectivity of 98.4%.

To investigate the outstanding performance of the catalysts in photocatalytic ethylene production from CO_2_, we monitored key factors influencing C_2_H_4_ generation in real time via gas chromatography (GC9790 II) over a 3‐h period for both Cu‐E‐d‐MOF and CuZn‐E‐d‐MOF. As shown in Figure S20 (Supporting Information), *CO (* denotes adsorbed species) played a crucial role as an intermediate in ethylene formation. CO production was detectable during the first 8 min of the reaction; However, CuZn‐E‐d‐MOF exhibited almost no CO production thereafter. In contrast, Cu‐E‐d‐MOF continued to produce CO over time, while its C_2_H_4_ yield decreased as the reaction progressed, indicating that CO was desorbed from Cu‐E‐d‐MOF. The significant difference in performance between Cu‐E‐d‐MOF and CuZn‐E‐d‐MOF can be attributed to the DLDF effect. In Cu‐E‐d‐MOF, a large number of the same charge centers were distributed uniformly, whereas the asymmetric Cu‐Zn active site introduced a variety of charge centers. This asymmetry helped stabilize numerous C_1_ intermediates, enhanced dipole–dipole interactions among them, and ultimately favored the formation of C_2_ products, as illustrated in Figure S21 (Supporting Information). To further investigate this asymmetric charge distribution, we explored the effects of other bimetallic systems (Cu‐Fe, Cu‐Co, and Cu‐Ni) on photocatalytic CO_2_ reduction, leveraging the chelating capability of EDTA. As illustrated in Figure S22 (Supporting Information), all these systems exhibited improved ethylene production compared with Cu‐E‐d‐MOF.

After identifying the origin of the C_2_ product, we conducted cycling experiments to evaluate the selectivity and stability of C_2_H_4_ production. In these tests, the system was exposed to air for 3 min after every 3 h of reaction (Figure 3c). The results indicated that the catalyst maintained high catalytic activity throughout the cycles. However, a significant drop in the photocatalytic CO_2_ reduction yield was observed in the subsequent cycle when the catalyst was reused without exposure to air. (Figure S23, Supporting Information). Interestingly, during photocatalytic CO_2_ reduction, the CuZn‐E‐d‐MOF catalyst exhibited a reversible color change from blue to light yellow (Figure S24, Supporting Information), which is characteristic of a Cu(II)→Cu(I) transition. The catalyst's excellent structural integrity was further evidenced by minimal metal dissolution (Table S4, Supporting Information), confirming its stability and heterogeneous nature. This observation suggests that the surface atoms of the catalyst underwent a photo‐induced reduction during CO_2_ photoreduction. The transition from Cu(II) to Cu(I) indicated that Cu sites were actively involved in the light‐driven electron transfer process, resulting in the formation of catalytically active Cu(I) centers. These Cu(I) species are believed to play a key role in promoting the adsorption and activation of CO_2_ molecules, thereby enhancing selectivity toward C_2_H_4_ production. The reversible color change further supports the dynamic behavior of the catalytic surface under light irradiation, where the coordination environment and oxidation state of the surface atoms were continuously modulated by the photocatalytic reaction.

To elucidate the reaction mechanism, the chemical states of the CuZn‐E‐d‐MOF were investigated by using X‐ray photoelectron spectroscopy (XPS). The Cu 2p XPS spectra revealed a significant change in the copper oxidation state after 3 h of irradiation (Figure 3d). The Cu^+^/Cu^2+^ ratio shifted dramatically from 0.78 in the fresh catalyst to 7.17 post‐reaction, indicating a substantial photo‐induced reduction of Cu^2+^ centers. The selective formation of Cu^+^, which is widely recognized as the active site for C–C coupling, was further corroborated by Cu LMM Auger spectra, which exhibited a characteristic peak for Cu(I) at ≈916.5 eV without any evidence of metallic Cu(0) (Figure S25, Supporting Information). Crucially, the dynamic Cu^2+^/ Cu^+^ redox couple is essential for the entire catalytic cycle. Recycling experiments conducted under vacuum, which prevent the re‐oxidation of Cu^+^ to Cu^2+^, resulted in a significant decline in C_2_H_4_ yield. This underscores the critical role of Cu^2+^ in the initial activation of CO_2_ and the formation of key C_1_ intermediates (e.g. *CO), which are precursors for C_2_H_4_ production. In contrast to the dynamic copper centers, the Zn 2p spectra remained virtually unchanged after the reaction, confirming that zinc persists in the Zn^2+^ oxidation state (Figure 3e). This suggests a synergistic bimetallic mechanism: the redox‐active Cu sites drive the CO_2_ reduction and C–C coupling, while the stable Zn^2+^ serve as crucial electronic and structural promoters. This cooperative interplay within the EDTA‐chelated framework is responsible for the exceptional photocatalytic performance of CuZn‐E‐d‐MOF, which exhibits both higher activity and selectivity for C_2_H_4_ production than most state‐of‐the‐art catalysts (Figure 3f; Table S5, Supporting Information).

### 2.3 Electron‐Transfer Mechanism

To elucidate the mechanism of enhanced photoactivity, the electron transfer pathway within CuZn‐E‐d‐MOF was investigated. Electron spin resonance (ESR) spectroscopy revealed that upon irradiation, an intense signal at g = 2.002 emerged, attributable to electron transfer from the organic linkers to the Ti‐oxo clusters (Figure 3g; Figure S26, Supporting Information). These clusters act as an electron relay, a notion supported by work function measurements showing a spontaneous electron migration from the parent MOF framework (4.306 eV) to the electron‐accepting Cu/Zn bimetallic sites (4.587 eV). (Figures S27 and S28, Supporting Information)

This directional charge transfer was experimentally confirmed by the significant decrease of the Cu(II) ESR signal under illumination, consistent with the formation of EPR‐silent Cu(I) active centers. Upon the introduction of CO_2_, the light‐induced ESR signal changes were diminished, providing direct evidence that photogenerated electrons stored at the copper sites were effectively transferred to activate CO_2_ molecules. This multi‐step electron cascade was further corroborated by DFT calculations, which visualized charge accumulation on the Cu/Zn nodes following transfer from the Ti‐oxo clusters, priming the catalyst for CO_2_ adsorption and reduction (Figure 3h). This synergistic charge‐transfer pathway, channeling electrons from the linker via the Ti‐oxo scaffold to the Cu/Zn active sites, underpins the catalyst's remarkable C_2_H_4_ selectivity and activity.

In addition, transient photocurrent response (Figure S29, Supporting Information), electrochemical impedance spectroscopy (Figure S30, Supporting Information), and steady‐state photoluminescence (PL) measurements (Figure S31, Supporting Information) demonstrated that photogenerated electrons and holes were efficiently separated and transferred within the CuZn‐E‐d‐MOF photocatalyst. To investigate the kinetics of the separated carriers, time‐resolved photoluminescence (TR‐PL) spectroscopy was employed. Notably, the EDTA‐grafted frameworks demonstrated a significantly longer average carrier lifetime (τ_av_ = 2.66 ns) compared to the parent d‐Ti‐BPDC (see Figure S32; Table S6, Supporting Information). This observation directly confirming that the EDTA‐chelated metal sites effectively suppress charge recombination. Notably, the loading of CuZn had little effect on the absorbance and electronic band structure of the photocatalysts (Figure S33, Supporting Information), indicating that CuZn‐E‐d‐MOF possessed suitable bandgaps and appropriate band edge positions for effective CO_2_ reduction.

According to the results discussed above, a mechanistic pathway for CO_2_ photoreduction over CuZn‐E‐d‐MOF is proposed Figure 3i. Upon visible‐light irradiation, the H_2_BPDC ligands acted as light‐harvesting units, generating photoexcited electrons and holes. These excited electrons were then transferred from the ligands to the Ti–oxo clusters, which functioned as transient electron reservoirs.^[^ ^25^ ^]^ Simultaneously, triethanolamine (TEOA) quenches the photogenerated holes, serving as both a sacrificial electron donor to inhibit charge recombination and as a proton source for the reduction steps. This entire process takes place within the acetonitrile solvent, which offers a high‐solubility, aprotic environment that suppresses the competing hydrogen evolution reaction and concentrates CO_2_ at the catalytic interface. The spatial separation between the organic ligands and Ti–oxo clusters within the MOF structure helped to effectively prevent electron–hole recombination. Subsequently, electrons were transferred from the Ti–oxo clusters to neighboring Cu and Zn sites, where they were temporarily stored for further catalytic activity. When CO_2_ molecules were adsorbed onto these metal centers, the stored electrons were transferred to CO_2_, triggering its activation and reduction to C_2_H_4_. Importantly, the Cu(II) sites were partially reduced to Cu(I) during this process, demonstrating their key role in electron delivery.

### 2.4 CO_2_‐Photoreduction Mechanism

**Figure 4:**
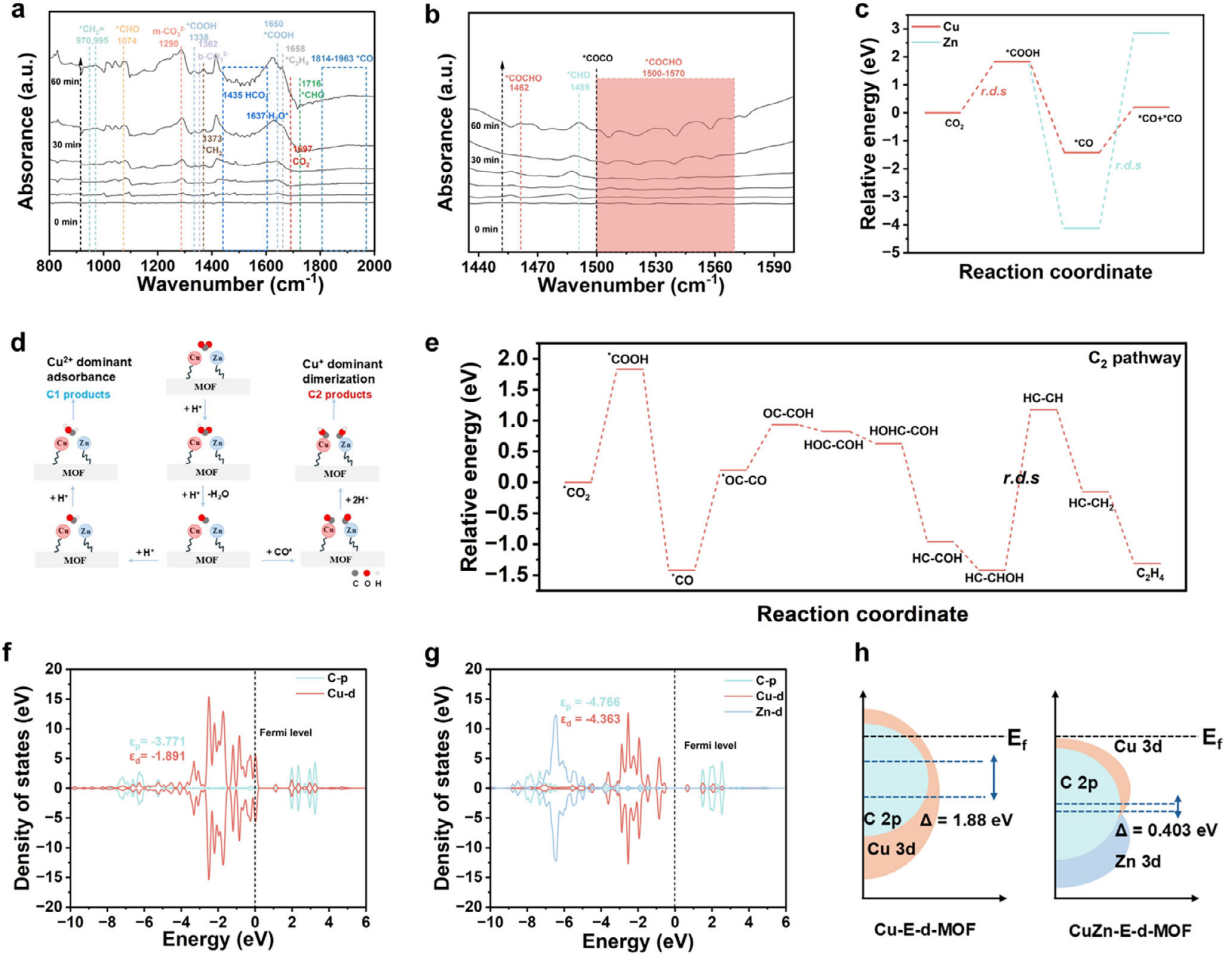
In situ, DRIFTS spectra were obtained at different irradiation times during CO_2_ photoreduction over a, b) CuZn‐E‐d‐MOF. c) Relative dimerization energy comparison at Cu and Zn sites in CuZn‐E‐d‐MOF. d) Visual summary of the C_1_ and C_2_ adsorption pathways. e) Gibbs free energy diagram for CO_2_ reduction to C_2_H_4_ over CuZn‐E‐d‐MOF. f) Density of state (DOS) calculation for *OCCO adsorbed on the Cu site in Cu‐E‐d‐MOF. g) DOS calculation for *OCCO adsorbed on the Cu and Zn sites in CuZn‐E‐d‐MOF. h) Schematic rigid band diagrams of Cu‐E‐d‐MOF and CuZn−E‐d‐MOF.

Notably, asymmetric diatomic catalysts create electronic asymmetry between the two metal centers, synergistically enhancing CO_2_ adsorption. The intrinsic dipole moments generate localized electric fields, similar to a DLDF effect, which regulate charge redistribution and thereby modulate CO_2_ adsorption and activation. As illustrated in Figure S34 (Supporting Information), the maximum adsorption energy for CO_2_ molecules occurs between Cu and Zn (Cu‐Zn) at –0.405 eV, increasing the CO_2_ adsorption capacity of CuZn‐E‐d‐MOF. To further investigate the intermediates formed during CO_2_ activation and to study the C–C coupling reaction, this work combines in situ surface‐enhanced infrared spectroscopy (in situ IR) with DFT calculations to analyze the formation and transformation of key intermediates. To elucidate the surface reaction pathways during CO_2_ electroreduction, in situ FTIR spectroscopy was conducted under CO_2_ conditions (**Figure** **4**a,b). Several carbonate species were identified on CuZn‐E‐d‐MOF, including monodentate carbonate (m‐CO_3_^2−^, 1290 cm^−1^), bidentate carbonate (b‐CO_3_^2−^, 1362 cm^−1^), and bicarbonate (HCO_3_^−^, 1435 cm^−1^), reflecting multiple CO_2_ adsorption modes.^[^ ^32^, ^33^, ^34^, ^35^ ^]^ A strong absorption peak at 1697 cm^−1^ indicated molecularly adsorbed CO_2_, confirming efficient CO_2_ activation via electron transfer from the catalyst surface.^[^ ^35^ ^]^ Importantly, the presence of *COOH intermediates was revealed by characteristic peaks at 1338 and 1650 cm^−1^,^[^ ^32^, ^36^ ^]^ followed by the emergence of *CO species between 1814 and 1963 cm^−1^, demonstrating the proton‐coupled electron transfer mechanism.^[^ ^9^, ^37^ ^]^

The simultaneous detection of *CO and *CHO intermediates at 1076 cm^−1^ and 1714 cm^−1^, respectively, provides strong evidence for a C–C coupling pathway through surface dimerization.^[^ ^33^, ^38^ ^]^ The appearance of an IR band at 1500 cm^−1^, attributed to the *OCCO intermediate, further supports this mechanism.^[^ ^39^, ^40^ ^]^ Notably, characteristic peaks associated with *COCHO species at 1462 cm^−1^ and within the 1500–1570 cm^−1^ range, as illustrated in Figure 3b, progressively increase over time under continuous illumination, indicating the steady buildup of C–C coupling products on the catalyst surface.^[^ ^9^, ^35^, ^40^ ^]^

Moreover, the presence of *CH_2_═ and *CH_2_ intermediates at 970, 995, and 1373 cm^−1^, along with a *C_2_H_4_ signature at 1658 cm^−1^, indicated that *COCHO underwent successive protonation steps, ultimately producing ethylene (C_2_H_4_) as a representative C_2_ product.^[^ ^32^, ^33^, ^38^ ^]^ These findings suggest that CuZn‐E‐d‐MOF facilitates a number of key processes including CO_2_ adsorption, activation, intermediate formation and C‐C coupling, emphasising the synergistic role of the dual active sites in directing the selective formation of multicarbon products.

The formation of C_2_H_4_ occurred through *COCO dimerization, as demonstrated by in situ IR spectroscopy and reinforced by multiple complementary experiments, aligning with previously established mechanisms.^[^ ^2^, ^41^, ^42^ ^]^ This highlighted the critical influence of the DLDF effect from the Cu–Zn dual‐atom configuration, which modulated the local electric field at the catalyst surface, stabilized key polar intermediates, and reduced the activation energy for C–C coupling. To further investigate the pivotal role of the Cu/Zn dual‐atom sites in promoting *CO dimerization during photocatalytic CO_2_ reduction to C_2_H_4_, DFT calculations were conducted. The results (Figure 4c) provided strong theoretical validation for the enhanced C_2_H_4_ selectivity observed experimentally in CuZn‐E‐d‐MOF photocatalysts. The calculated free energy profiles showed that Zn sites had a lower energy barrier for *CO formation, facilitating the initial activation of CO_2_, but faced a significantly higher barrier for *CO dimerization than Cu sites. Conversely, Cu sites favored *CO–*CO coupling, emphasizing their essential role in C─C bond formation. These findings underscored the synergistic catalytic effect of the Cu/Zn dual‐atom interface, which optimally balanced *CO generation and stabilization, thereby lowering the overall energy barrier for C_2_ product formation. Combined experimental and theoretical analyses revealed that the selectivity between C_1_ and C_2_ products was governed by the stability of *CO intermediates, which were modulated by the local valence states of the Cu‐based active sites. As illustrated in Figure 4d, Cu^+^ and Cu^2+^ facilitated distinct reaction pathways leading to C_2_ and C_1_ products, respectively. Guided by in situ IR spectroscopy, literature reports,^[^ ^42^ ^]^ and experimental insights, the reaction mechanisms and key intermediate species included in the DFT calculations are summarized in Figure S35 (Supporting Information).

Figure 4e illustrates the Gibbs free energy (ΔG) changes along the CO_2_ reduction reaction (CO_2_RR) pathway, including the activation barrier for each step. The reaction began with CO_2_ adsorption and activation on the catalyst surface, followed by a proton‐coupled electron transfer that forms the *COOH intermediate. Subsequent dehydration produced the *CO intermediate. Two adjacent *CO species then combined to form the *OC–CO intermediate, representing the critical C─C bond formation step. As established in previous studies, this *CO dimerization was key to C─C bond formation.^[^ ^42^, ^43^, ^44^ ^]^ Owing to the asymmetric dual‐atom structure inducing a dipolar confinement field, the adsorption of *CO intermediates was effectively stabilized, which lowered the energy barrier and facilitated the C–C coupling process. Following C─C bond formation, a series of hydrogenation steps occurred: *OC–CO was hydrogenated to *HOC–COH, which was further protonated to form *HOHC–COH. This intermediate then underwent rearrangement and additional hydrogenation to yield *HC–CHOH. The most kinetically demanding step was the subsequent dehydration and hydrogenation of the *HC–CHOH intermediate to generate the *HC–CH species, with an energy barrier of 2.6 eV. This step involved the removal of a hydroxyl group (‐OH) alongside the formation of a C═C double bond, resulting in an unsaturated *HC–CH structure. The strong dipolar confinement field created by the asymmetric dual‐atom configuration stabilized the *HC–CHOH intermediate, which, while beneficial for intermediate retention, also imposed a significant kinetic barrier on the dehydration and C─H bond formation steps.^[^ ^16^ ^]^ As a result, this transformation required overcoming the highest energy barrier along the entire reaction pathway and was therefore identified as the rate‐determining step (RDS). Once the *HC–CH intermediate was formed, subsequent hydrogenation readily produced C_2_H_4_ (ethylene), a thermodynamically favorable process. In contrast, within the C_1_ pathway (CO_2_→CH_4_), the hydrogenation of *CHOH intermediates to *CH via C─O bond cleavage faced prohibitively high activation free energies (ΔG = 7.68 eV), making methane formation kinetically unfeasible at mild overpotentials (Figure S36, Supporting Information). While C–C coupling initiated the formation of multi‐carbon structures, the overall reaction rate was mainly controlled by the rate‐limiting dehydration‐hydrogenation step converting *HC–CHOH to *HC–CH. The strong local dipolar confinement field helped stabilize polar intermediates such as *HC–CHOH but also raised the energy barrier for their further transformation by limiting charge redistribution and bond rearrangement.

To understand the electron sources contributing to the differing catalytic performances of Cu single‐atom catalysts (SACs) and Cu‐Zn dual‐atom catalysts (DACs), we conducted projected density of states analyses (Figure 4f,g) on the *CO–*CO coupling intermediates for both catalysts. In the Cu SAC (Figure 4f), the Cu 3d band center (ε_d_ = −1.891 eV) was above the C 2p band center (ε_p_ = −3.771 eV), resulting in a large energy difference (Δ = 1.88 eV). While this gap promoted electron donation from the metal center to the carbon species, it restricted the effective orbital hybridization necessary for C─C bond formation. Upon introducing Zn to form the DAC (Figure 4g), the combined contributions of Cu and Zn atoms shifted the d‐band center downward to (ε d = −4.363 eV). This narrowed the energy difference with the C 2p band center (ε_p_ = −4.766 eV) to Δ = 0.403 eV. The reduced gap enhanced orbital overlap between the metal center and the C intermediate, facilitating charge redistribution and C–C coupling. The energy schematic (Figure 4h) summarizes these trends, illustrating that the d‐band center of the Cu‐Zn DAC is more closely aligned with the C 2p state than that of the Cu SAC, thereby improving electronic coupling at the interface. Overall, these results highlight that tuning the d‐band center through Zn doping is an effective strategy to optimize intermediate adsorption and activation, ultimately boosting C–C coupling efficiency as well as the selectivity and catalytic activity toward C_2_ products.

This principle of d‐band engineering also elucidates why other bimetallic catalysts, despite improvements over single‐atom Cu, do not achieve the superior performance of the Cu‐Zn DAC. While the introduction of Fe, Co, and Ni modulates the electronic structure, none attain the ideal d‐p energy alignment found in the Cu‐Zn system (Figure S37, Supporting Information). For instance, the CuCo catalyst exhibits a wide d‐p energy gap of Δ = 0.93 eV, significantly deviating from the optimal 0.403 eV, which results in poor orbital hybridization and inefficient C–C coupling. Similarly, the CuFe system (Δ = 0.549 eV) does not reach the ideal electronic coupling established by Cu‐Zn. Most notably, although the CuNi catalyst's d‐p gap (Δ = 0.426 eV) is very close to that of Cu‐Zn, its overall d‐band center is situated at a considerably lower energy, likely causing the over‐stabilization of C2 intermediates. This excessively strong binding impedes the subsequent product desorption step, thereby limiting the overall catalytic turnover rate. Consequently, the exceptional performance of the Cu‐Zn DAC can be attributed to its uniquely and precisely tuned electronic structure, which creates an optimal energetic landscape for the entire catalytic cycle.

## 3 Conclusion

**Figure 5:**
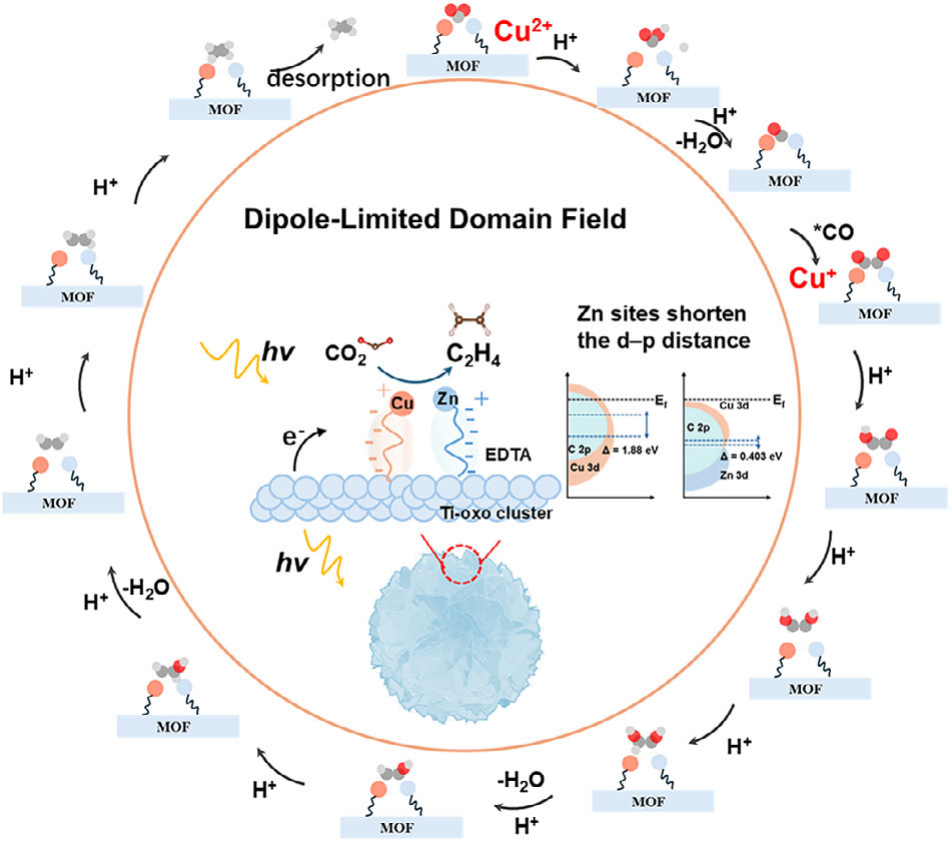
Mechanism illustrating the role of asymmetric Cu–Zn dual‐atom sites in photocatalytic CO_2_‐to‐ethylene conversion under dipole‐confinement fields.

In summary, this study demonstrates that an asymmetric Cu‐Zn dual‐atom catalyst, CuZn‐E‐d‐MOF, is a highly effective photocatalyst for the selective reduction of CO_2_ to ethylene. By engineering an asymmetric charge distribution, a localized dipole‐limited domain field (DLDF) is generated at the bimetallic sites, which enhances CO_2_ activation and promotes the multi‐electron transfer required for C–C coupling. A key aspect of this synergy is the role of Zn in precisely modulating the electronic structure of the adjacent Cu sites, specifically by upshifting the Cu d‐band center. This electronic tuning optimizes the interaction with C_1_ intermediates, thereby lowering the activation barrier for dimerization. Supported by experimental evidence and DFT calculations, we elucidate the complementary roles of the metal centers: the Cu(I) sites drive C–C coupling via *CO–*CO dimerization, while the Zn sites facilitate initial CO_2_ activation and stabilize key intermediates (**Figure** **5**). This work highlights the power of tailored diatomic configurations and electronic modulation in designing high‐performance photocatalysts for the sustainable synthesis of multi‐carbon products.

## Conflict of Interest

The authors declare no conflict of interest.

## Supporting information



Supporting Information

## Data Availability

The data that support the findings of this study are available from the corresponding author upon reasonable request.
