# Supplementary material for: Photocatalytic Asymmetric C‐C Coupling for CO2 Reduction on Cu‐Zn Bimetallic Catalysts with Dipole‐Limiting Effects
Source: Adv Sci (Weinh). 2025 Dec 8;13(11):e21670. doi: 10.1002/advs.202521670 (PMC12931159; doi:10.1002/advs.202521670)
Supplement: Supplementary file 1 — Supporting Information [file ADVS-13-e21670-s001.docx]

**Supporting Information**

Photocatalytic Asymmetric C-C Coupling for CO_2_ Reduction on Cu-Zn Bimetallic Catalysts with Dipole-Limiting Effects

Peidong Ma, Ying Yu, Fan Wen, Hongyan Guo, Boyu Li, GuoZhi Deng, Xianyang Shi, Daochuan Jiang, Ruting Huang^*[a]^

**Experimental section**

**Chemicals**

All chemicals and materials were obtained from the commercial sources and used without further purification unless otherwise required. The tetraethyl titanate, 2,2’-bipyridine-5,5’-dicarboxylic acid (H_2_BPDC) were purchased from Aladdin Company. The acetic acid, N, N-dimethylformamide (DMF) and ethanol were purchased from Aladdin Company. Ethylenediaminetetraacetic acid disodium salt，Copper chloride dihydrate and zinc chloride were purchased from Aladdin Company. Deionized (DI) water was extracted from Create Fun water purification system (18.2 MΩ cm^−1^).

**Characterization**

A cold field emission scanning electron microscope, the Regulus 8230 from Hitachi, was employed for morphology characterization, while a FEI Talos F200X G2 field emission transmission electron microscope was utilized for high-resolution surface morphology observation, energy spectrum surface scanning, and diffraction analysis to assess the material's structure and composition. A JEM-ARM200F transmission electron microscopy(TEM) high-angle annular dark-field scanning TEM images. The crystal structures and chemical valence states of the material samples were analyzed using a SmartLab X-ray diffractometer (XRD) with 2θ range from 5° to 80° and an ESCALAB 250Xi X-ray photoelectron spectrometer (XPS), the binding energy reference is the C 1s peak, with a binding energy of 284.8 eV.. Fourier infrared microscopy (Vertex80+Hyperion2000) and Raman spectroscopy (in Via-Reflex) were applied to investigate the types of functional groups and chemical bonds present in the materials. A UV-visible near-infrared spectrophotometer (Lambda 1050) was used to determine the UV-visible light response range and light absorption capacity, with the band gap width of the sample calculated through formula conversion over a test range of 200-800 nm. The photoelectric properties were assessed on a Shanghai CHI-660E electrochemical workstation. Thermogravimetric analysis (TGA) was conducted using a Mettler Toledo TGA/DSC 3^+^, Switzerland, in a nitrogen atmosphere, covering a temperature range from 30 to 800°C, and the differential curve of TGA-DTG was obtained. A comparative study of free radicals under dark and light conditions was performed using a Bruker EMXPLUS paramagnetic resonance spectrometer in Germany. Photocatalytic experiments were conducted with an online photocatalytic reaction system from Beijing MerryChange Technology Co., Ltd, and the products CO and CH_4_ were analyzed using gas chromatography connected with the Fuli GC9790Ⅱ. The instrument used for ^1^H NMR spectroscopy is JNM-ECZR400, operating at a frequency of 400 MHz. The deuterated solvent used is D_2_O, with a reference peak at 4.79 ppm. The instrument model used for ICP-AES testing is ICAP 7400. Photoluminescence spectroscopy is acquired with a steady state fluorescence and phosphorescence spectrometer (FluoroMax 4P) at an excitation wavelength of 330 nm.

**Synthesis of Ti-BPDC with defects:** Ti-BPDC is synthesized using a simple one-pot solvothermal method. In a 100 mL beaker, H_2_BPDC (100 mg, 0.41 mmol) is dissolved in a mixed solution of DMF (30.0 mL) and acetic acid (1.0 mL), sonicated for 10 min before adding tetraethyl titanate (0.13 mL, 0.624 mmol). After an additional 15 min sonication, the solution is transferred into a 100 mL Teflon-lined stainless-steel container, heating at 150 °C for 72 h.[1] After the solvothermal reaction, the formed nanoparticles Ti-BPDC were obtained by centrifugation at 7800 rpm for 15 min and further washed with DMF, methanol, and ethanol repeatedly. Further, the as-synthesized Ti-BPDC was immersed in 10 mL of 1 M HCl solution for 30 min. After centrifugating and washing with water, DMF, and methanol, the obtained particles were dried under vacuum at 80 °C. Then the samples were heated to 350 °C at a rate of 5 °C min^−1^ under Ar atmosphere and kept for 2 h in a tubular furnace. After the temperature naturally cooled to the room temperature, the activated Ti-BPDC with unsaturated Ti sites (Ti-BPDC-d) was obtained.[2]

**Preparation of EDTA-2Na aqueous solution (0.5 M)：**Take 186 g EDTA-2Na to deionized water, add sodium hydroxide in between to adjust the pH to neutral, and volume to 1L.

**Synthesis of E-d-Ti-BPDC：**The d-Ti-BPDC was reacted in EDTA-2Na solution (0.5 M) at 60 °C for 24h, after which it was washed three times with deionized water and dried at 60 degrees Celsius for 12h.

**Synthesis of** **CuZn-E-d-MOF：**Cu^2+^ and Zn^2+^ ions were bound to E-d-Ti-BPDC by a simple adsorption and chelation process. Specifically, 100 mg of E-d-Ti-BPDC was dispersed in aqueous solution containing CuCl_2_ 2H_2_O (10mL，1mM) and ZnCl_2_ (10mL，1mM) and the reaction was stirred at 50 °C for 12 hours. The product was washed with water and centrifuged, then dried under vacuum at 60 °C to obtain CuZn-E-d-MOF.

**Synthesis of Cu-E-d-MOF：**Following a similar synthetic procedure to that of CuZn-E-d-MOF,the Cu-E-d-MOF was prepared by using CuCl_2_ 2H_2_O (10mL，1mM), without any other metal salt.

**Synthesis of Zn-E-d-MOF:** Following a similar synthetic procedure to that of CuZn-E-d-MOF,the Cu-E-d-MOF was prepared by using ZnCl_2_ (10mL，1mM), without any other metal salt.

**Apparent quantum yield (AQY) calculation：**

The AQY is calculated as follow:

$$AQY = \frac{N_{e}}{N_{p}} \times100\%= \frac{MN_{A}}{\frac{SPt}{h\cdot\frac{c}{\lambda}}} = \frac{(2\times n_{CO}+8\times n_{CH4}+12\times n_{C2H4})N_{A}hc}{SPt\lambda} \times100\%$$

*N_e_* is the number of reaction electrons, *N_p_* is the number of incident photons, *M* is the amount of molecules, *N_A_* is Avogadro’s constant (6×10^23^ /mol), *h* is the Planck constant (6.626×10^-34^ J s), *c* is the speed of light (3×10^8^ m/s), *S* is the irradiation a rea (2 cm^2^), *P* is the intensity of the irradiation (420 nm: 1.76 mW cm^-2^; 450 nm: 2.15 mW cm^-2^; 520 nm: 2.79 mW cm^-2^; 600 nm: 2.73 mW cm^-2^), *t* is the photoreaction time (3600 s), and *λ* is the monochromatic light wavelength (420, 450, 520 and 600 nm).

**Photocatalytic CO_2_ reduction measurements:** Here, 5 mg of photocatalyst powder were mixed in a 6 ml solution containing 3 ml of acetonitrile, 0.5 ml of TEOA and 2.5 ml of H_2_O in a 250-ml quartz reaction cell. To prevent excessive temperature rise from prolonged light exposure, a circulating cold water system was installed in the reactor pipeline to maintain the reaction at room temperature (25°C). After setup, the reactor was evacuated using a vacuum pump and repeatedly flushed with high-purity CO_2_ (99.999%) to ensure that only CO_2_ gas was present in the reaction setup. During the reaction, magnetic stirring was employed to ensure uniform mixing. A 300 W xenon lamp (Beijing MerryChange Technology Co., Ltd.) was utilized as the light source, delivering visible light irradiation within the range of 420–760 nm. The lamp was positioned 20 cm above the reactor, yielding a light power density of 148.4 mW cm^-2^ at the reaction surface. The liquid products were detected by the ^1^H-NMR (JNM-ECZR600 400MHz spectrometer) and gas chromato-graph (FL GC9790II). During the reaction, the collected gaseous products were analyzed by a gas chromatograph equipped with a flame-ionized detector (FID) and thermal conductivity detector (TCD). The ethene selectivity was calculated by the equation as follows:

$Electron selectivity\left( \% \right)=\frac{{\mathrm{mole} C}_{2}H_{4}*12e^{-}}{\sum(\mathrm{mole} \mathrm{products} \left( \mathrm{CO}、CH_{4}、C_{2}H_{4} \right)*number of electrons)}*100\%$ (1)

$Products selectivity\left( \% \right)=\frac{{mole C}_{2}H_{4}}{\sum(mole products \left( \mathrm{CO}、CH_{4}、C_{2}H_{4} \right))}*100\%$ （2）

**Photoelectrochemical measurements.** The CHI760E electrochemical workstation (Shanghai Chenhua Instrument Co., Ltd, China) was used to conduct photoelectrochemical measurements in 0.5 mol L^−1^ Na_2_SO_4_ solution with a 300 W Xe lamp light source. The lamp was positioned 20 cm above the reactor, resulting in a light power density of 148.4 mW cm^-2^ at the reaction surface . A three-electrode experimental system contained the counter electrodes (Pt foil), reference electrodes (Ag/AgCl) and working electrodes (as-prepared catalysts). The photocurrent was performed by intermittent illumination with 0.5 V bias. Electrochemical impedance spectra were measured at open circuit potential. Mott–Schottky plots were measured at the frequencies of 500, 1000 and 1500 Hz.

**XAFS measurement.** Cu and Zn K-edge XAFS analyses were performed with Si(111) crystal monochromators at the BL14W Beam line at the Shanghai Synchrotron Radiation Facility (SSRF) (Shanghai, China). Before the analysis at the beamline, samples were placed into aluminum sample holders and sealed using Kapton tape film. The XAFS spectra were recorded at room temperature using a 4-channel Silicon Drift Detector (SDD) Bruker 5040. Cu and Zn K-edge extended X-ray absorption fine structure (EXAFS) spectra were recorded in transmission/fluorescence mode. Negligible changes in the line-shape and peak position of Cu and Zn K-edge XANES spectra were observed between two scans taken for a specific sample. The XAFS spectra of these standard samples were recorded in transmission mode. The spectra were processed and analyzed by the software codes Athena.

**DRIFTS test.** The in situ DRIFTS analysis was performed on the Bruker INVENIO S FT-IR spectrometer equipped with an in situ diffuse cell (Harrick). A certain amount of photocatalyst was placed in an in situ cell. Each sample was purged with Ar (50 mL min-1) at 120 ℃ for 0.5 h, and then the temperature naturally dropped to room temperature. The background and reference IR were collected. When the reactor was cooled to 30 ℃, a mixture of CO_2_ (5 mL min^-1^), Ar (25 mL min^-1^) and trace H_2_O vapor was introduced into the reactor for about 1 h. The background spectrum was recorded in the range 800–2000 cm^-1^ after the adsorption/desorption of CO_2_ and H_2_O on the photocatalyst reached equilibrium. IR was collected every 3 min in the first min, and data were collected every 5 min in the latter part of the time. Finally, the IR spectra were recorded in real time under the irradiation of a 300 W Xe lamp.

**Computaational details****.** The first-principles[3] based on the density functional theory (DFT) calculations was performed within the generalized gradient approximation (GGA) using the Perdew-Burke-Ernzerhof (PBE)[4] formulation. The projected augmented wave (PAW) potentials[5] were selected to describe the ionic cores and the valence electrons are taken into account using a plane wave basis set with a kinetic energy cutoff of 520 eV. The electronic energy was considered to be self-consistent when the energy change was smaller than 10^−5^ eV. A geometry optimization was considered convergent when the energy change was smaller than 0.05 eV Å^−1^. The Brillouin zone integration is performed using 2×2×1 Monkhorst-Pack k-point sampling. Finally, the adsorption energies (*E*_ads_) were calculated as *E*_ads_= *E*_ad/sub_ -*E*_ad_ -*E*_sub_, where *E*_ad/sub_, *E*_ad_, and *E*_sub_ are the total energies for the optimized adsorbate/substrate system, the adsorbate in the structure, and the clean substrate, respectively. The free energy was calculated using the equation:

*G*=*E*_ads_+ZPE-TS （3）

where *G*, *E*_ads_, ZPE and TS are the free energy, total energy from DFT calculations, zero point energy and entropic contributions, respectively, where T is set to 298.15K. For the excited state structures, full optimization is performed by applying electrons to the system.

**Supplementary Figures and Tables**


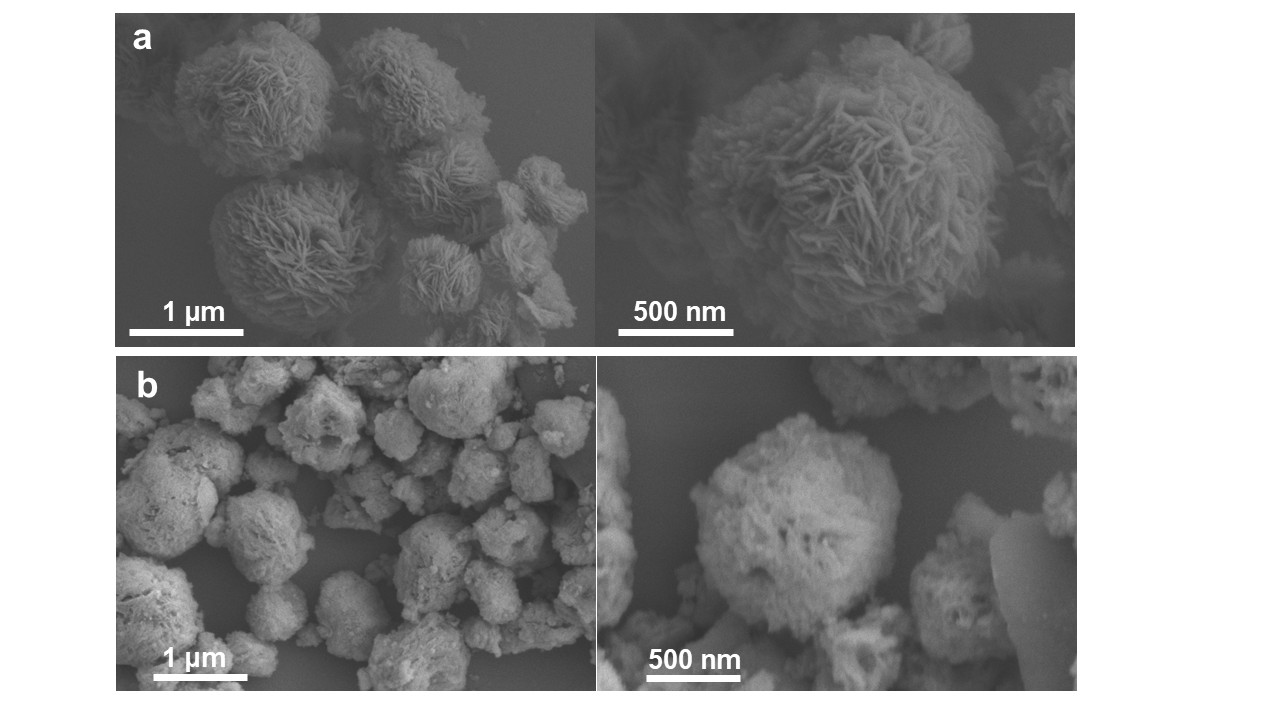


**Figure S1:** SEM image of (a) d-Ti-BPDC and (b) EDTA-d-MOF.


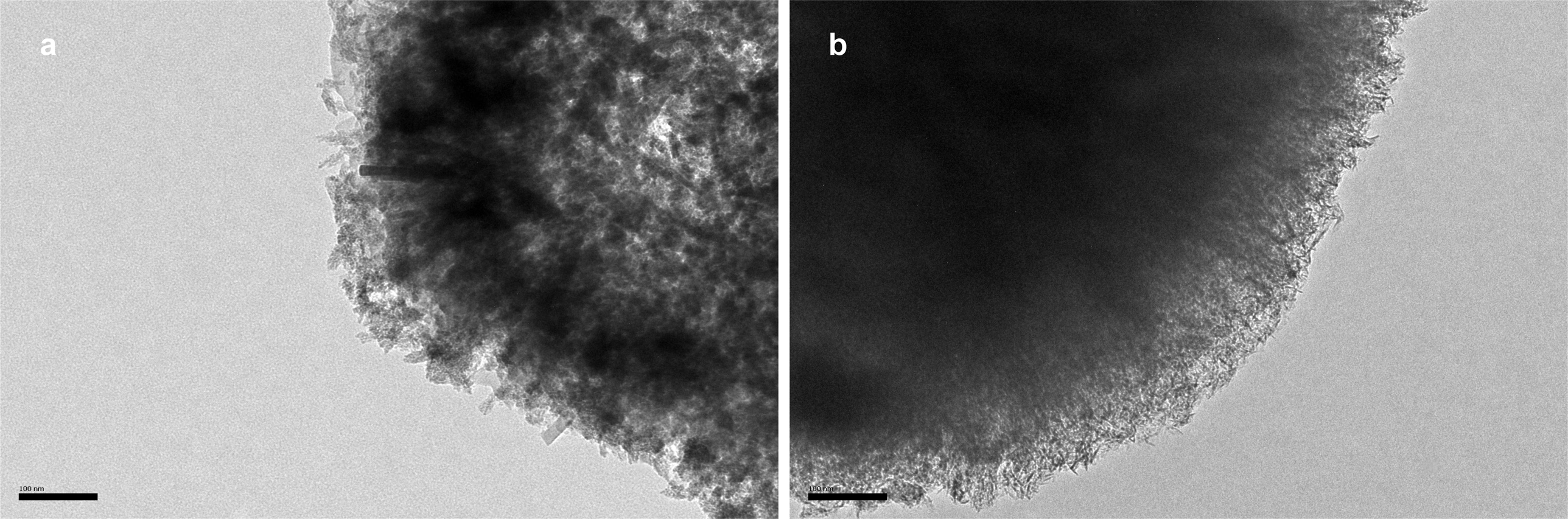


**Figure S2:** TEM image of (a) d-Ti-BPDC and (b) EDTA-d-MOF.


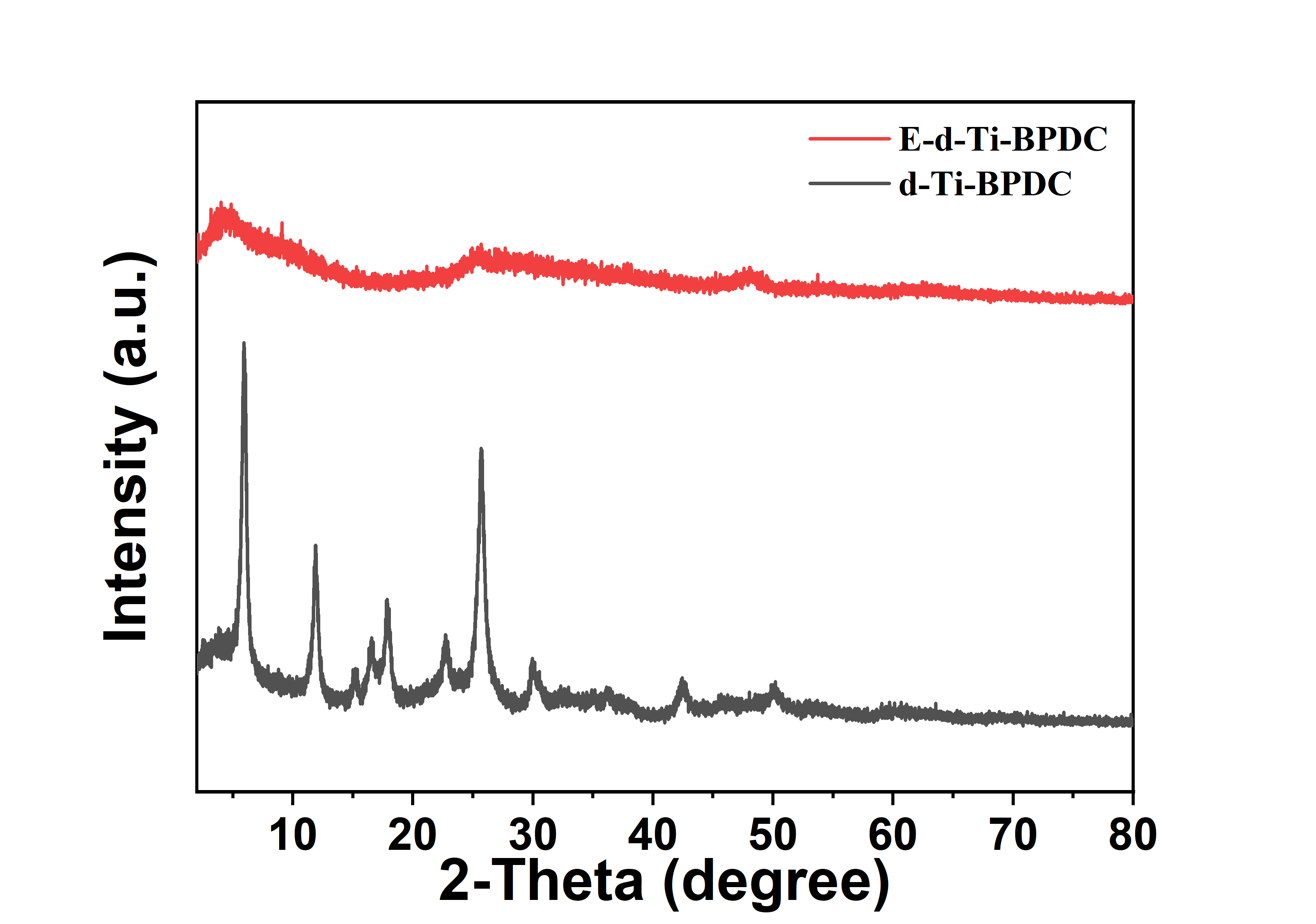


**Figure S3:** XRD patterns of d-Ti-BPDC and EDTA-d-MOF.


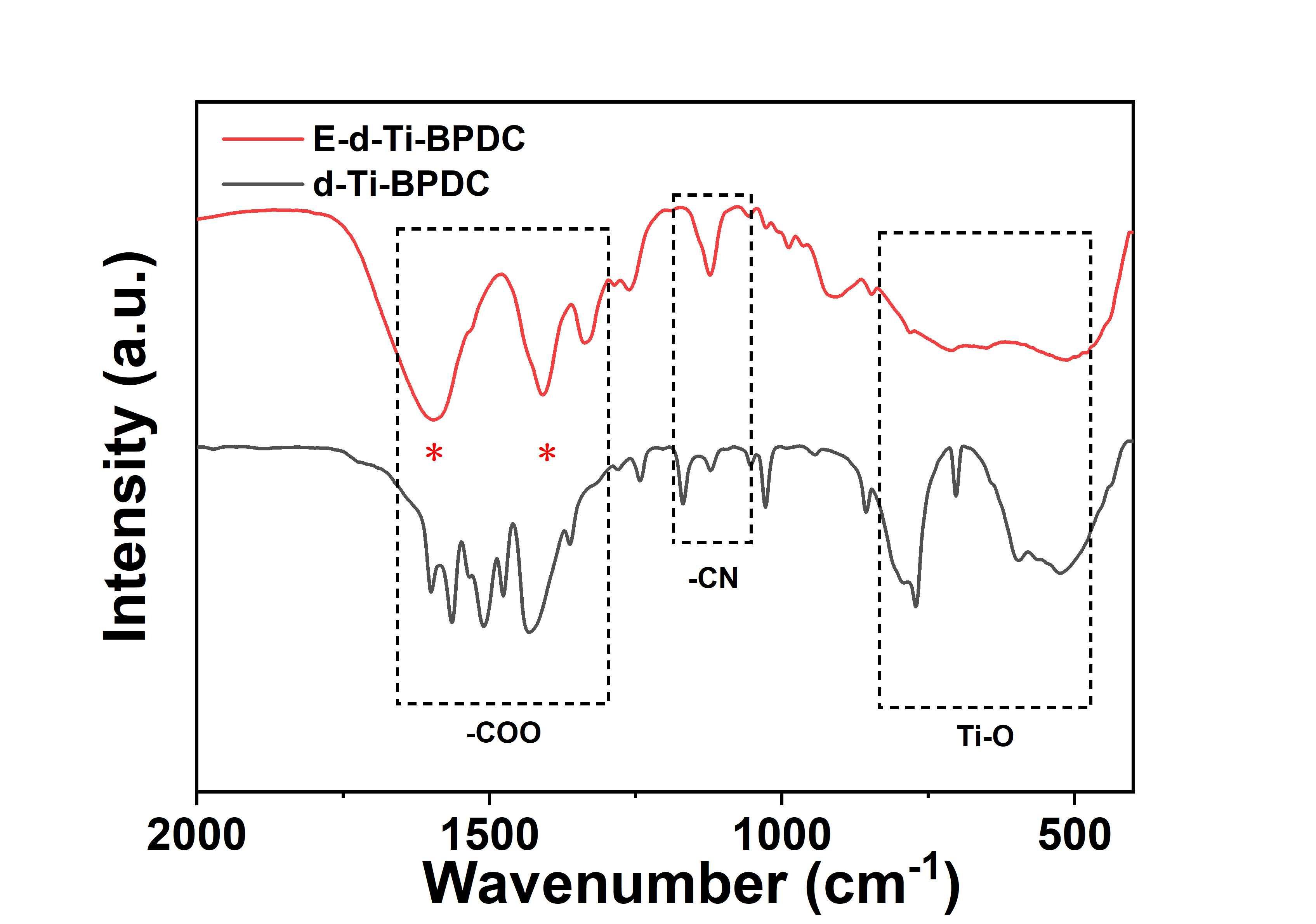


**Figure S4:** FTIR-spectra of d-Ti-BPDC and EDTA-d-MOF.

**
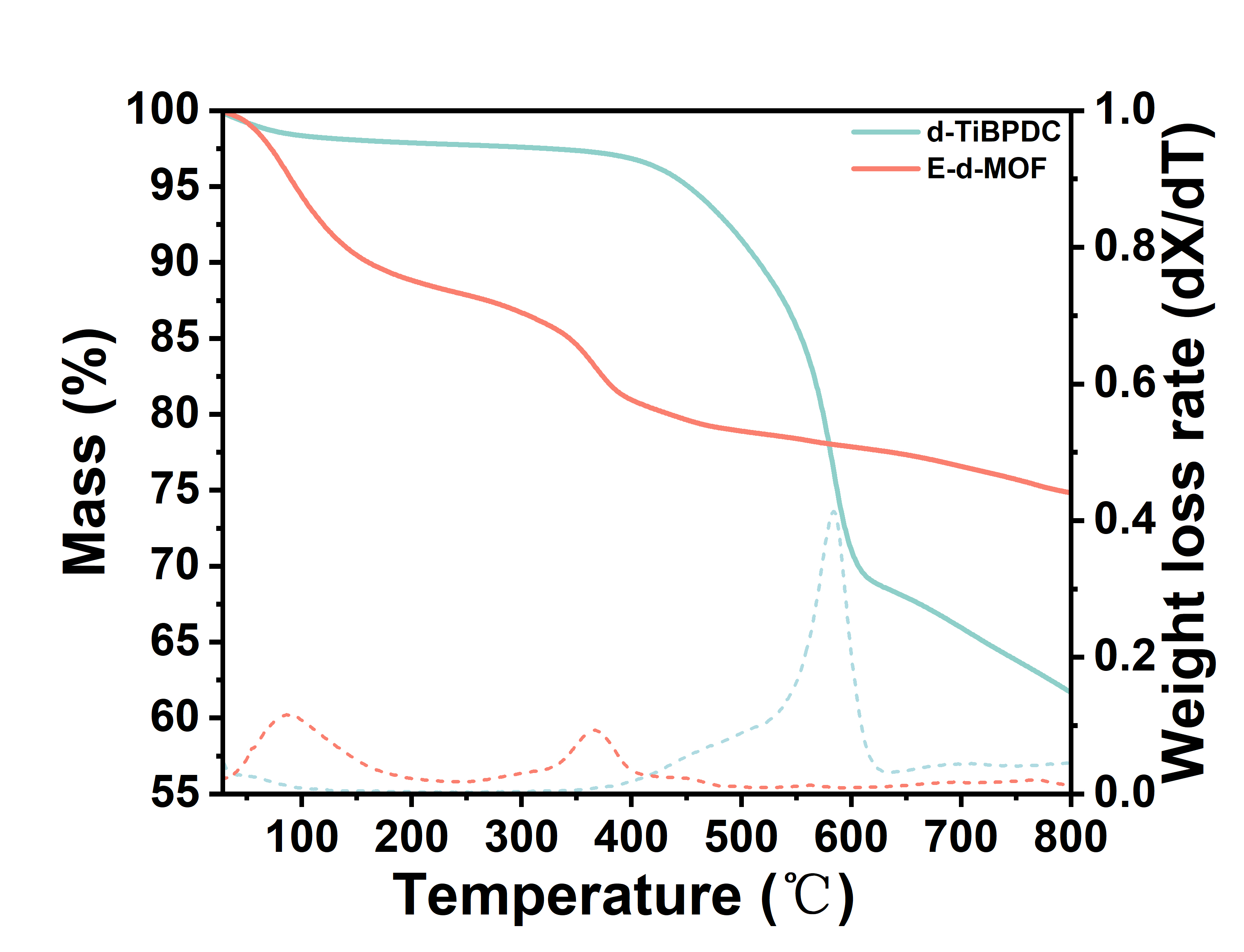
**

**Figure S5:** TG (left vertical, solid) and DTG (right vertical, dashed) curves.


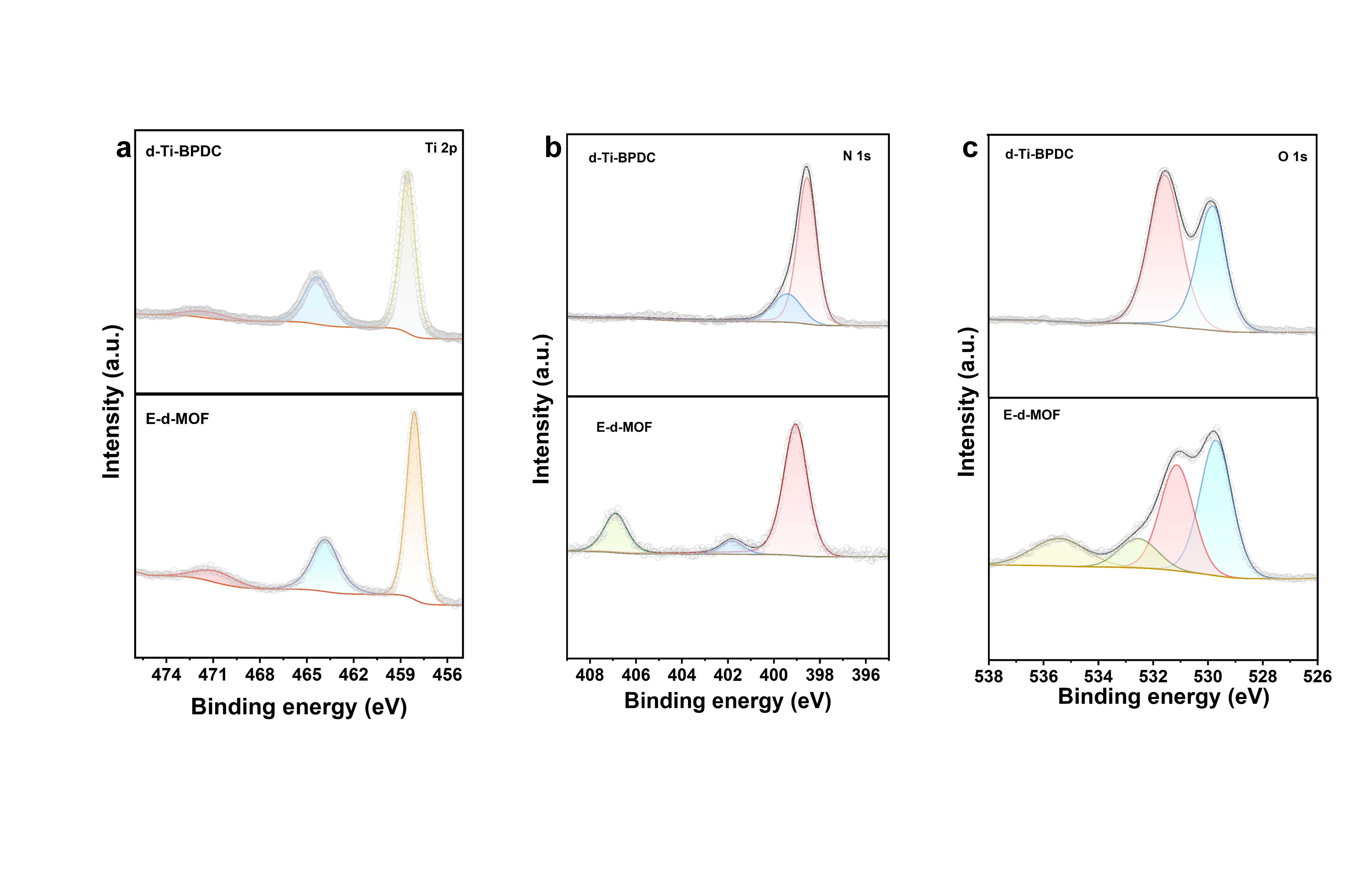


**Figure S6:** XPS spectra for Ti 2p ,N 1s and O 2p of d-Ti-BPDC and E-d-MOF.

**
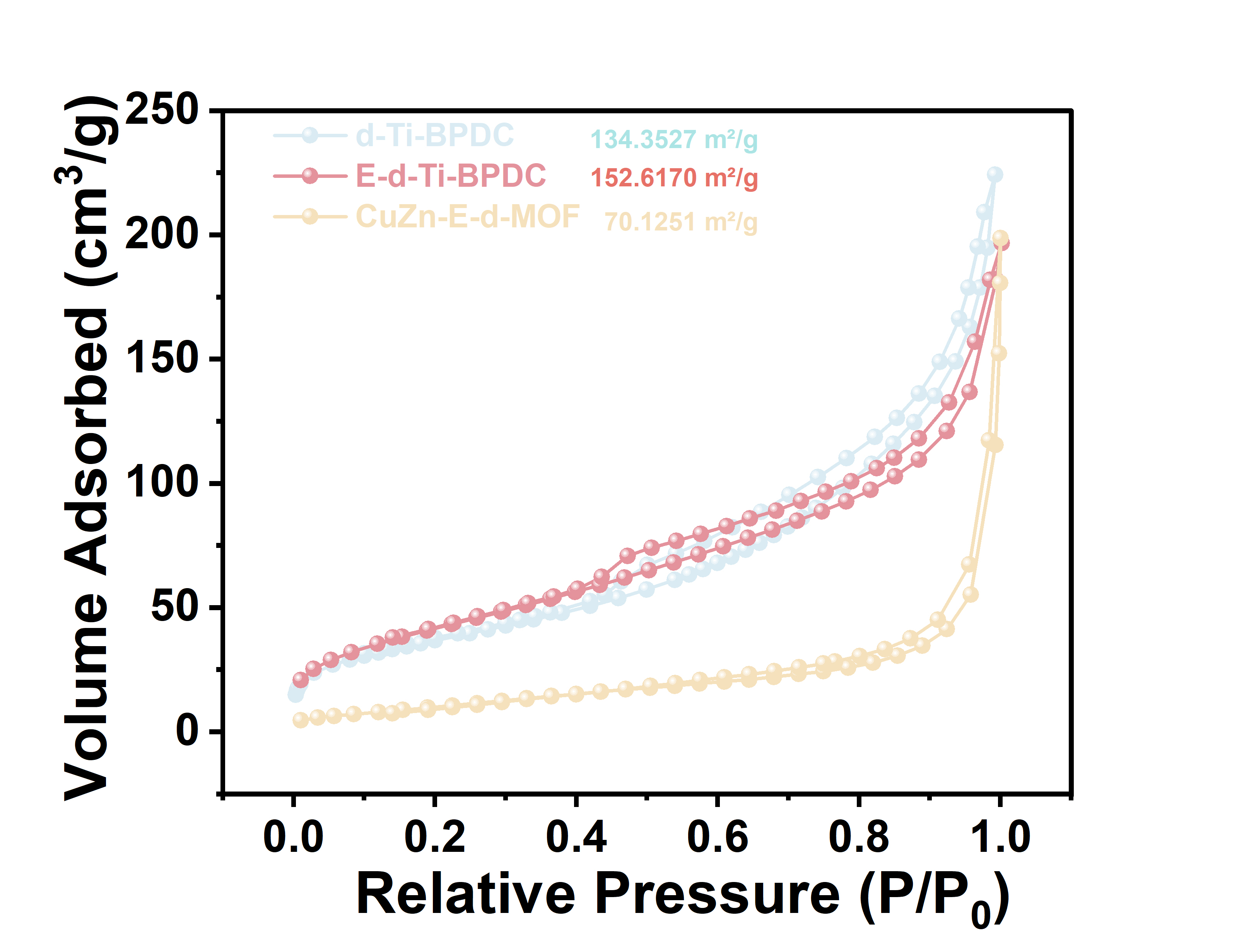
**

**Figure S7:** N_2_ sorption isotherms. N_2_ sorption isotherms of d-Ti-BPDC, E-d-Ti-BPDC，CuZn-E-d-MOF at 77 K.


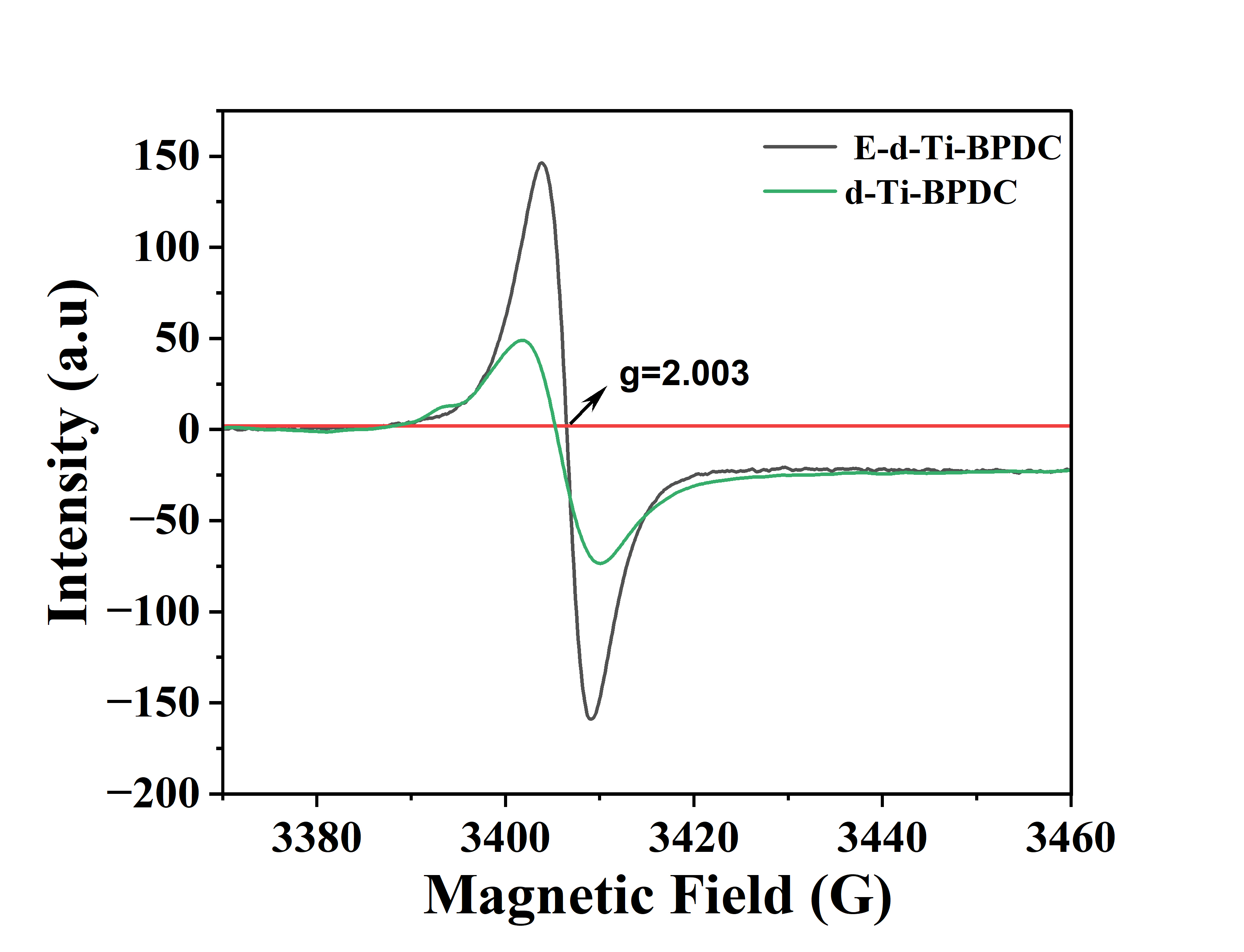


**Figure S8:** EPR spectra of d-Ti-BPDC and E-d-MOF.


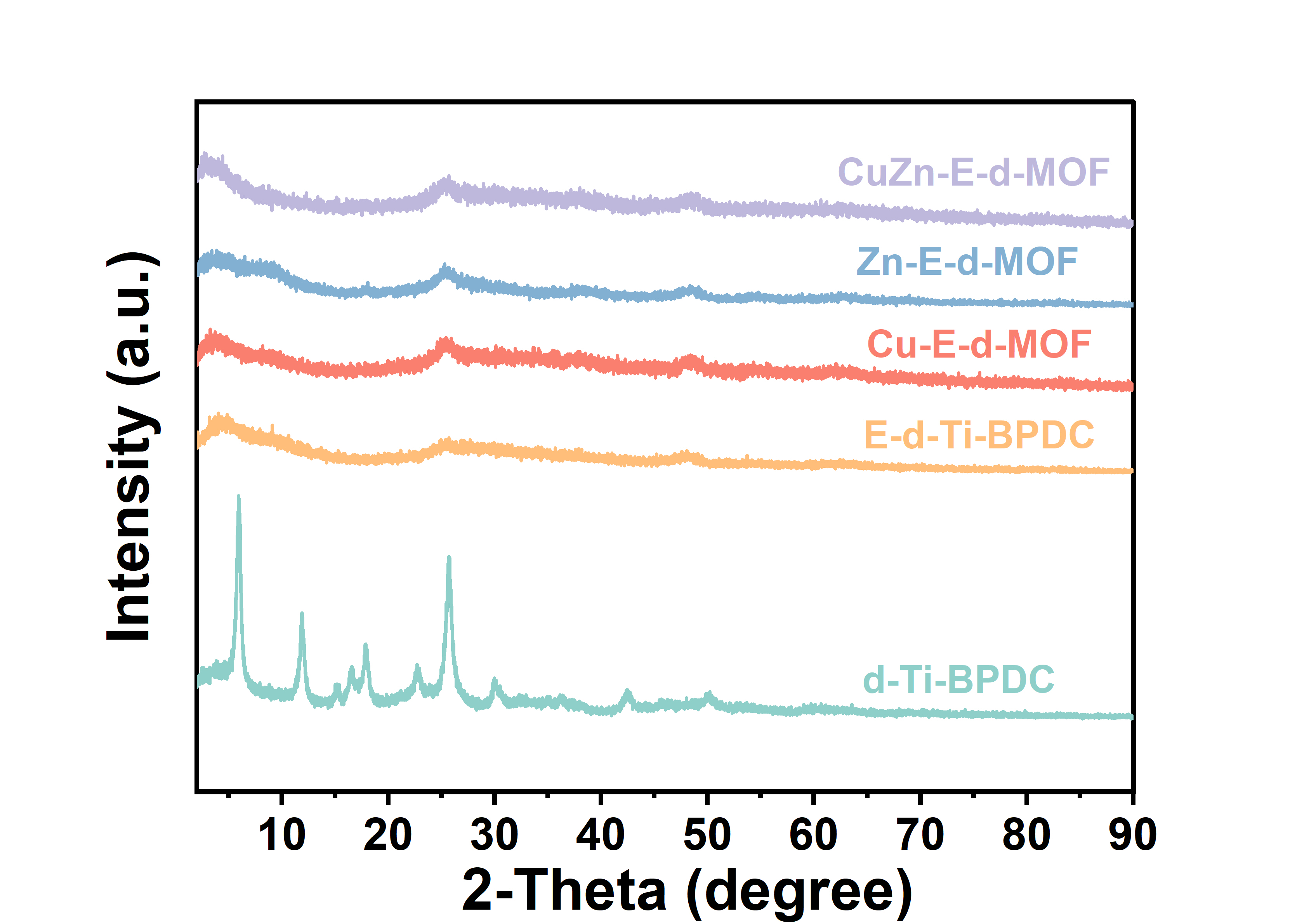


**Figure S9: Powder XRD patterns.** Powder XRD patterns of experimental d-Ti-BPDC, E-d-Ti-BPDC, Cu-E-d-MOF, Zn-E-d-MOF and CuZn-E-d-MOF, demonstrating their very similar crystalline structures.


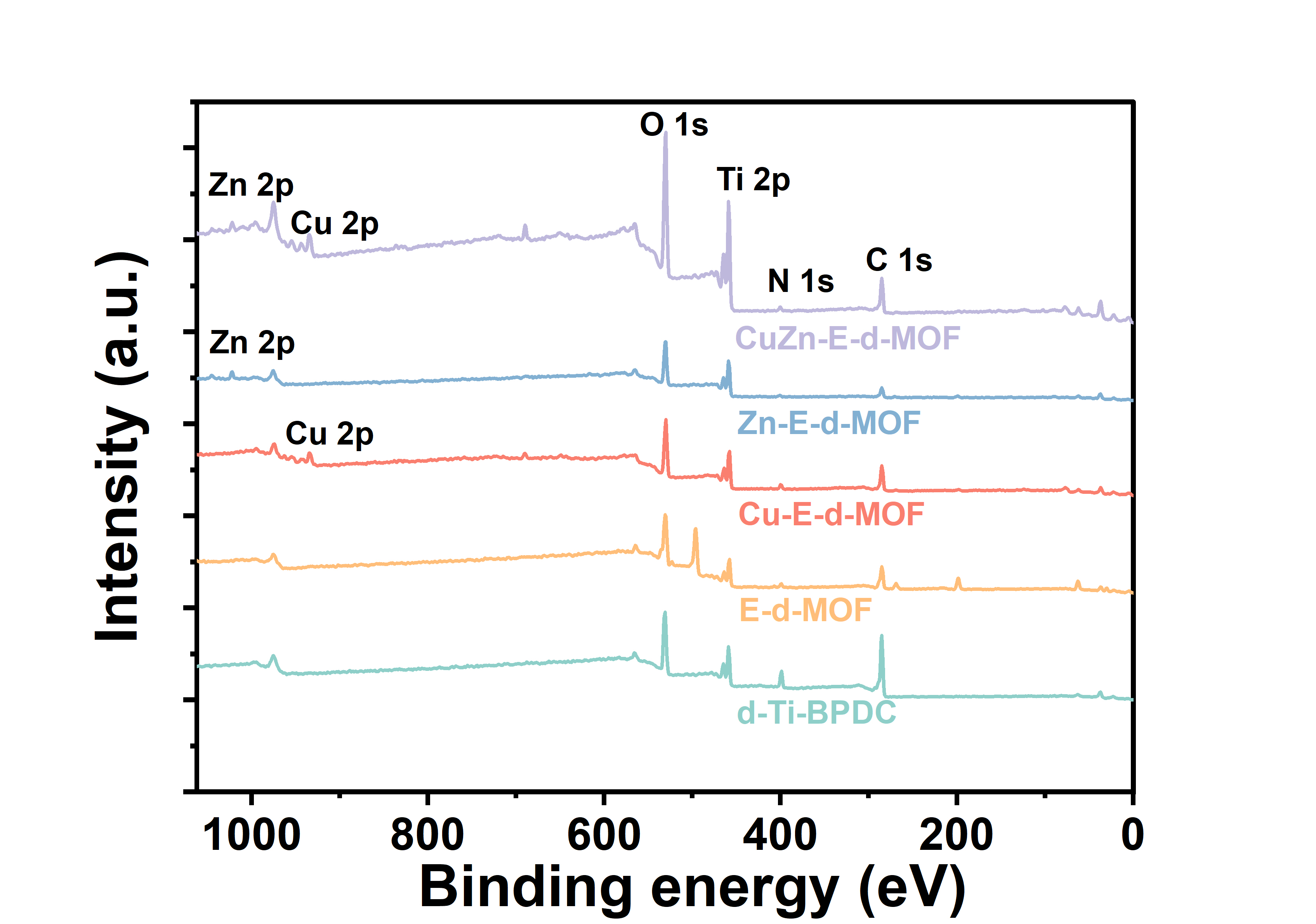


**Figure S10: XPS full spectra.** XPS spectra of d-Ti-BPDC, E-d-Ti-BPDC, Cu-E-d-MOF, Zn-E-d-MOF and CuZn-E-d-MOF, supporting the existence of Cu and Zn in the catalysts.


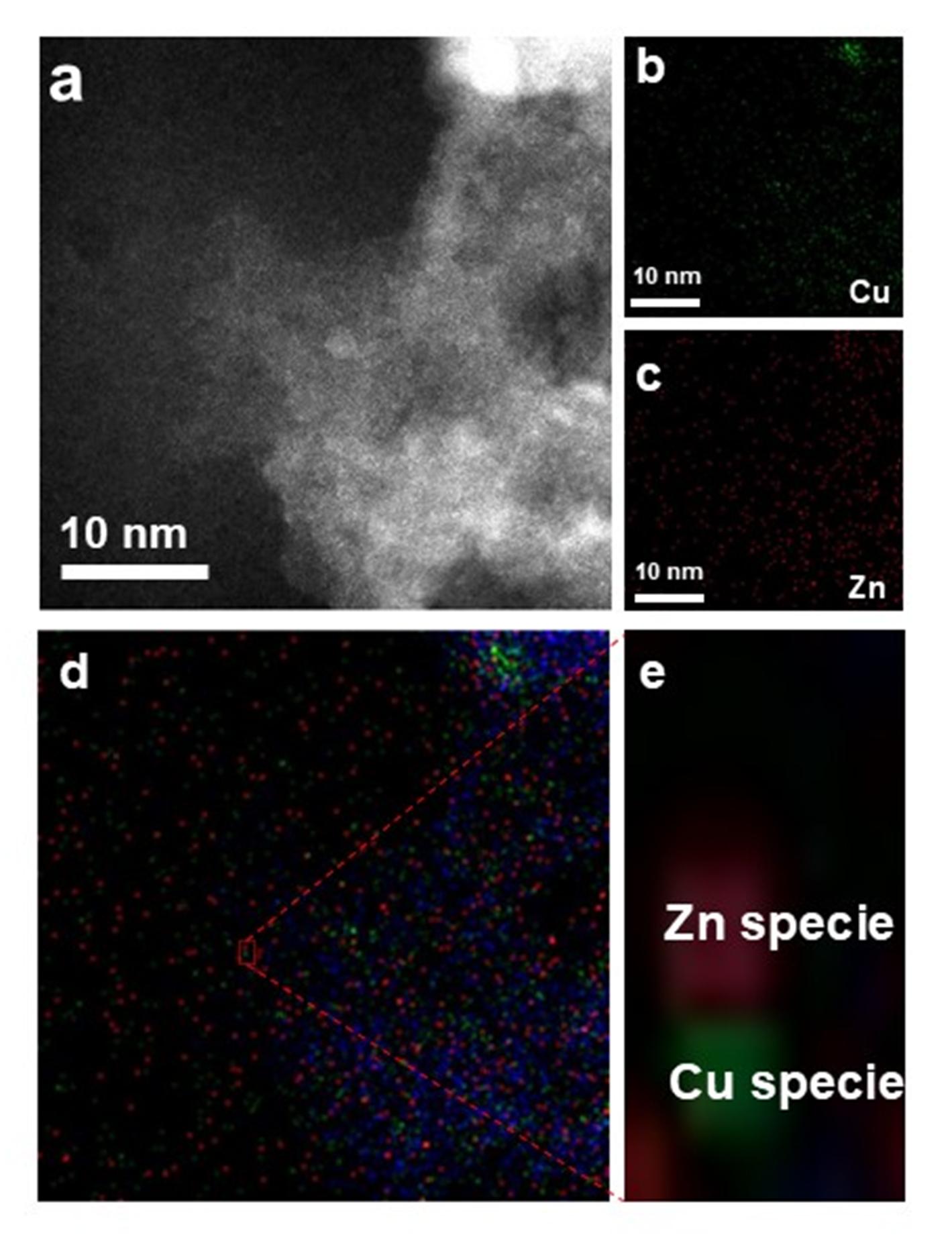


**Figure S11:** The dispersion of Cu and Zn sites. (a) HADDF-STEM image of a single slice of CuZn-E-d-MOF particle, (b-e) high-resolution EDS elemental mapping images of (b) Cu, (c) Ni, (d) overlap, and (e) enlarged image of the highlighted pixels in (d) for a single slice of CuZn-E-d-MOF particle.


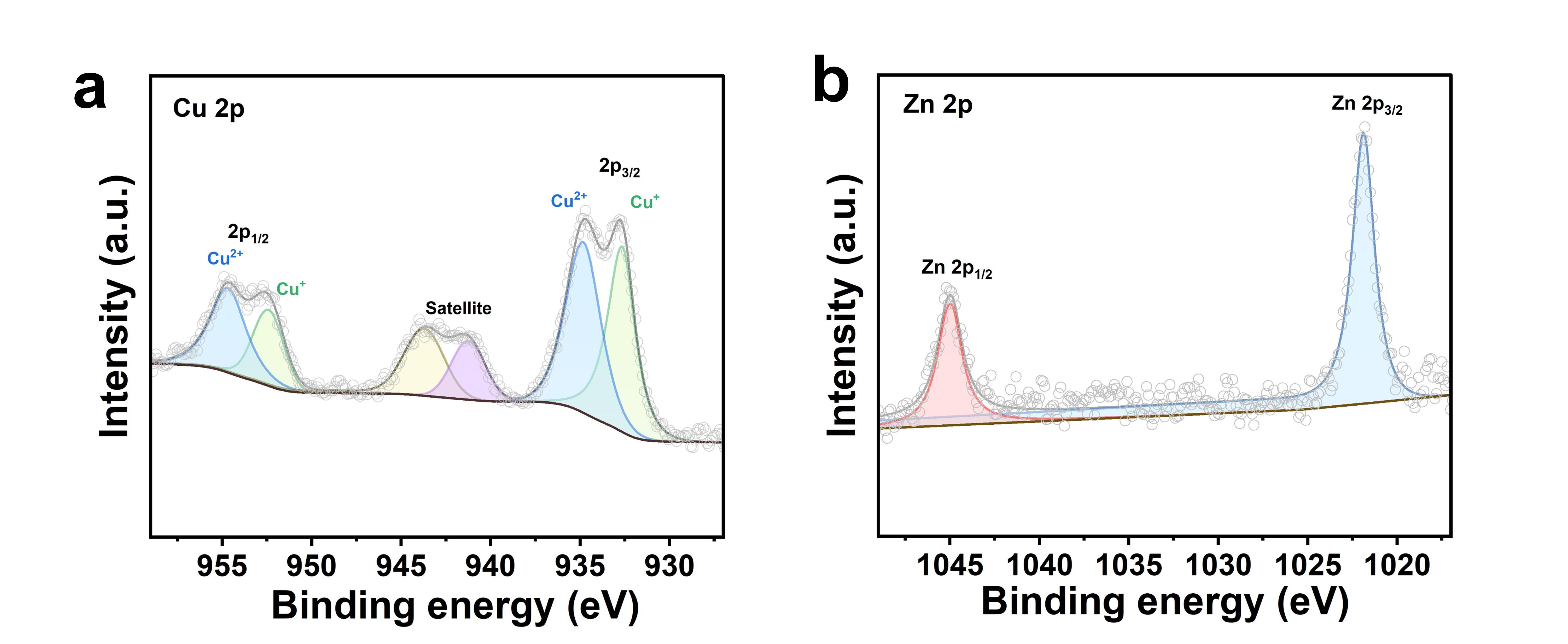


**Figure S12:** XPS spectra. a, b, XPS spectra of (a) Cu 2p and (b) Zn 2p in CuZn-E-d-MOF.


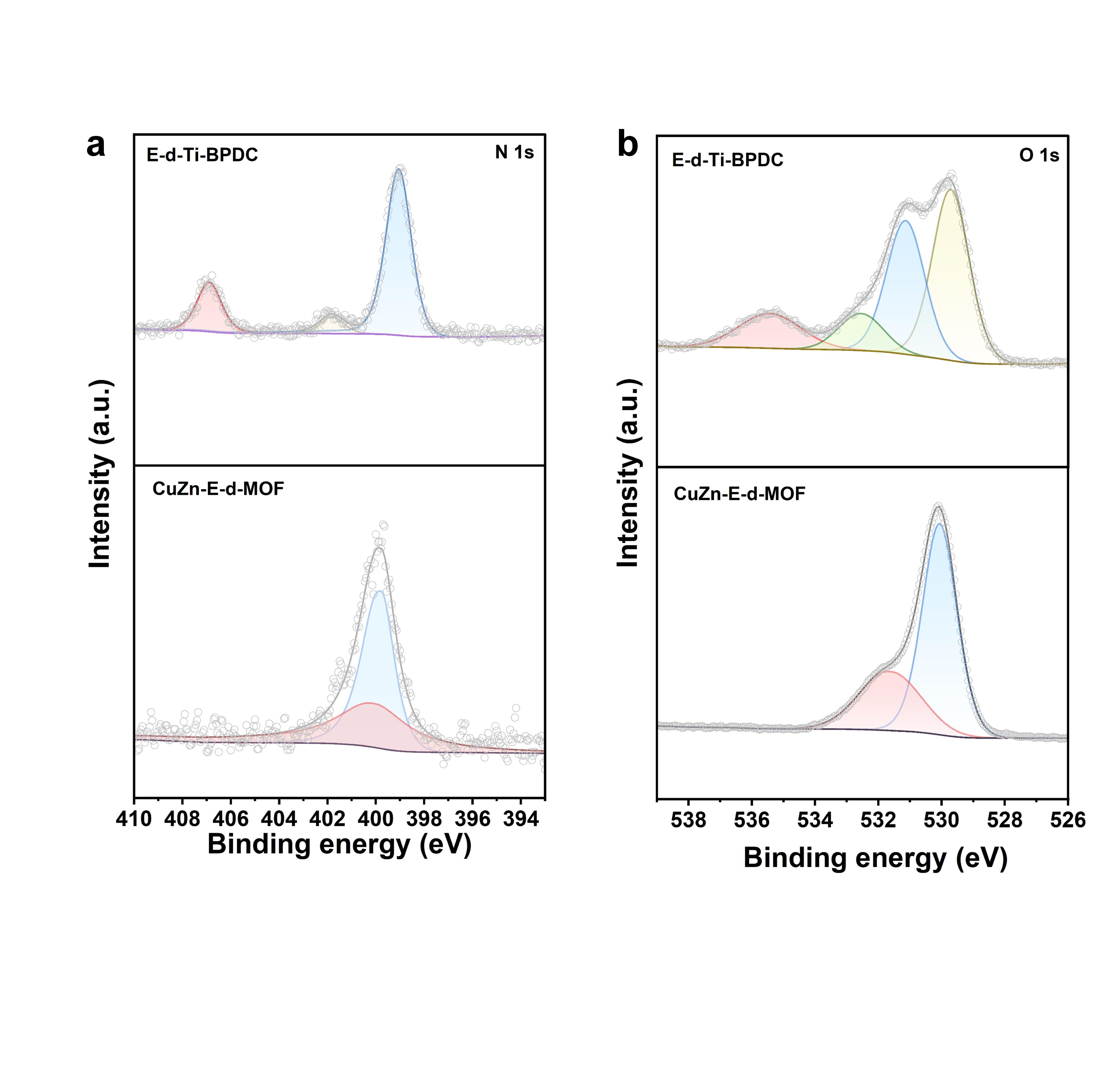


**Figure S13:** XPS spectra. XPS spectra of (a) N 1s and (b) O 1s in E-d-Ti-BPDC and CuZn-E-d-MOF.


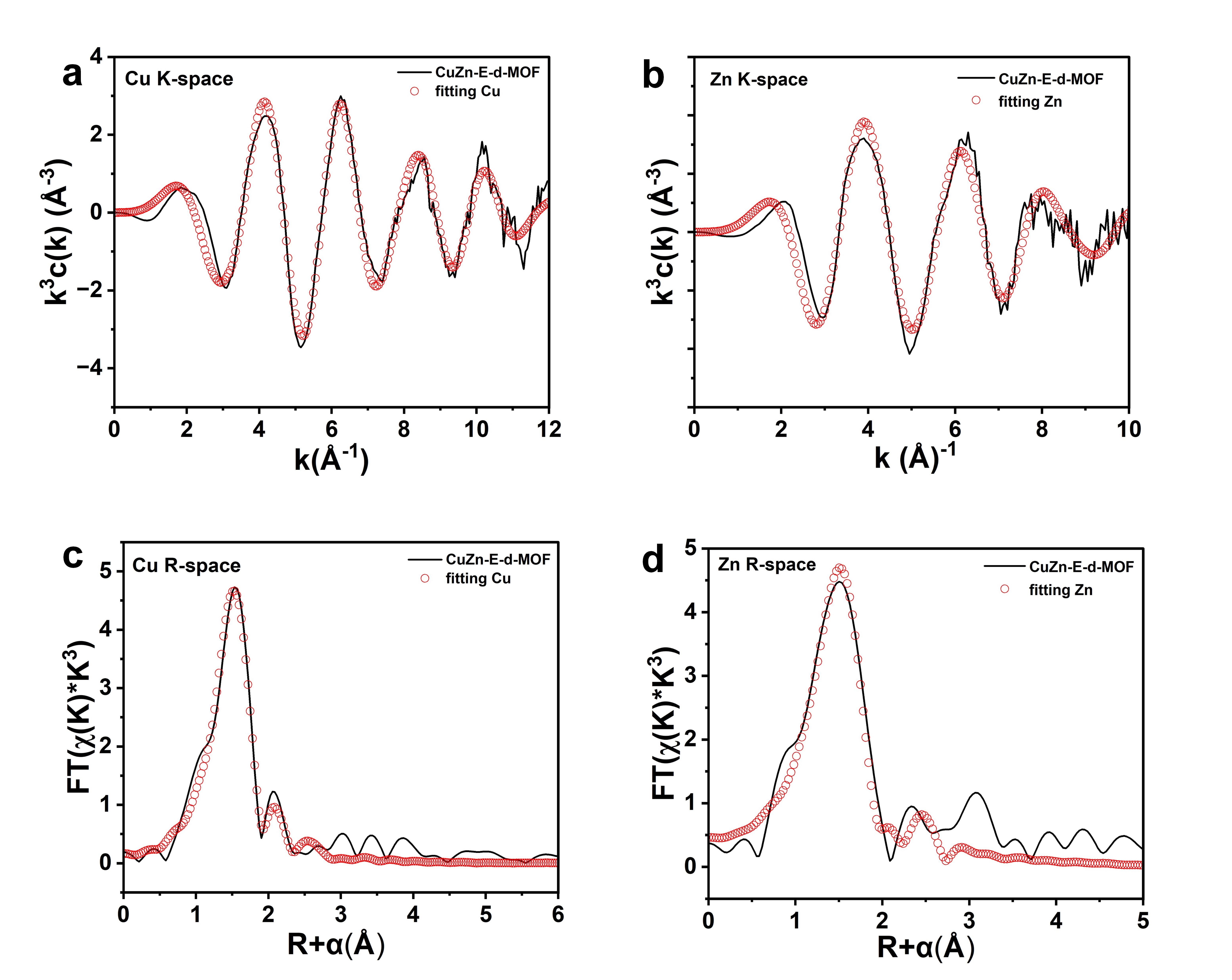


**Figure S14:** The EXAFS fitting curves. a, b, The EXAFS fitting curves at k space of (a) Cu sites and (b) Zn sites. c, d, The EXAFS fitting curves at R space of (c) Cu sites and (d) Zn sites.


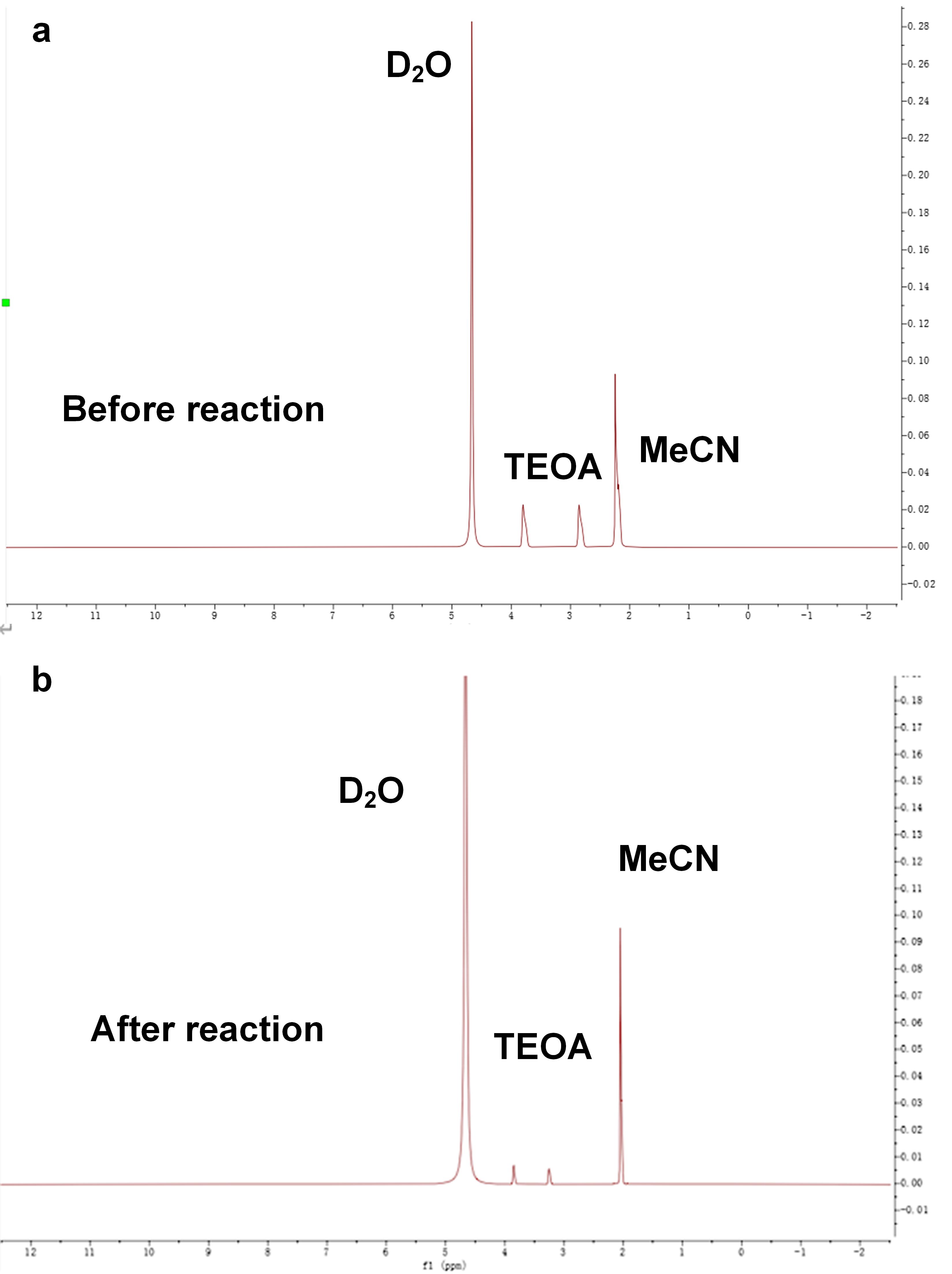


**Figure S15:** ^1^H NMR spectra for the liquid mixture of CH_3_CN and TEOA containing CuZn-E-d-MOF using D_2_O as the deuterated solvent a) before photocatalytic CO_2_ reduction; b) after photocatalytic CO_2_ reduction.


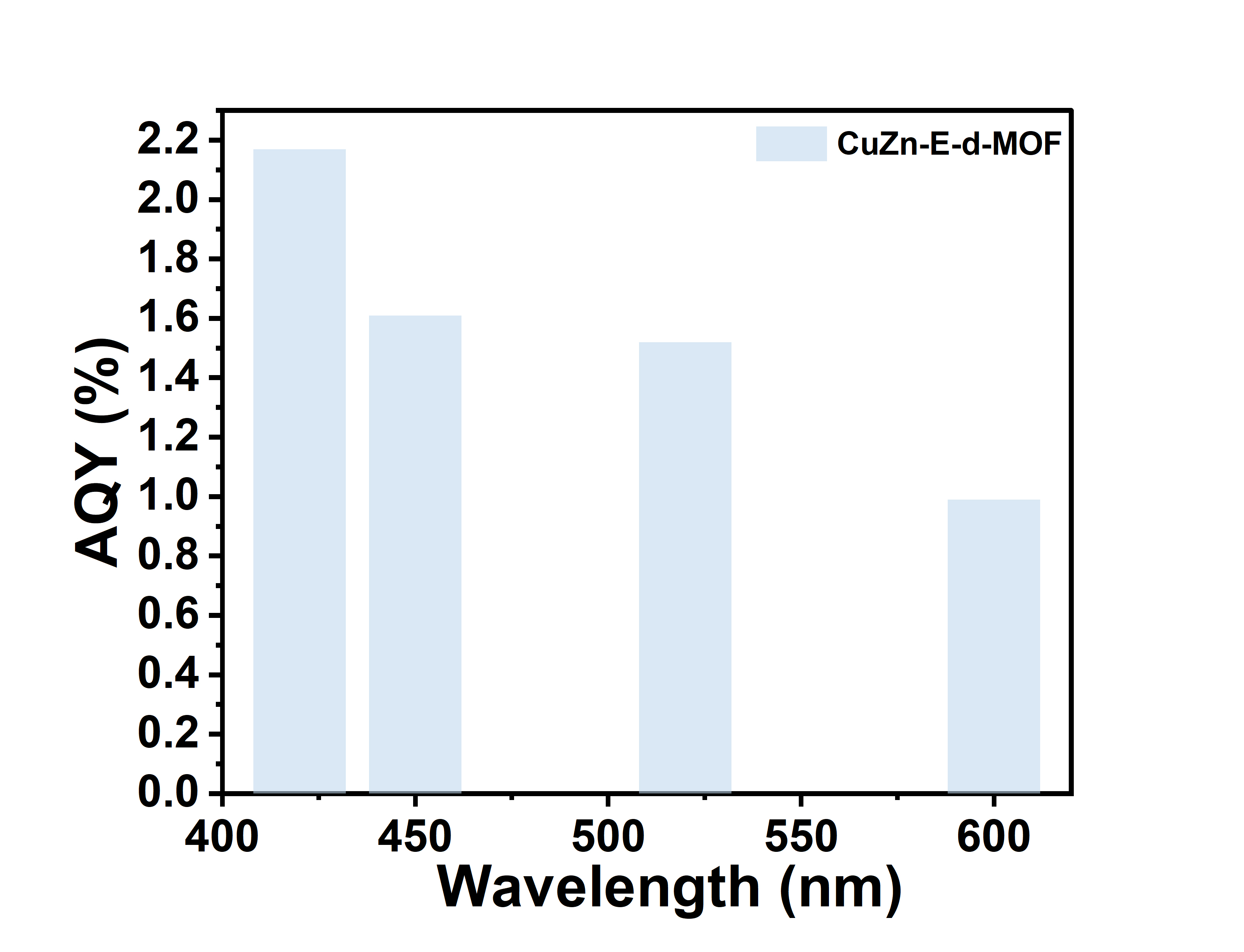


**Figure S16:** Wavelength dependence of AQY during photocatalytic CO_2_ reduction on CuZn-E-d-MOF under the irradiation of 420, 450, 520 and 600 nm wavelength light.


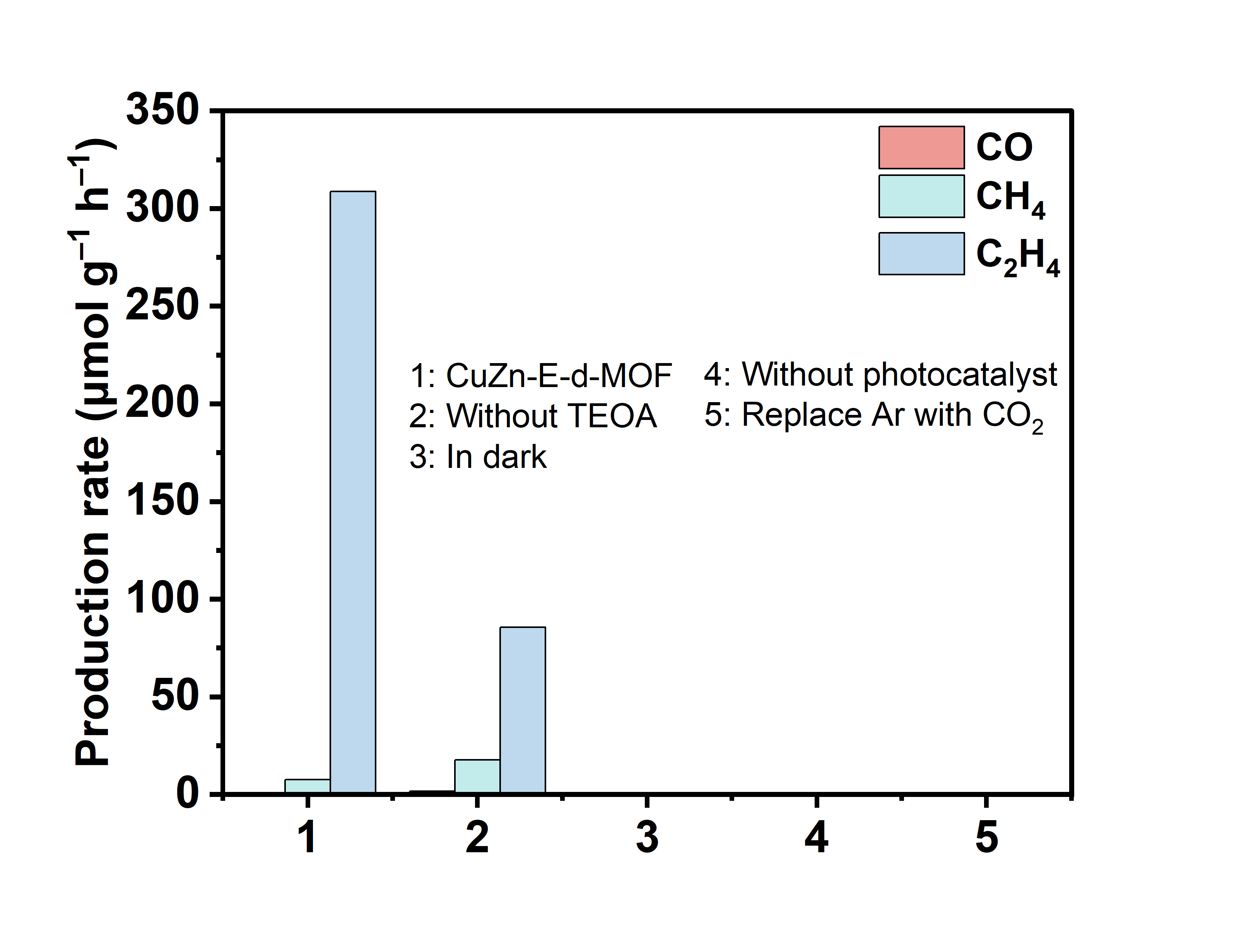


**Figure S17:** The control experiments of photocatalytic CO_2_ reduction performance over CuZn-E-d-MOF under altered conditions.


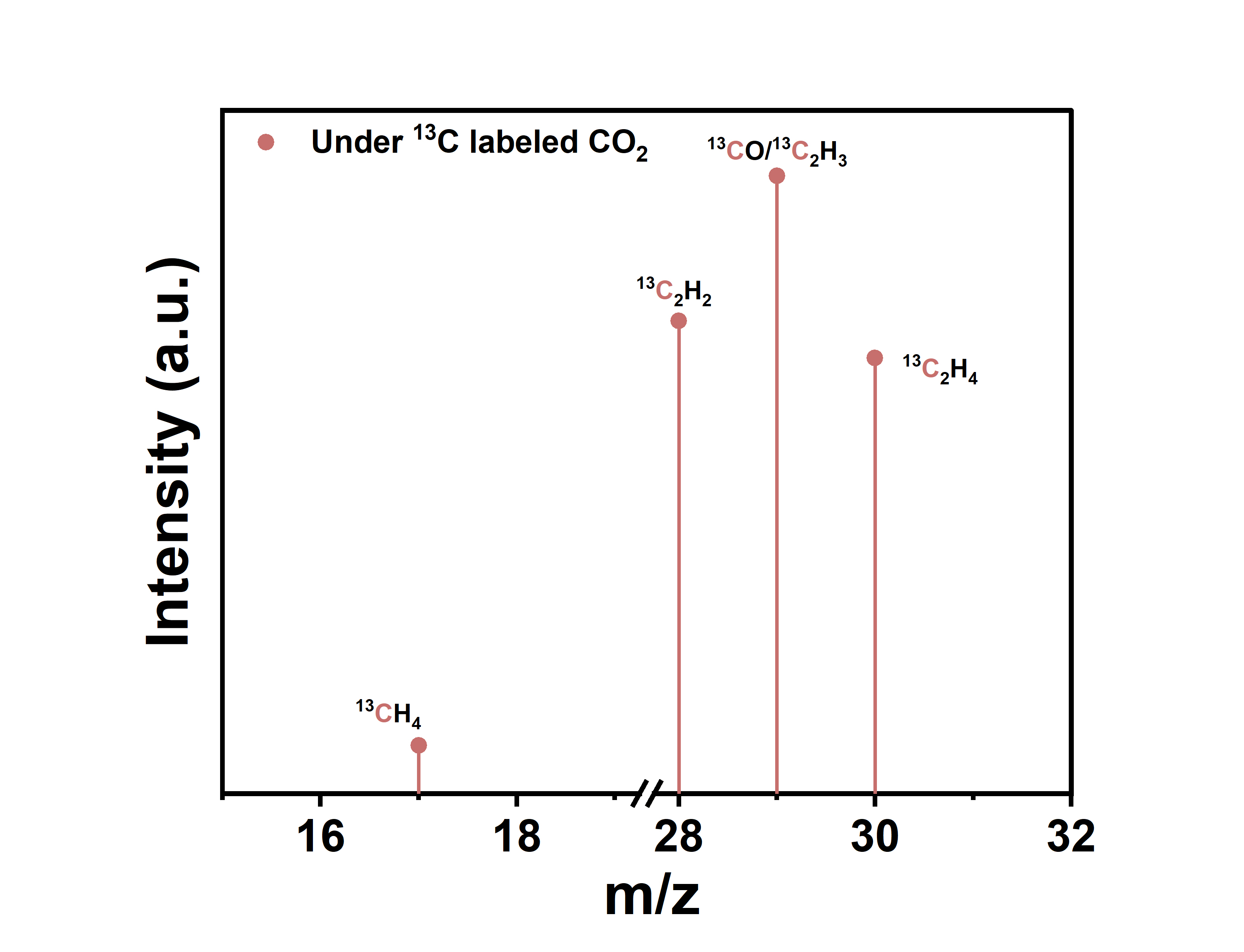


**Figure S18.** GC-MS Spectra for Photocatalytic Reduction of ^13^CO_2_ on CuZn-E-d-MOF


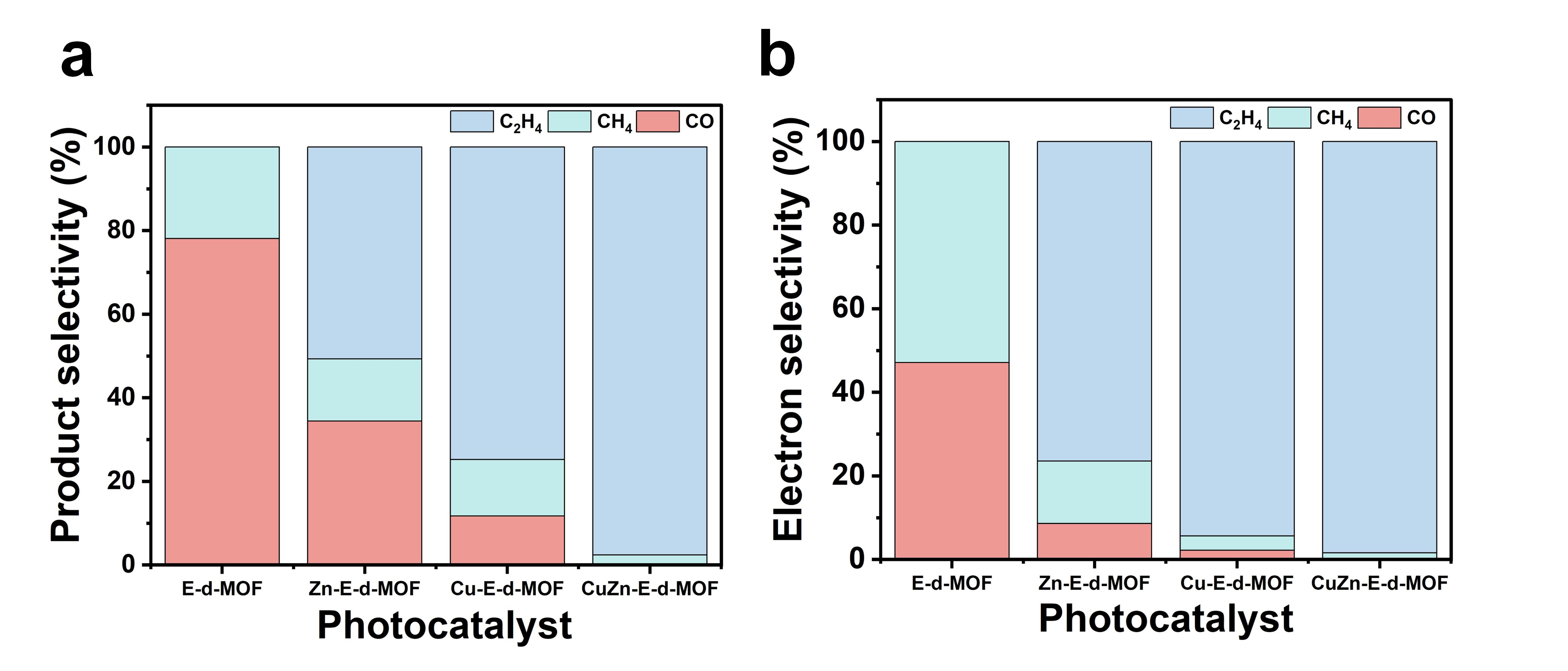


**Figure S19:** The ethane product selectivity **a** and electron selectivity **b** on E-d-MOF, Zn-E-d-MOF, Cu-E-d-MOF and CuZn-E-d-MOF.


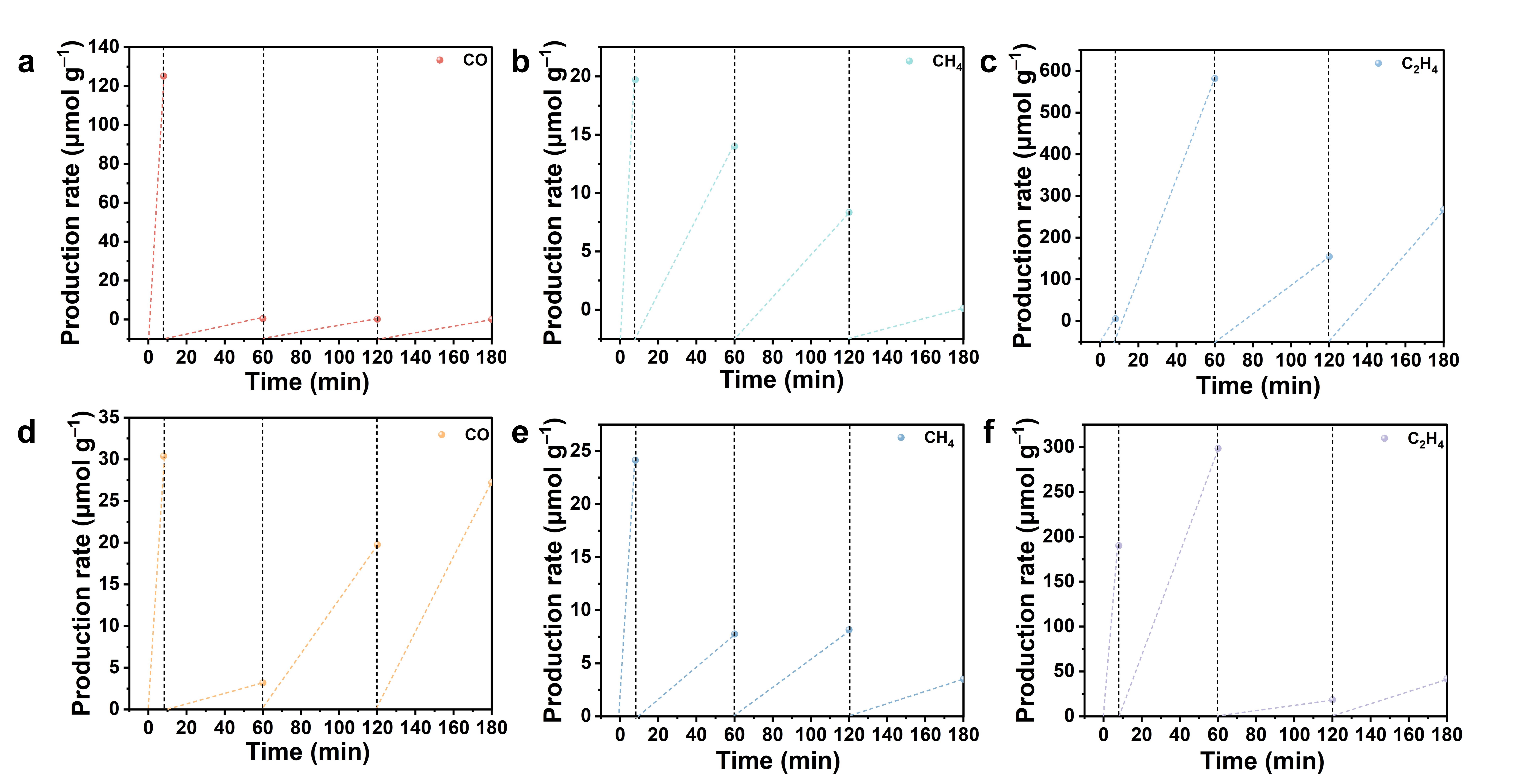


**Figure S20:** CuZn-E-d-MOF photocatalytic CO_2_ generation gas monitoring a CO, b CH_4_, d C_2_H_4_. Cu-E-d-MOF photocatalytic CO_2_ generation gas monitoring d CO, e CH_4_, f C_2_H_4_.


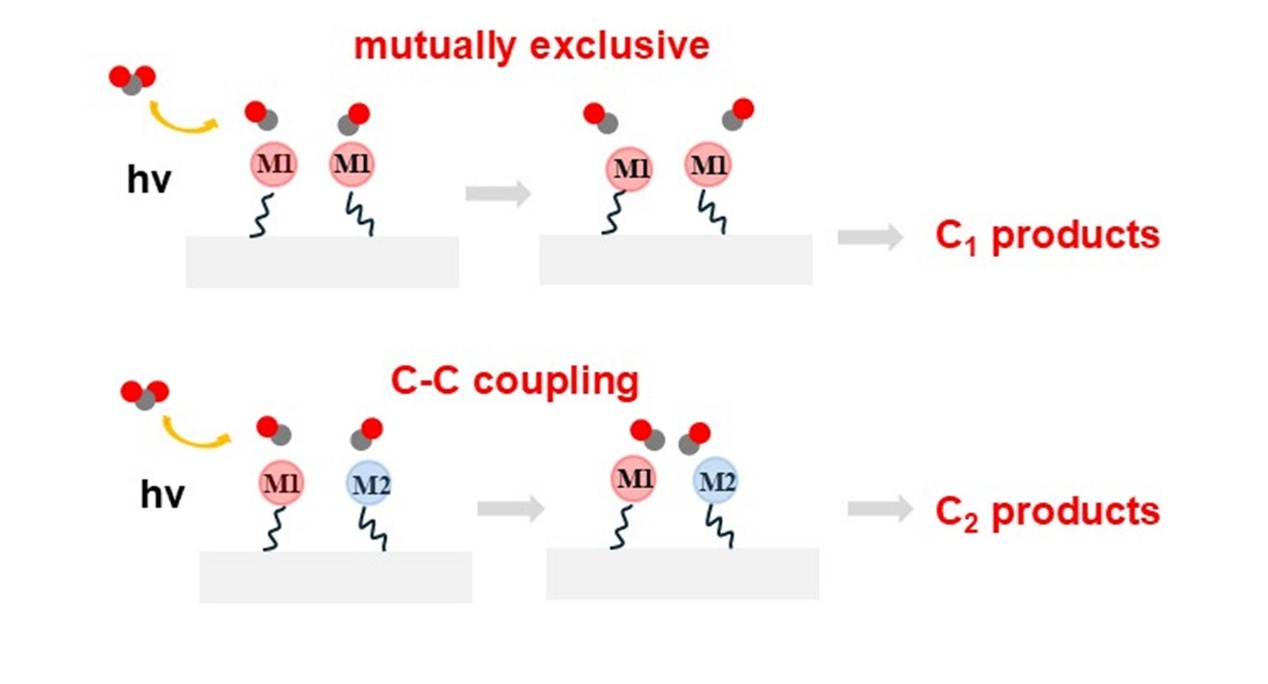


**Figure S21：**Possible CO_2_ Photoreduction Process over Charge Balanced Active Sites and Charge Polarized Active Sites,in which the M Represents the Active Site.


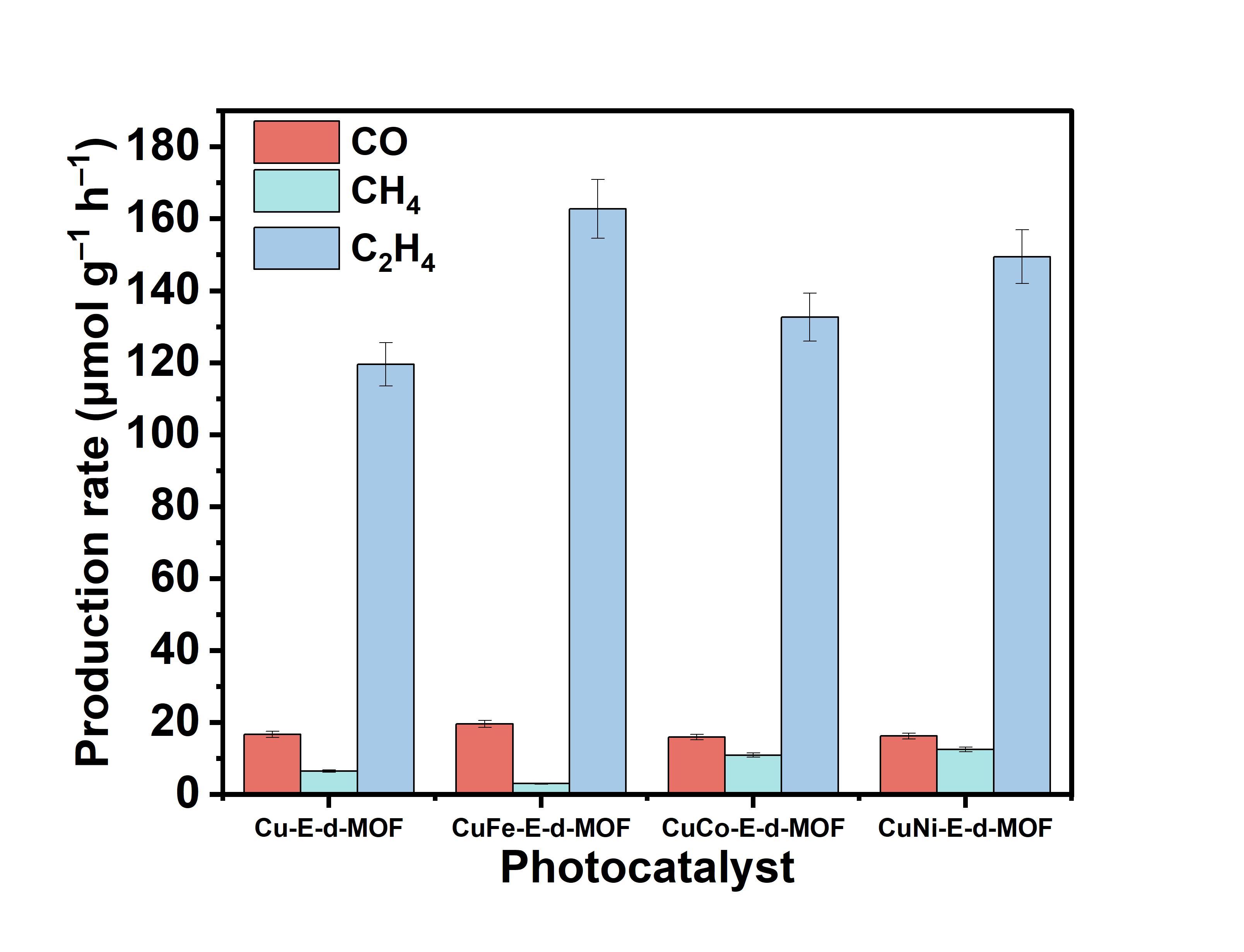


**Figure S22**：Catalytic properties of CuFe-E-d-MOF, CuCo-E-d-MOF, CuNi-d-MOF.


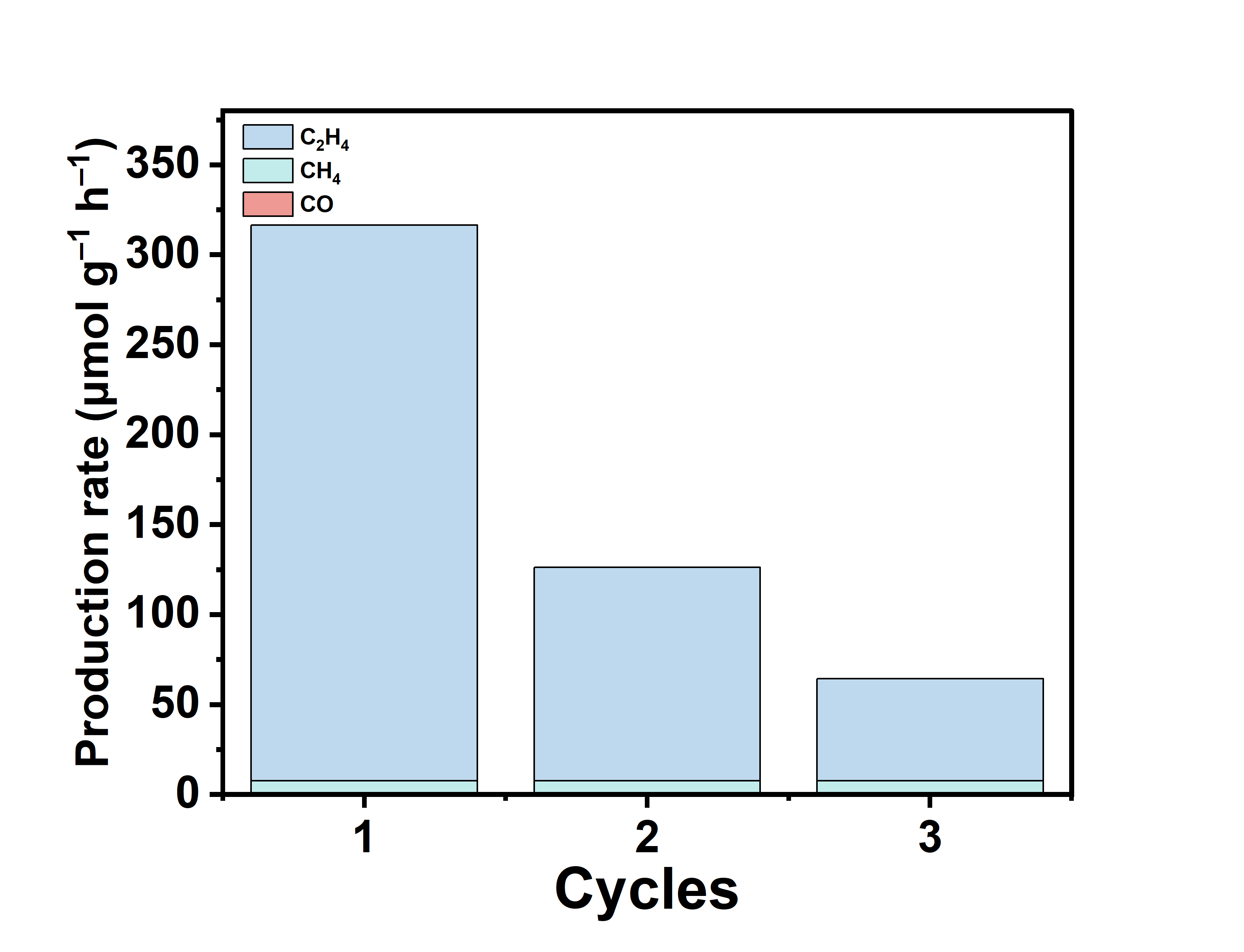


**Figure S23：**Photocatalytic cycling experiment of CuZn-E-d-MOF after the photoreaction without exposure to air.


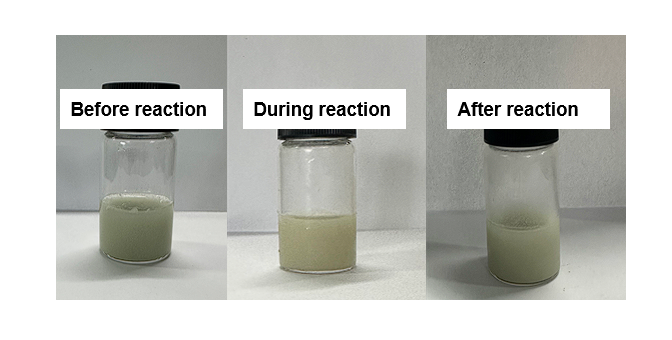


**Figure S24：**The color change of CuZn-E-d-MOF before, during and after reaction


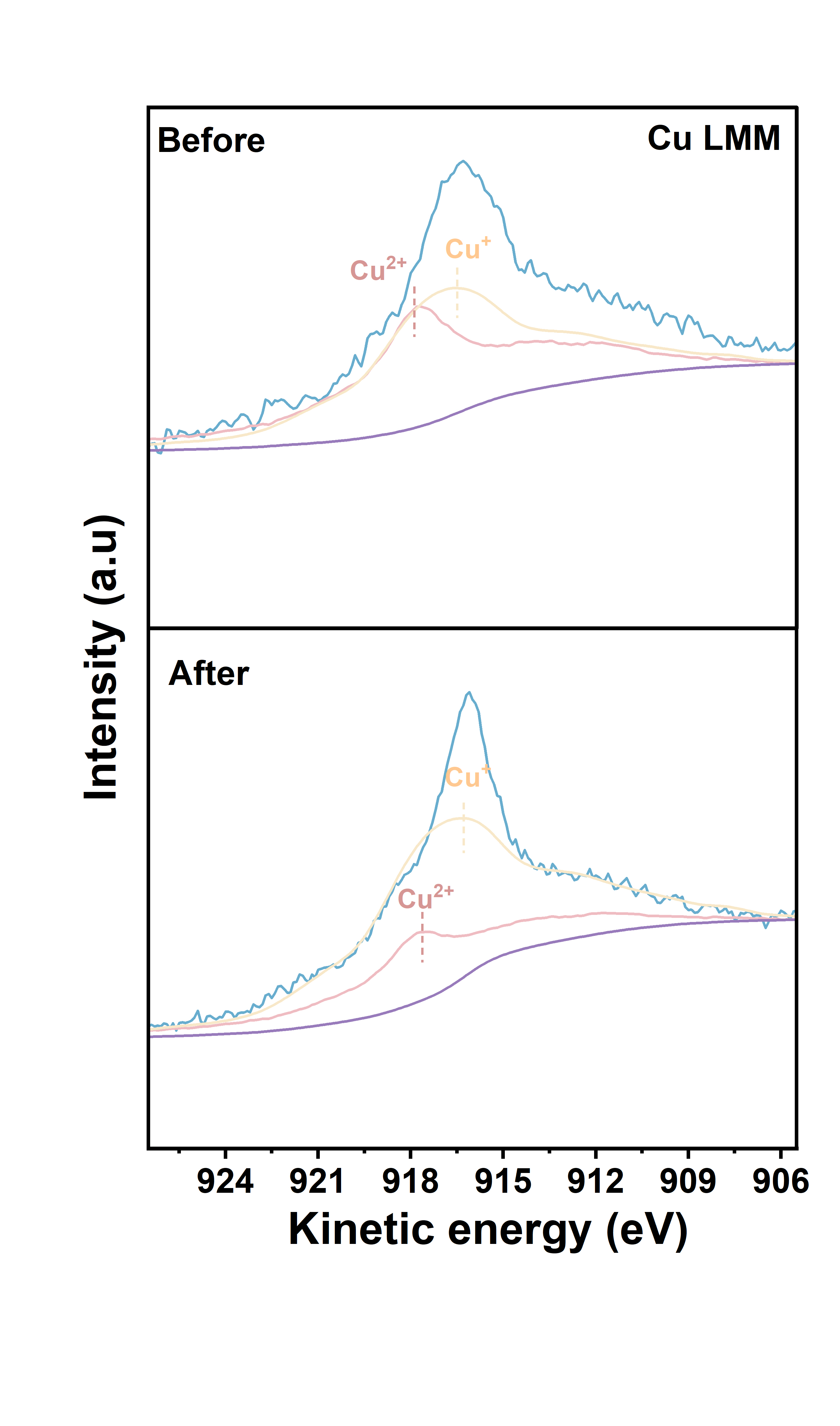


**Figure S25：**Cu LMM Auger spectra for CuZn-E-d-MOF before and after photocatalysis.


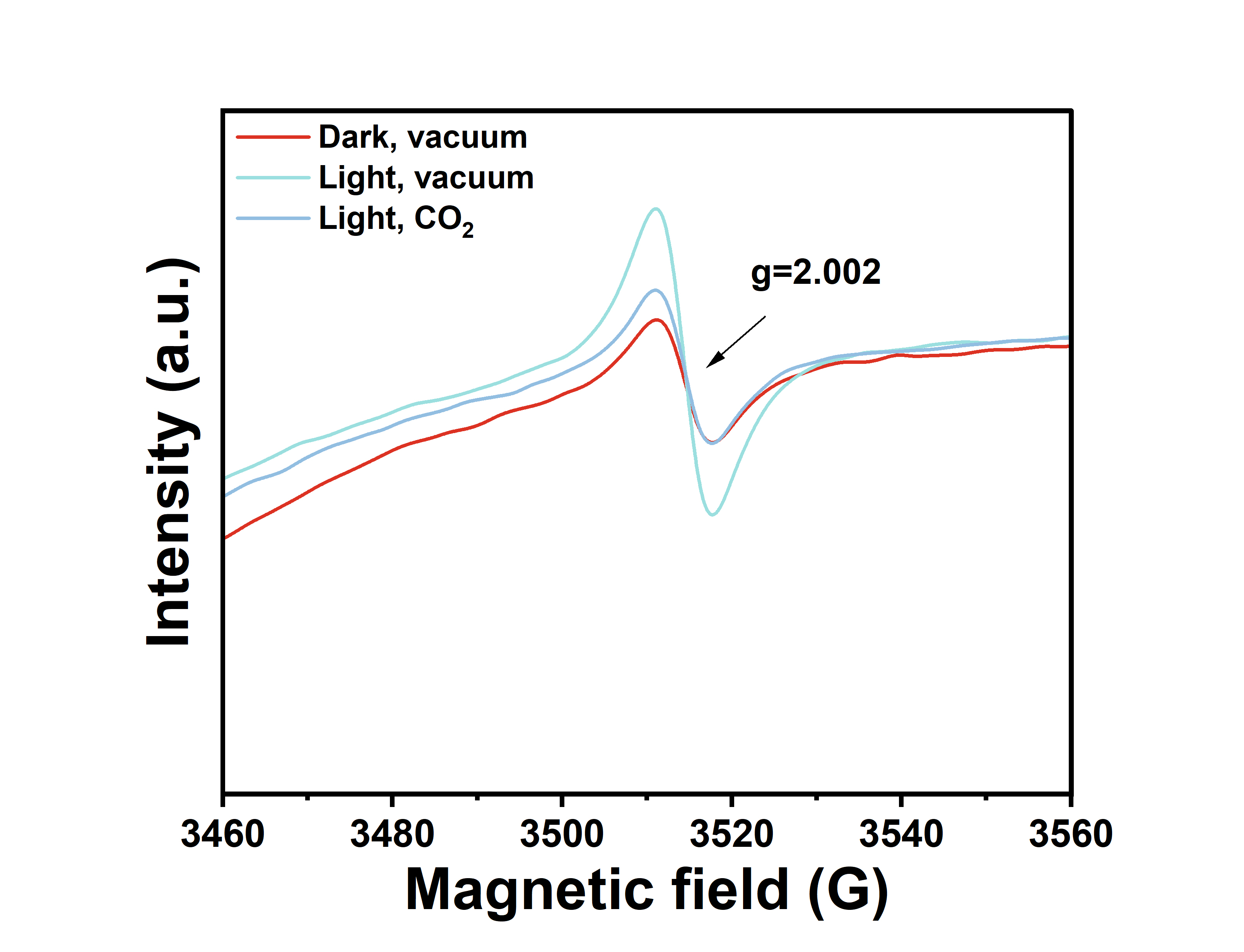


**Figure S26：**Quasi in-situ EPR spectra of CuZn-E-d-MOF under different conditions.


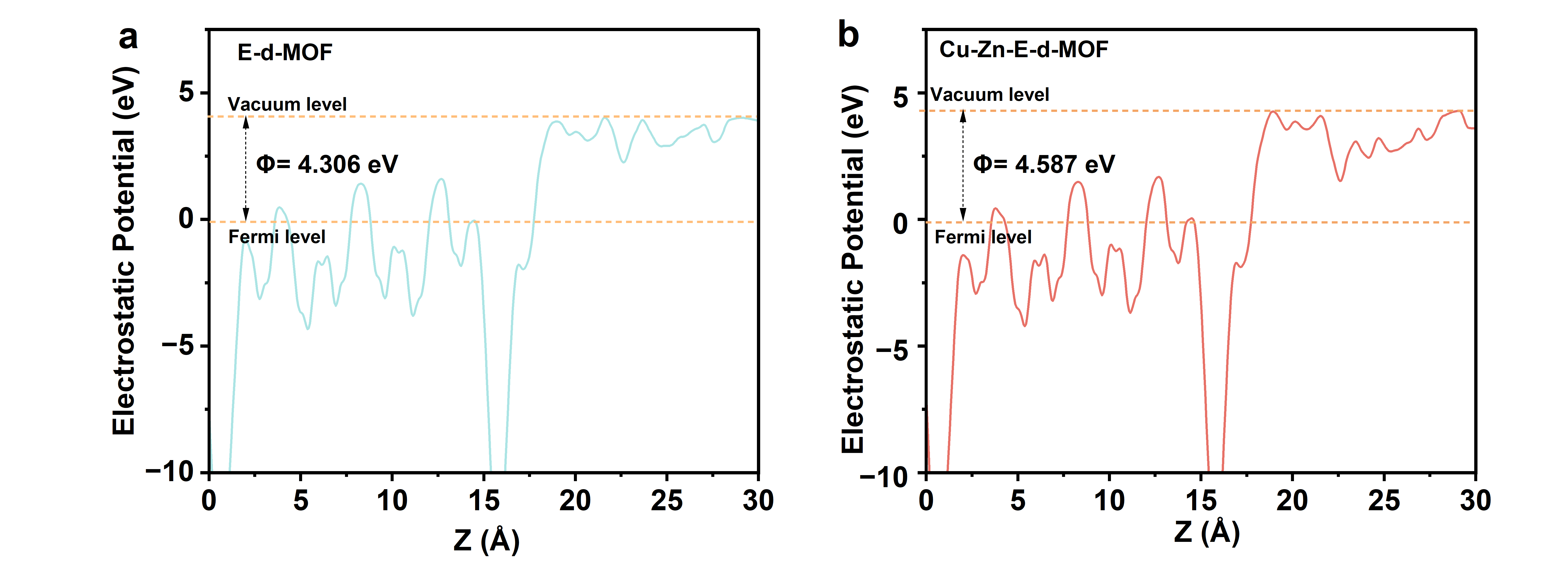


**Figure S27：**Calculated electrostatic potentials of E-d-MOF and CuZn-E-d-MOF.


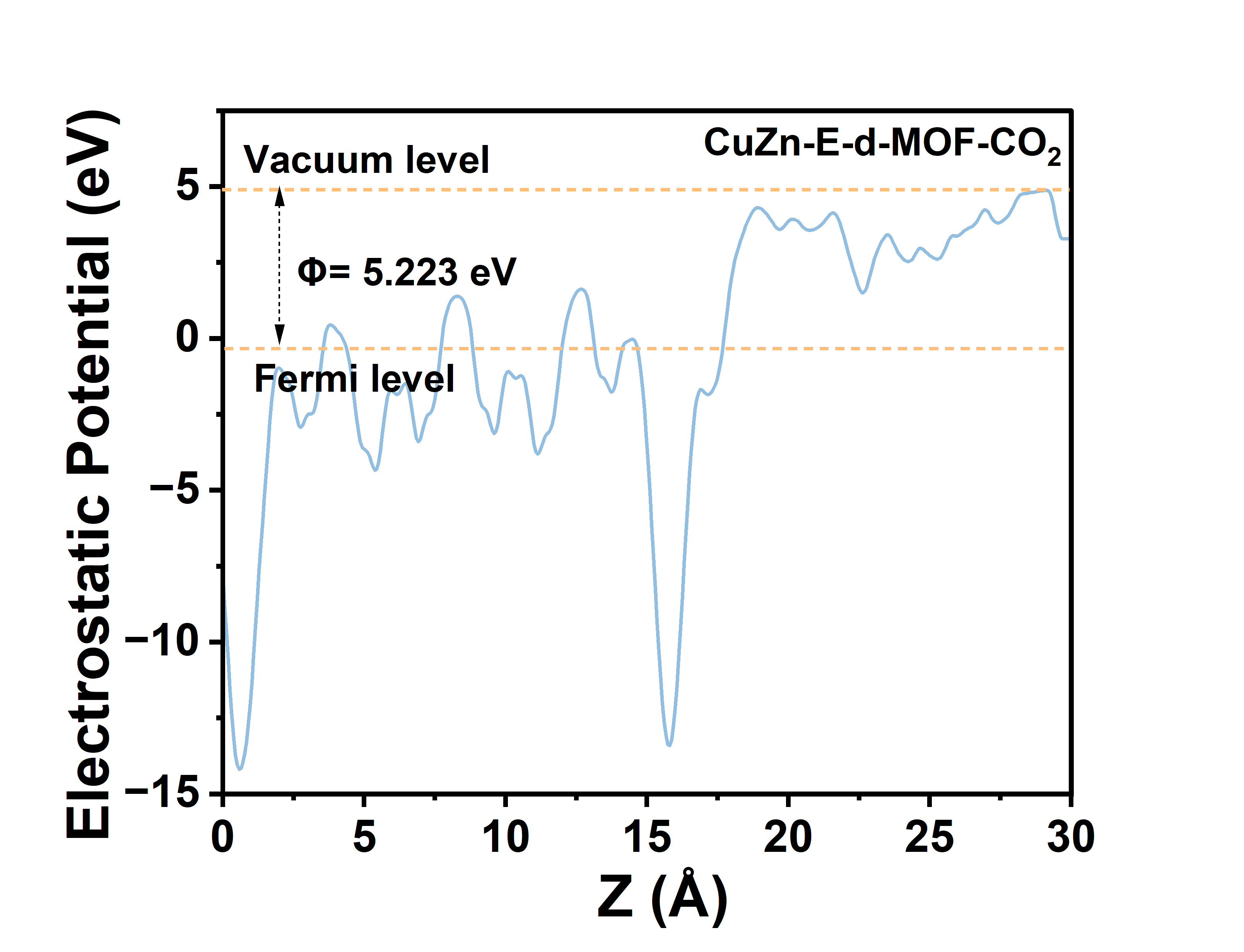


**Figure S28：**Calculated electrostatic potentials of the system with CuZn-E-d-MOF with CO_2_.


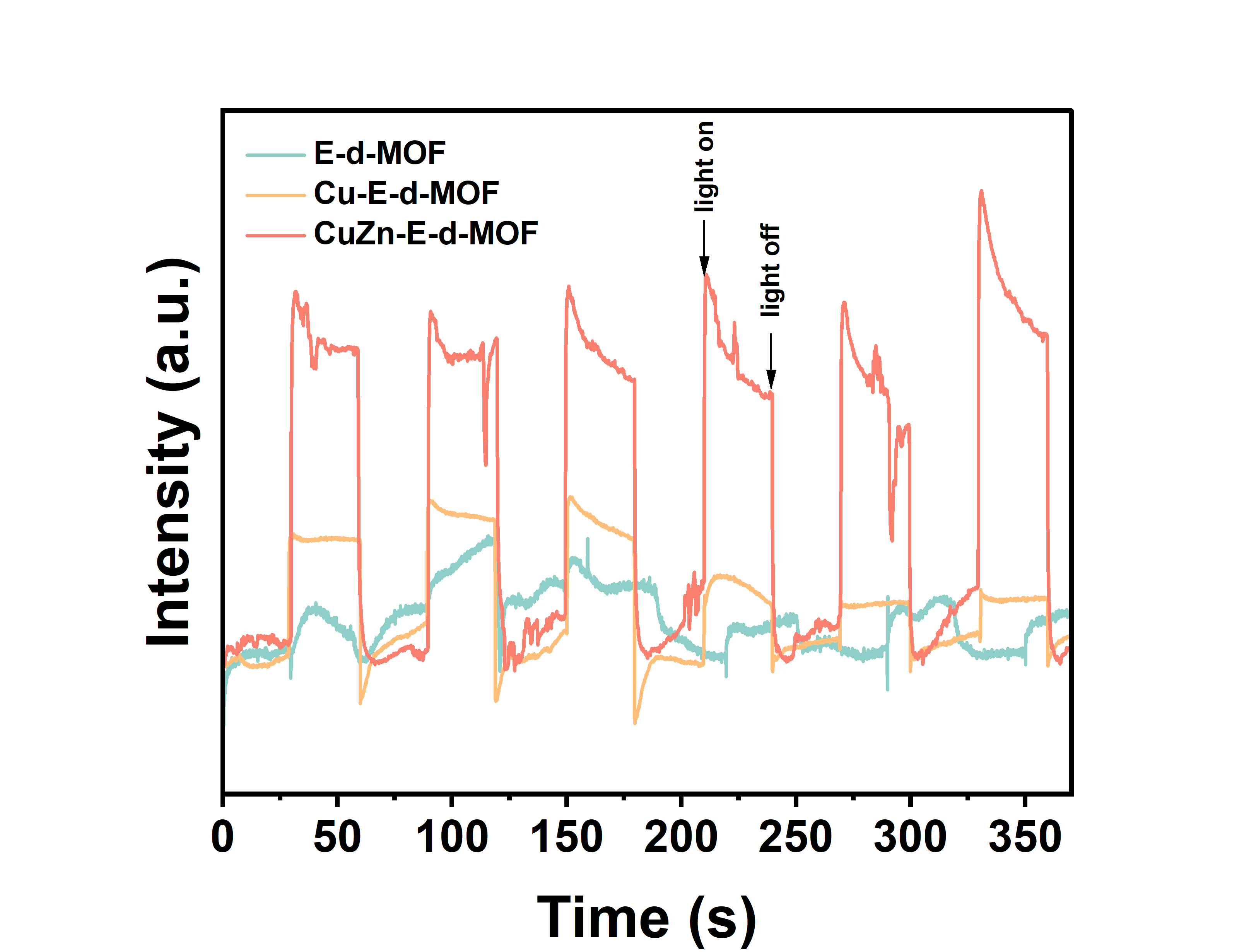


**Figure S29：**Transient photocurrent response.


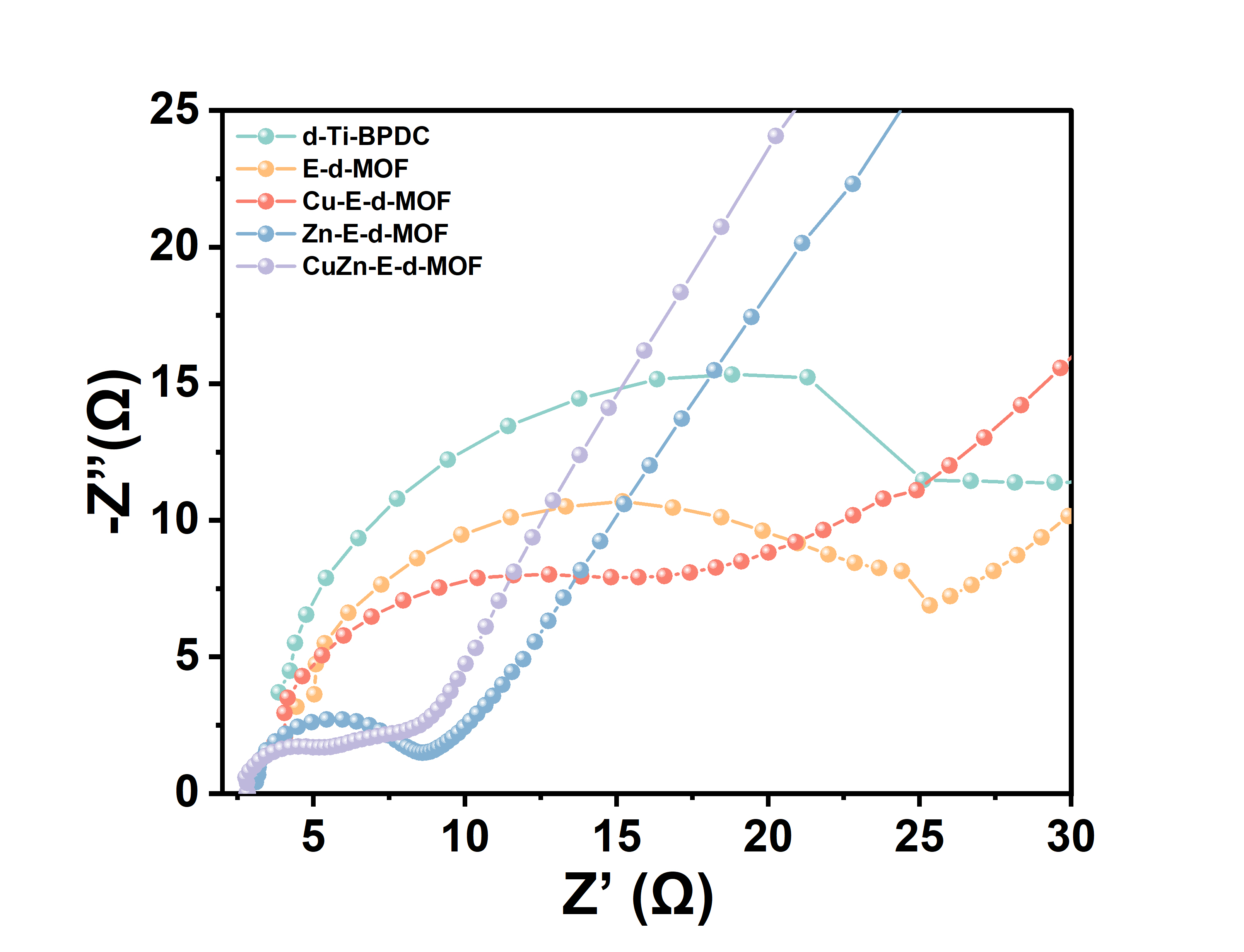


**Figure S30：**EIS Nyquist plots.


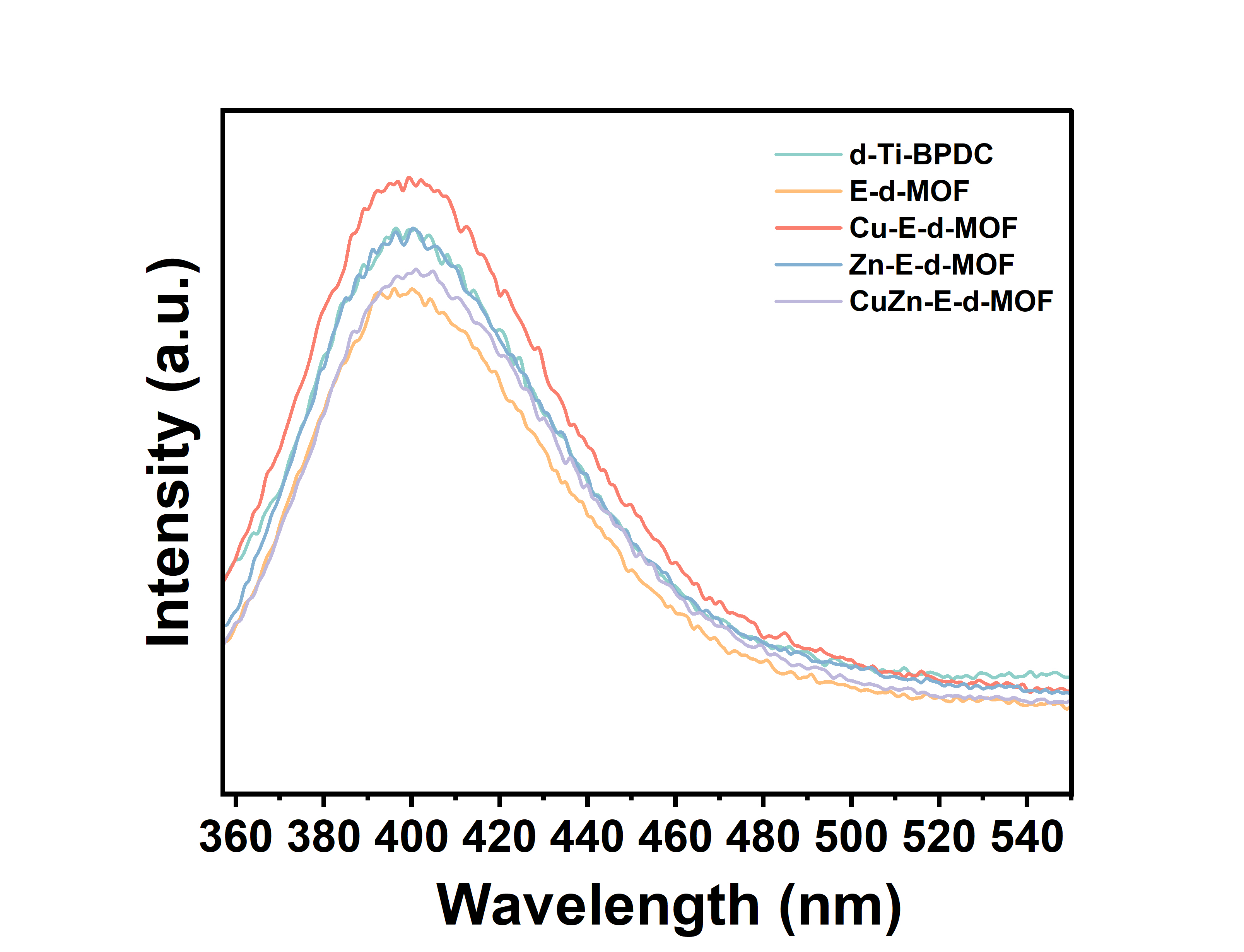


**Figure S31：**photoluminescence spectra of d-Ti-BPDC, E-d-MOF, Cu-E-d-MOF, Zn-E-d-MOF and CuZn-E-d-MOF.


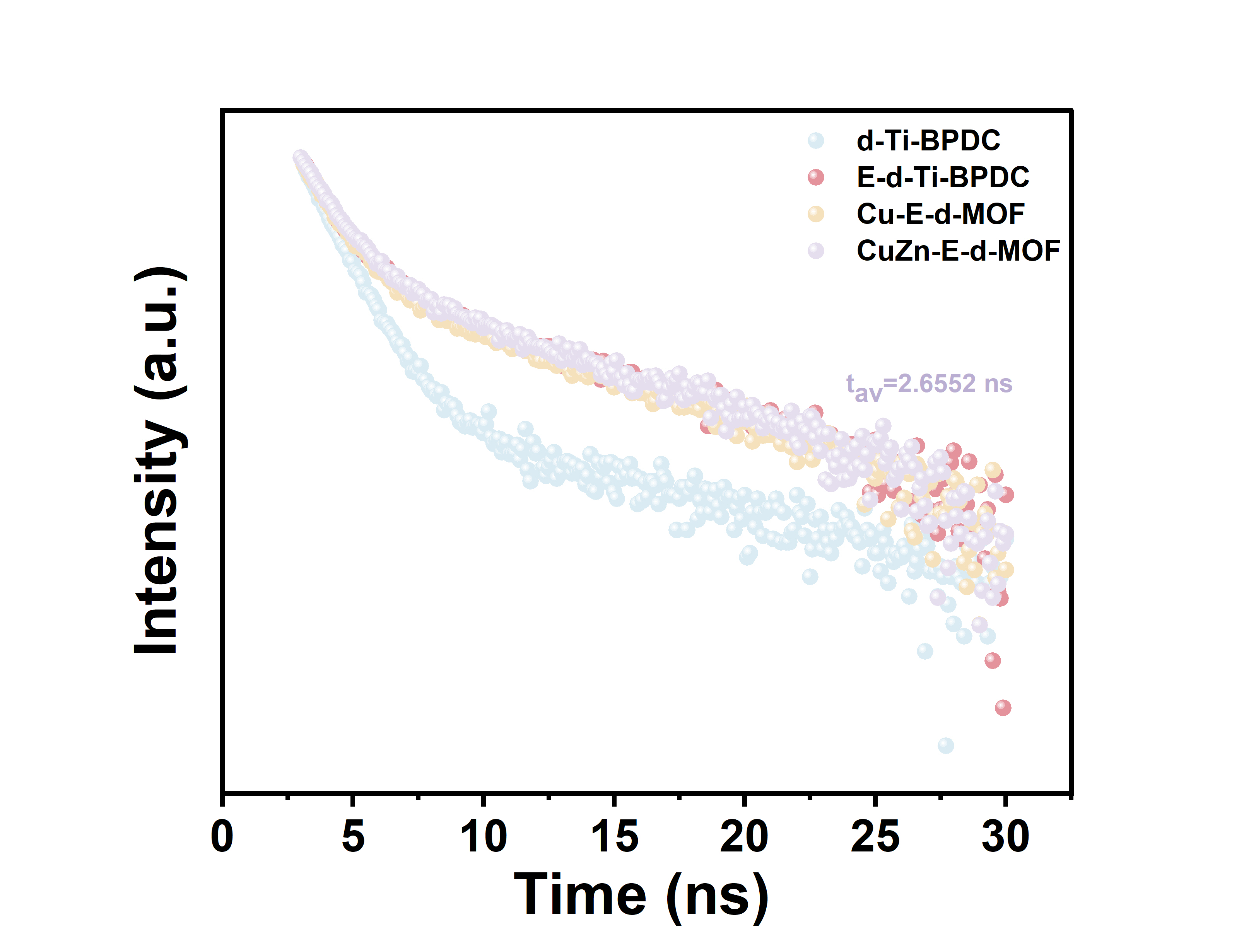


**Figure S32：**TRPL decay spectra of d-Ti-BPDC, E-d-MOF, Cu-E-d-MOF and CuZn-E-d-MOF.


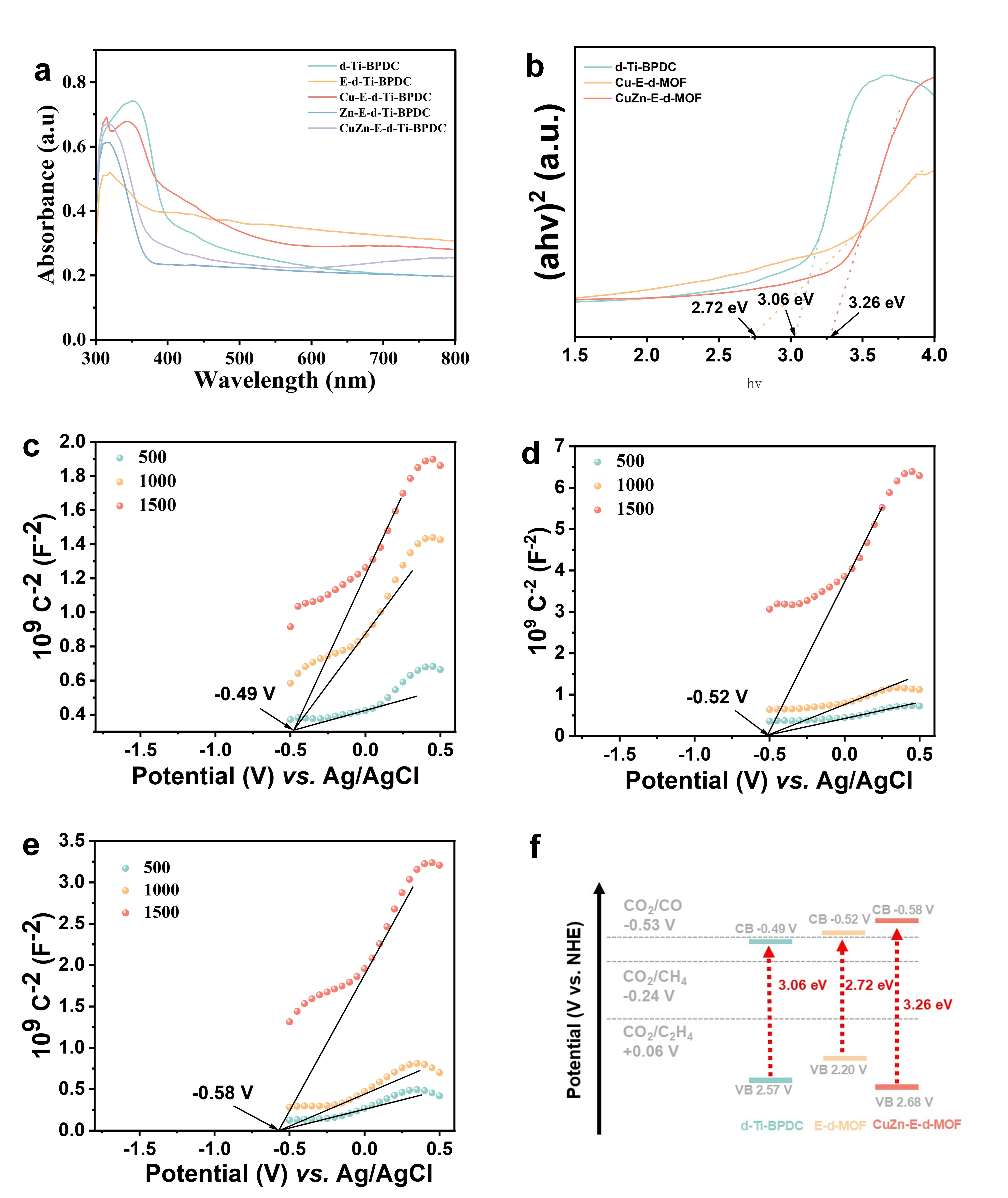


**Figure S33：**(a) UV-vis diffuse reflectance spectra (DRS), (b) obtained bandgaps of 3.06eV, 2.72eV and 3.26 eV, estimated by plotting (αhν)^2^ versus hν (Tauc plot). α and ν are the absorbance and wavenumber. (c, d, e) Mott−Schottky plots of d-Ti-BPDC,E-d-MOF, and CuZn-E-d-MOF in 0.5 M Na_2_SO_4_ aqueous solution at 500, 1000, and 1500 Hz. (f) Schematic energy level diagrams of d-Ti-BPDC, E-d-MOF, CuZn-E-d-MOF.


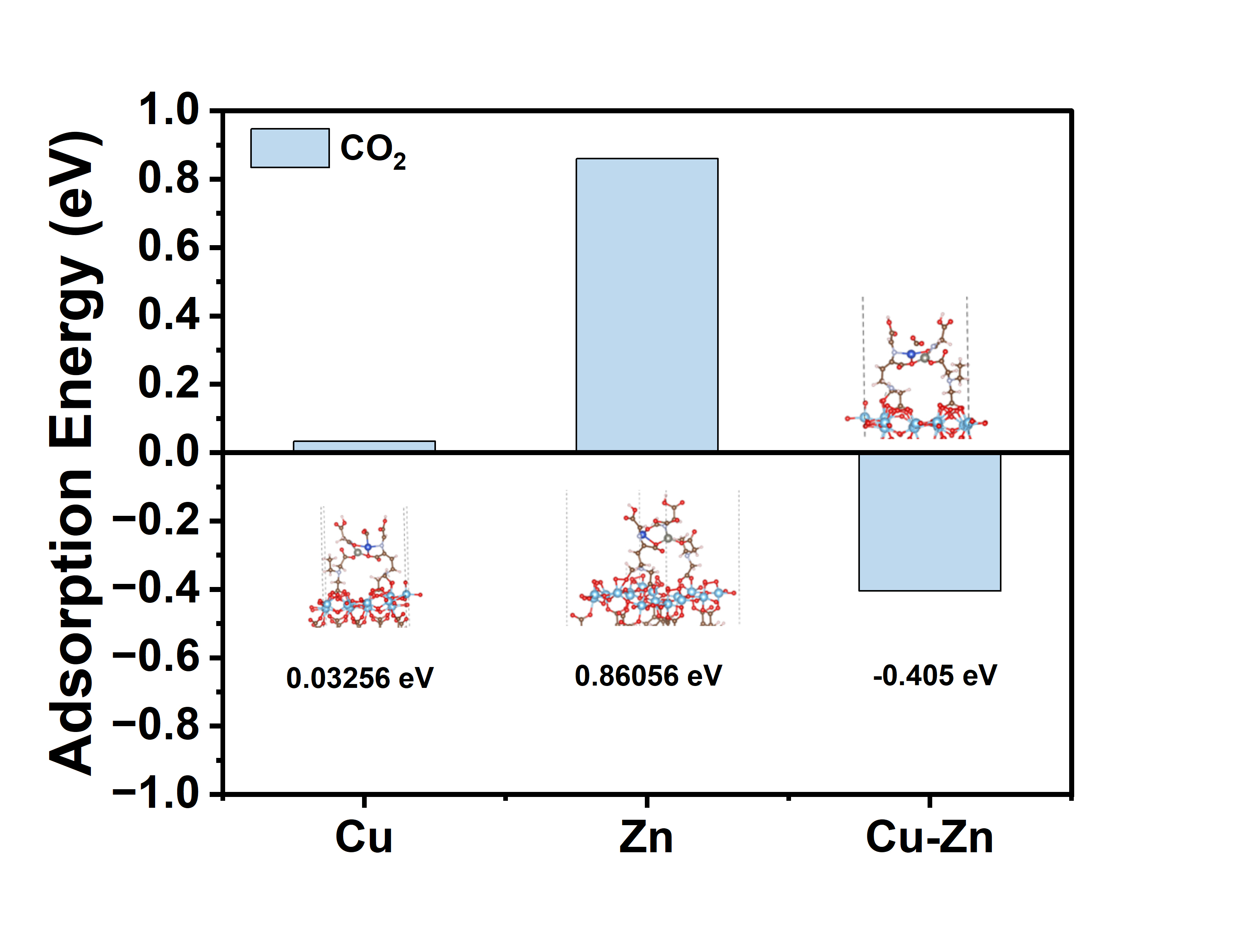


**Figure S34：**Calculated CO_2_ adsorption energies and corresponding structures.


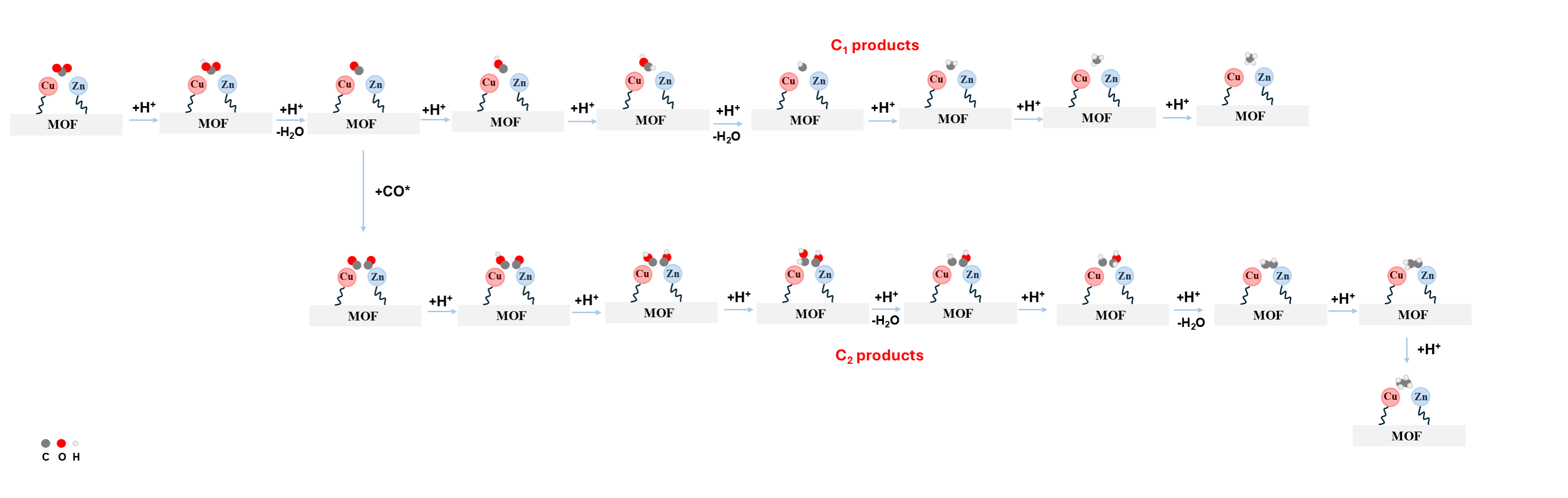


**Supplementary Fig. 35：**Proposed CO_2_RR mechanism over CuZn-E-d-MOF system.


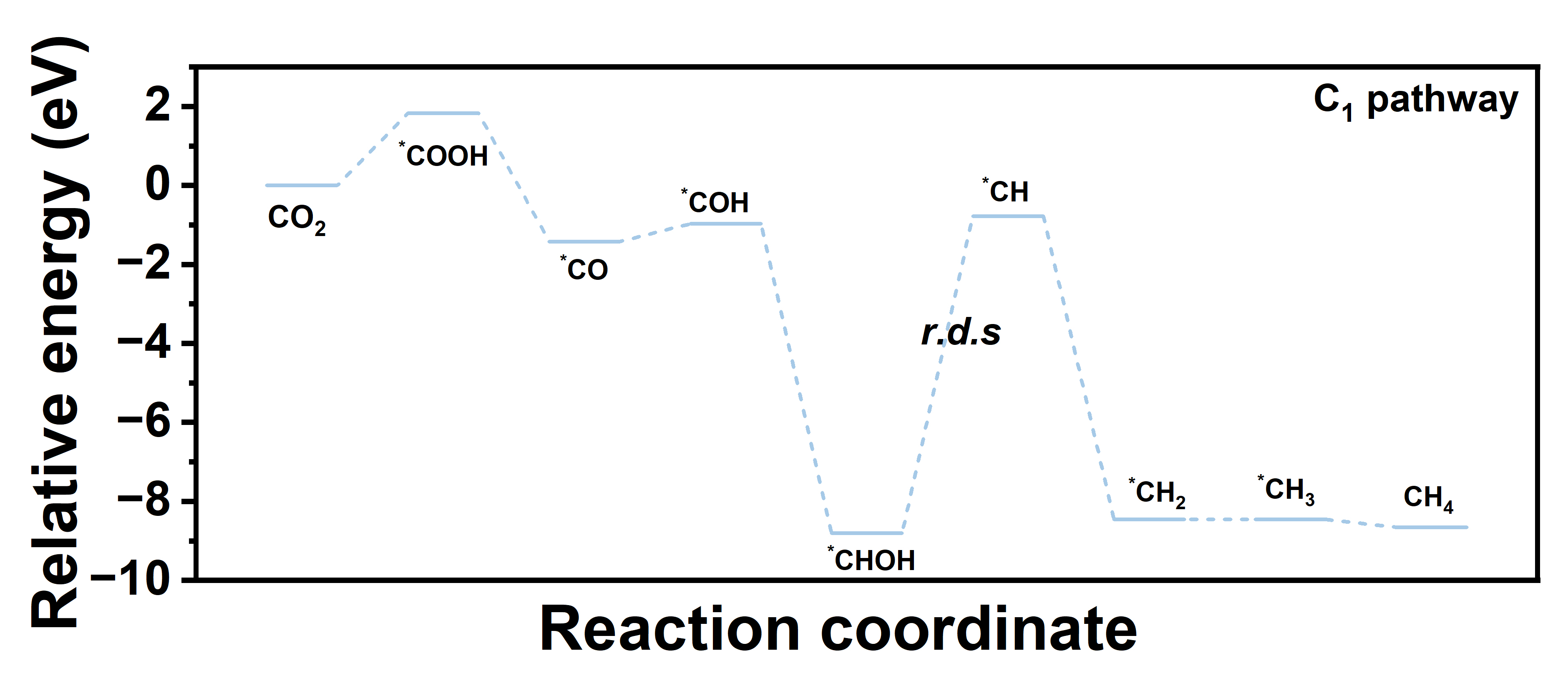


**Figure S36：**Gibbs free energy diagram of CO_2_ reduction to CH_4_ for CuZn-E-d-MOF.


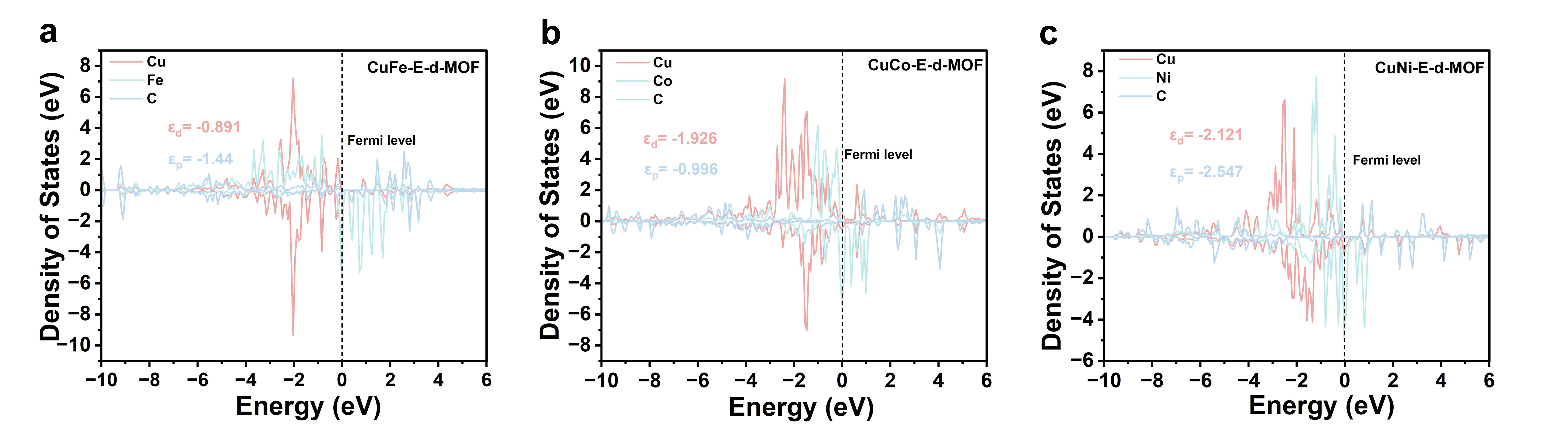


**Figure S37：**Density of state (DOS) calculation for *OCCO adsorbed on the CuFe-E-d-MOF, CuCo-E-d-MOF and CuZn-E-d-MOF.

**Table S1.** The EXAFS fitting parameters. EXAFS fitting parameters at the Zn K-edge and Cu K-edge for different samples (S0^2^ = 1(Ni), 1(Cu))

| **Cu K-edge** | | | | | | |
| --- | --- | --- | --- | --- | --- | --- |
| **Sample** | **Path** | **N^a^** | **R/Å^b^** | **σ^2^/10^-3^ Å^2c^** | **ΔE/eV** | **R-factor** |
| CuZn-E-d-MOF | Cu-O | 3.6±0.39 | 1.96 | 8.5 | -1.10 | 0.0079 |
| Cu foil | Cu-Cu | 12 | 2.54 | 9.74 | -0.01234 | 0.01479 |
| Cu_2_O | Cu-O | 1.736 | 1.85 | 4.13 | 0.014 | 0.010 |
|  | Cu-Cu | 8 | 2.99 | 10.7 | 0.014 | 0.10 |
| CuO | Cu-O | 2.51 | 1.95 | 2.42 | -0.747 | 0.0096 |
|  | Cu-Cu | 4 | 3.27 | 8.97 | -0.747 | 0.0096 |
|  | Cu-O | 8 | 3.52 | 10.24 | -0.747 | 0.0096 |
| **Zn K-edge** | | | | | | |
| CuZn-E-d-MOF | Zn-O | 3.91±0.47 | 1.9838 | 3.0 | -1.64 | 0.01 |
| Zn foil | Zn-Zn | 4 | 2.64 | 13.32 | 0.01 | 0.004 |
| ZnO | Zn-O | 2.91 | 1.9654 | 3.46 | -0.040 | 0.003 |
|  | Zn-Zn | 12 | 3.24 | 11.6 | -0.035 | 0.003 |

**^a^N:** coordination numbers; **^b^R:** bond distance;**^c^σ^2^:** Debye-Waller factors; **ΔE_0_:** the inner potential correction. **R factor:** goodness of fit. Ѕ0^2^ was set to 1 for Zn and 1 for Cu, according to the experimental EXAFS fit of Zn and Cu foil by fixing CN as the known crystallographic value.

**Table S2.** Cu metal and Zn metal loadings of CuZn-E-d-MOF.

| Sample name | Zn Loadings (%) | Cu Loadings (%) |
| --- | --- | --- |
| CuZn-E-d-MOF | 1.2594 | 11.42859922 |

**Table S3.** Comparison of Quantum Yields Across Different Materials

| Photocatalyst | Wavelength (nm) | AQY (%) | Reference |
| --- | --- | --- | --- |
| CuZn-E-d-MOF | 420 | ~2.15 | This work |
| CIS-Cu_1.5_ | 420 | ~1.02 | [6] |
| CCS-H1 | 500 | ~1.54 | [7] |
| Culn-ABPS | 400 | ~4.3 | [8] |
| CuGaS_2_ | 620 | ~0.75 | [9] |

**Table S4.** The solution obtained after the photocatalytic reaction with CuZn-E-d-MOF.

| Element | Concentration (ug/ml) | Dissolution rate (%) |
| --- | --- | --- |
| Cu | 0.285974151 | 0.294 |
| Zn | 0.023604769 | 0.197 |

**Table S5.** The efficiency and selectivity of plasmonic photocatalysts for CO_2_ RR and the references.

| **Photocatalysts** | **Rection conditon** | | **C_2_H_4_ rate (μmol g^-1^ h^-1^)** | | | **Selectivity (%)** | | **References** |
| --- | --- | --- | --- | --- | --- | --- | --- | --- |
| CuZn-E-d-MOF | | MeCN/TEOA/H_2_O | | 308.72 | | 98.4 | This work | |
| Ultra-thin CGS/GS | | H_2_O/TEOA | | 335 | | 93.87 | [10] | |
| CuInP_2_S_6_ | | H_2_O | | 20.89 | | 70.6 | [11] | |
| CuACs/PCN | | H_2_O/TEOA/C_30_H_24_C_l2_N_6_Ru-6H_2_O | | 10.2 | | 53.2 | [12] | |
| Cu_1_/W_18_O_49_ (3.6-Cu_1_/W_18_O_49_) | | MeCN/TEOA | | 4.9 | | 72.8 | [13] | |
| CuGaS_2_@CuO | | H_2_O | | 20.6 | | 92.7 | [9] | |
| Re-bpy/PTF(Cu) | | H_2_O/TEOA | | 73.2 | | 21.4 | [14] | |
| Cu_2_MoS_4_ | | H_2_O | | 35.3 | | 34 | [15] | |
| Bi_19_S_27_Br_3_-VS | | H_2_O | | 17.8 | | 85 | [16] | |
| Cu_2.63_P/g-C_3_N_4_ | | C_2_H_5_OH/H_2_O | | | 3.58 | 64.25 | [17] | |

**Table S6.** Multiexponential fitting parameters for the decay plots of TRPL lifetime.

|  | A_1_ (%) | τ_1_ (ns） | A_2_ (%) | τ_2_ (ns） | τ_av_ (ns） |
| --- | --- | --- | --- | --- | --- |
| d-Ti-BPDC | 11.26762 | 1.2132 | 0.04487 | 8.631 | 1.41756 |
| E-d-MOF | 9.12577 | 1.241 | 0.27746 | 8.1755 | 2.398178 |
| Cu-E-d-MOF | 8.77588 | 1.2668 | 0.23287 | 8.7489 | 2.425608 |
| CuZn-E-d-MOF | 8.20678 | 1.3053 | 0.26259 | 8.8664 | 2.655242 |

The average lifetime (τ_av_) can be calculated according to a dual-exponential function: τav = (A_1_τ_1_^2^ + A_2_τ_2_^2^)/( A_1_τ_1_ + A_2_τ_2_). Where A_1_ and A_2_ and are the pre-exponential factors, τ_1_ and τ_2_ represent the lifetime (ns) of the process of radiation and non-radiation energy transfer, respectively.

**Table S7.** The optimization structure of CuZn-E-d-MOF

1.0

12.5109996796 0.0000000000 0.0000000000

-4.4858940348 13.7467868139 0.0000000000

0.0000000000 0.0000000000 34.5500984192

C O Ti N H Cu Zn

116 106 32 20 76 1 1

Direct

| 0.307600000 | 0.080960000 | 0.147480000 |
| --- | --- | --- |
| 0.403990000 | 0.081860000 | 0.208110000 |
| 0.999720000 | 0.083970000 | 0.153540000 |
| 0.407020000 | 0.083140000 | 0.127750000 |
| 0.306270000 | 0.081250000 | 0.187460000 |
| 0.322410000 | 0.102850000 | 0.312010000 |
| 0.403950000 | 0.079880000 | 0.250980000 |
| 0.404920000 | 0.072260000 | 0.330620000 |
| 0.321500000 | 0.106730000 | 0.271920000 |
| 0.153440000 | 0.321150000 | 0.393310000 |
| 0.158420000 | 0.331480000 | 0.104880000 |
| 0.484380000 | 0.048130000 | 0.307360000 |
| 0.051190000 | 0.318010000 | 0.330690000 |
| 0.149020000 | 0.328620000 | 0.270000000 |
| 0.246340000 | 0.331850000 | 0.326800000 |
| 0.151380000 | 0.323970000 | 0.349800000 |
| 0.049590000 | 0.321780000 | 0.290490000 |
| 0.070040000 | 0.360530000 | 0.166460000 |
| 0.149970000 | 0.330950000 | 0.227000000 |
| 0.153000000 | 0.331230000 | 0.147010000 |
| 0.068410000 | 0.360460000 | 0.206480000 |
| 0.411300000 | 0.082530000 | 0.086060000 |
| 0.408240000 | 0.069270000 | 0.373820000 |
| 0.231660000 | 0.304170000 | 0.170000000 |
| 0.807040000 | 0.079810000 | 0.149640000 |
| 0.902080000 | 0.080480000 | 0.210700000 |
| 0.500670000 | 0.084870000 | 0.150980000 |
| 0.906700000 | 0.082380000 | 0.129920000 |
| 0.804820000 | 0.079710000 | 0.189650000 |
| 0.816120000 | 0.100670000 | 0.314480000 |
| 0.901010000 | 0.078890000 | 0.253760000 |
| 0.899400000 | 0.072900000 | 0.333960000 |
| 0.816560000 | 0.103940000 | 0.274300000 |
| 0.670720000 | 0.321510000 | 0.388530000 |
| 0.658750000 | 0.331340000 | 0.101820000 |
| 0.980840000 | 0.050490000 | 0.311030000 |
| 0.561600000 | 0.321490000 | 0.327260000 |
| 0.657050000 | 0.332550000 | 0.266190000 |
| 0.757070000 | 0.335280000 | 0.322160000 |
| 0.662670000 | 0.325990000 | 0.345520000 |
| 0.558630000 | 0.326090000 | 0.287150000 |
| 0.572780000 | 0.361760000 | 0.163350000 |
| 0.655720000 | 0.333670000 | 0.223350000 |
| 0.654850000 | 0.331760000 | 0.143760000 |
| 0.573140000 | 0.362810000 | 0.203320000 |
| 0.911460000 | 0.082080000 | 0.088040000 |
| 0.901340000 | 0.071610000 | 0.377650000 |
| 0.734500000 | 0.304830000 | 0.166250000 |
| 0.306010000 | 0.578350000 | 0.147970000 |
| 0.402130000 | 0.583230000 | 0.208840000 |
| 0.001400000 | 0.584990000 | 0.150490000 |
| 0.406220000 | 0.581400000 | 0.128530000 |
| 0.303930000 | 0.579760000 | 0.187900000 |
| 0.317350000 | 0.609240000 | 0.311940000 |
| 0.401920000 | 0.584600000 | 0.251660000 |
| 0.400940000 | 0.581960000 | 0.331230000 |
| 0.317490000 | 0.610520000 | 0.271820000 |
| 0.158760000 | 0.824710000 | 0.392130000 |
| 0.160570000 | 0.830950000 | 0.105690000 |
| 0.482940000 | 0.559040000 | 0.308710000 |
| 0.058360000 | 0.830350000 | 0.330580000 |
| 0.154140000 | 0.830810000 | 0.270200000 |
| 0.247790000 | 0.824310000 | 0.326880000 |
| 0.155360000 | 0.825920000 | 0.349520000 |
| 0.057090000 | 0.832380000 | 0.290530000 |
| 0.075480000 | 0.863540000 | 0.167100000 |
| 0.155210000 | 0.833180000 | 0.227360000 |
| 0.156630000 | 0.831820000 | 0.147670000 |
| 0.074980000 | 0.864730000 | 0.207070000 |
| 0.410910000 | 0.580280000 | 0.086820000 |
| 0.405210000 | 0.578760000 | 0.374130000 |
| 0.234960000 | 0.803770000 | 0.170490000 |
| 0.807370000 | 0.578380000 | 0.148200000 |
| 0.907780000 | 0.582270000 | 0.208280000 |
| 0.499870000 | 0.584910000 | 0.152060000 |
| 0.905870000 | 0.580790000 | 0.127800000 |
| 0.808500000 | 0.580160000 | 0.188210000 |
| 0.831320000 | 0.602640000 | 0.312800000 |
| 0.910360000 | 0.580940000 | 0.251200000 |
| 0.916140000 | 0.574290000 | 0.331040000 |
| 0.827830000 | 0.606180000 | 0.272750000 |
| 0.657620000 | 0.830810000 | 0.391590000 |
| 0.660220000 | 0.830570000 | 0.105480000 |
| 0.995110000 | 0.551220000 | 0.307190000 |
| 0.556760000 | 0.831200000 | 0.329720000 |
| 0.652500000 | 0.828390000 | 0.269720000 |
| 0.745620000 | 0.823820000 | 0.326610000 |
| 0.654010000 | 0.828850000 | 0.348910000 |
| 0.555270000 | 0.830670000 | 0.289670000 |
| 0.574710000 | 0.862450000 | 0.166830000 |
| 0.654290000 | 0.831380000 | 0.226960000 |
| 0.656100000 | 0.831100000 | 0.147440000 |
| 0.574090000 | 0.863170000 | 0.206770000 |
| 0.908820000 | 0.578730000 | 0.086060000 |
| 0.920000000 | 0.572210000 | 0.374280000 |
| 0.734660000 | 0.803150000 | 0.170150000 |
| 0.670840000 | 0.608600000 | 0.518390000 |
| 0.159090000 | 0.592400000 | 0.525080000 |
| 0.702970000 | 0.600990000 | 0.561080000 |
| 0.878800000 | 0.573720000 | 0.598710000 |
| 0.807290000 | 0.565270000 | 0.637690000 |
| 0.709110000 | 0.562010000 | 0.699220000 |
| 0.690630000 | 0.488430000 | 0.732740000 |
| 0.729270000 | 0.606190000 | 0.633160000 |
| 0.625570000 | 0.643810000 | 0.624650000 |
| 0.711230000 | 0.445750000 | 0.574640000 |
| 0.163680000 | 0.648680000 | 0.562860000 |
| 0.077910000 | 0.546360000 | 0.623770000 |
| 0.098440000 | 0.520940000 | 0.666290000 |
| 0.184580000 | 0.581000000 | 0.696010000 |
| 0.155500000 | 0.539840000 | 0.737260000 |
| 0.216320000 | 0.689990000 | 0.631370000 |
| 0.318180000 | 0.689360000 | 0.655170000 |
| 0.298270000 | 0.495590000 | 0.658220000 |
| 0.310180000 | 0.429260000 | 0.691940000 |
| 0.818620000 | 0.461020000 | 0.552400000 |
| 0.239030000 | 0.409330000 | 0.016100000 |
| 0.409700000 | 0.329400000 | 0.030900000 |
| 0.082130000 | 0.250590000 | 0.017080000 |
| 0.224620000 | 0.291750000 | 0.087250000 |
| 0.254070000 | 0.341860000 | 0.408420000 |
| 0.063860000 | 0.301130000 | 0.411650000 |
| 0.481410000 | 0.400970000 | 0.451530000 |
| 0.163190000 | 0.340530000 | 0.480150000 |
| 0.345930000 | 0.241750000 | 0.465880000 |
| 0.095760000 | 0.372330000 | 0.085790000 |
| 0.174770000 | 0.052160000 | 0.488530000 |
| 0.110980000 | 0.117110000 | 0.500040000 |
| 0.464070000 | 0.142940000 | 0.460970000 |
| 0.164460000 | 0.119660000 | 0.421120000 |
| 0.356750000 | 0.501810000 | 0.457990000 |
| 0.466630000 | 0.022970000 | 0.390440000 |
| 0.005160000 | 0.089040000 | 0.069800000 |
| 0.319440000 | 0.074520000 | 0.066530000 |
| 0.222730000 | 0.164150000 | 0.009070000 |
| 0.411210000 | 0.078800000 | 0.991510000 |
| 0.098910000 | 0.496680000 | 0.008060000 |
| 0.349390000 | 0.115050000 | 0.390940000 |
| 0.738260000 | 0.408850000 | 0.012880000 |
| 0.910760000 | 0.331410000 | 0.030750000 |
| 0.582000000 | 0.249960000 | 0.014120000 |
| 0.723630000 | 0.290360000 | 0.084130000 |
| 0.761520000 | 0.346780000 | 0.405730000 |
| 0.570770000 | 0.288600000 | 0.405170000 |
| 0.975140000 | 0.405680000 | 0.468190000 |
| 0.832540000 | 0.363120000 | 0.552370000 |
| 0.826810000 | 0.249550000 | 0.470340000 |
| 0.596650000 | 0.372940000 | 0.082880000 |
| 0.712850000 | 0.090140000 | 0.503540000 |
| 0.592180000 | 0.043540000 | 0.500970000 |
| 0.951700000 | 0.141260000 | 0.462780000 |
| 0.658700000 | 0.115340000 | 0.427840000 |
| 0.841770000 | 0.497460000 | 0.459400000 |
| 0.963000000 | 0.029660000 | 0.394930000 |
| 0.504940000 | 0.089780000 | 0.067740000 |
| 0.819940000 | 0.074560000 | 0.068290000 |
| 0.724300000 | 0.163710000 | 0.008290000 |
| 0.912030000 | 0.079780000 | 0.992890000 |
| 0.597340000 | 0.496790000 | 0.007270000 |
| 0.837480000 | 0.113820000 | 0.393890000 |
| 0.642270000 | 0.325360000 | 0.484320000 |
| 0.240050000 | 0.909150000 | 0.016980000 |
| 0.411390000 | 0.830660000 | 0.034250000 |
| 0.082640000 | 0.751020000 | 0.017530000 |
| 0.224590000 | 0.789140000 | 0.087910000 |
| 0.241740000 | 0.811730000 | 0.409680000 |
| 0.074300000 | 0.838410000 | 0.409770000 |
| 0.462010000 | 0.911200000 | 0.459850000 |
| 0.174420000 | 0.786900000 | 0.502470000 |
| 0.347150000 | 0.738280000 | 0.462130000 |
| 0.098790000 | 0.873030000 | 0.086730000 |
| 0.250770000 | 0.588600000 | 0.509650000 |
| 0.064440000 | 0.564630000 | 0.507990000 |
| 0.515350000 | 0.668830000 | 0.450870000 |
| 0.158900000 | 0.560670000 | 0.436350000 |
| 0.288430000 | 0.978930000 | 0.450000000 |
| 0.474100000 | 0.538380000 | 0.388600000 |
| 0.002660000 | 0.586440000 | 0.067750000 |
| 0.319130000 | 0.572240000 | 0.067180000 |
| 0.224420000 | 0.665300000 | 0.009710000 |
| 0.411970000 | 0.581790000 | 0.992150000 |
| 0.097690000 | 0.995450000 | 0.009310000 |
| 0.342850000 | 0.616120000 | 0.393570000 |
| 0.273420000 | 0.857050000 | 0.517020000 |
| 0.079430000 | 0.827620000 | 0.506040000 |
| 0.741000000 | 0.909540000 | 0.017590000 |
| 0.911140000 | 0.829690000 | 0.034300000 |
| 0.582650000 | 0.751250000 | 0.017440000 |
| 0.723960000 | 0.788440000 | 0.087790000 |
| 0.743660000 | 0.824870000 | 0.409200000 |
| 0.569400000 | 0.838500000 | 0.408630000 |
| 0.965500000 | 0.923280000 | 0.462890000 |
| 0.683280000 | 0.783860000 | 0.502960000 |
| 0.846340000 | 0.747800000 | 0.456420000 |
| 0.598930000 | 0.873160000 | 0.086600000 |
| 0.757490000 | 0.672860000 | 0.492060000 |
| 0.558140000 | 0.549550000 | 0.501880000 |
| 0.996450000 | 0.665820000 | 0.454040000 |
| 0.666900000 | 0.565300000 | 0.429070000 |
| 0.766940000 | 0.977310000 | 0.454290000 |
| 0.982050000 | 0.530470000 | 0.391570000 |
| 0.504610000 | 0.587230000 | 0.068560000 |
| 0.816540000 | 0.569010000 | 0.066510000 |
| 0.723960000 | 0.665020000 | 0.009470000 |
| 0.912150000 | 0.582020000 | 0.991560000 |
| 0.598960000 | 0.995480000 | 0.009060000 |
| 0.857090000 | 0.615090000 | 0.391130000 |
| 0.779140000 | 0.853020000 | 0.518710000 |
| 0.585170000 | 0.823490000 | 0.505410000 |
| 0.990510000 | 0.267720000 | 0.530210000 |
| 0.247580000 | 0.358030000 | 0.510630000 |
| 0.648710000 | 0.750100000 | 0.615160000 |
| 0.501400000 | 0.579470000 | 0.628640000 |
| 0.636850000 | 0.380950000 | 0.725480000 |
| 0.741100000 | 0.522900000 | 0.771860000 |
| 0.048350000 | 0.543600000 | 0.756500000 |
| 0.236380000 | 0.500910000 | 0.759380000 |
| 0.436560000 | 0.740480000 | 0.636730000 |
| 0.320010000 | 0.758940000 | 0.687900000 |
| 0.379110000 | 0.363320000 | 0.681610000 |
| 0.193870000 | 0.371150000 | 0.705380000 |
| 0.839990000 | 0.552110000 | 0.526820000 |
| 0.063540000 | 0.368800000 | 0.030210000 |
| 0.257150000 | 0.292160000 | 0.031270000 |
| 0.311470000 | 0.359920000 | 0.461750000 |
| 0.997680000 | 0.277800000 | 0.483110000 |
| 0.287670000 | 0.111110000 | 0.447030000 |
| 0.044560000 | 0.061230000 | 0.453370000 |
| 0.050220000 | 0.110550000 | 0.016310000 |
| 0.272160000 | 0.048940000 | 0.014240000 |
| 0.562380000 | 0.367560000 | 0.027790000 |
| 0.758120000 | 0.292820000 | 0.028750000 |
| 0.816190000 | 0.374100000 | 0.492790000 |
| 0.518150000 | 0.282510000 | 0.456590000 |
| 0.793320000 | 0.115250000 | 0.453000000 |
| 0.530410000 | 0.049940000 | 0.448970000 |
| 0.550100000 | 0.110380000 | 0.014550000 |
| 0.773120000 | 0.049210000 | 0.015630000 |
| 0.063850000 | 0.868170000 | 0.031660000 |
| 0.258900000 | 0.792530000 | 0.032290000 |
| 0.307460000 | 0.866810000 | 0.462960000 |
| 0.001050000 | 0.791090000 | 0.459080000 |
| 0.297940000 | 0.601660000 | 0.453320000 |
| 0.011210000 | 0.529260000 | 0.452880000 |
| 0.050240000 | 0.611450000 | 0.015060000 |
| 0.272600000 | 0.549720000 | 0.014670000 |
| 0.563930000 | 0.868390000 | 0.031520000 |
| 0.758840000 | 0.792160000 | 0.032230000 |
| 0.808850000 | 0.874780000 | 0.464090000 |
| 0.504720000 | 0.787690000 | 0.459320000 |
| 0.807910000 | 0.608870000 | 0.447910000 |
| 0.522090000 | 0.537860000 | 0.444510000 |
| 0.550320000 | 0.611550000 | 0.015500000 |
| 0.771980000 | 0.549110000 | 0.013640000 |
| 0.998100000 | 0.082110000 | 0.192010000 |
| 0.485090000 | 0.051640000 | 0.268760000 |
| 0.246140000 | 0.332780000 | 0.288130000 |
| 0.231170000 | 0.304180000 | 0.208440000 |
| 0.499810000 | 0.083270000 | 0.189380000 |
| 0.982740000 | 0.053070000 | 0.272310000 |
| 0.755300000 | 0.336940000 | 0.283600000 |
| 0.735870000 | 0.305940000 | 0.204640000 |
| 0.002870000 | 0.584690000 | 0.188880000 |
| 0.484820000 | 0.560510000 | 0.270170000 |
| 0.248370000 | 0.826640000 | 0.288350000 |
| 0.234960000 | 0.804340000 | 0.208900000 |
| 0.498610000 | 0.585010000 | 0.190460000 |
| 0.993490000 | 0.554620000 | 0.268550000 |
| 0.746130000 | 0.823600000 | 0.288070000 |
| 0.734480000 | 0.803150000 | 0.208540000 |
| 0.787960000 | 0.543880000 | 0.565580000 |
| 0.691820000 | 0.514480000 | 0.659460000 |
| 0.178830000 | 0.611600000 | 0.602230000 |
| 0.295310000 | 0.589850000 | 0.676350000 |
| 0.233020000 | 0.078600000 | 0.130810000 |
| 0.078960000 | 0.086520000 | 0.139640000 |
| 0.229090000 | 0.079240000 | 0.202530000 |
| 0.259380000 | 0.132930000 | 0.257290000 |
| 0.261070000 | 0.124090000 | 0.329340000 |
| 0.048110000 | 0.029800000 | 0.325230000 |
| 0.486870000 | 0.313910000 | 0.344680000 |
| 0.326100000 | 0.337550000 | 0.340960000 |
| 0.479950000 | 0.322130000 | 0.272690000 |
| 0.006260000 | 0.385320000 | 0.221780000 |
| 0.008600000 | 0.383620000 | 0.149960000 |
| 0.298080000 | 0.281860000 | 0.155990000 |
| 0.732480000 | 0.077270000 | 0.132920000 |
| 0.579530000 | 0.087440000 | 0.136800000 |
| 0.726980000 | 0.077360000 | 0.204350000 |
| 0.753420000 | 0.128100000 | 0.259310000 |
| 0.753120000 | 0.120740000 | 0.331370000 |
| 0.550630000 | 0.025230000 | 0.321140000 |
| 0.976510000 | 0.310180000 | 0.348200000 |
| 0.837130000 | 0.341260000 | 0.336120000 |
| 0.971550000 | 0.317600000 | 0.275490000 |
| 0.512170000 | 0.388450000 | 0.218920000 |
| 0.510880000 | 0.384410000 | 0.146940000 |
| 0.799880000 | 0.281910000 | 0.151820000 |
| 0.231400000 | 0.574660000 | 0.131080000 |
| 0.079900000 | 0.588720000 | 0.135810000 |
| 0.226000000 | 0.576780000 | 0.202680000 |
| 0.254130000 | 0.633530000 | 0.256480000 |
| 0.253990000 | 0.629490000 | 0.328530000 |
| 0.062650000 | 0.528850000 | 0.320330000 |
| 0.484610000 | 0.833600000 | 0.346750000 |
| 0.324450000 | 0.821820000 | 0.341260000 |
| 0.479140000 | 0.830980000 | 0.274470000 |
| 0.014930000 | 0.892090000 | 0.222390000 |
| 0.014600000 | 0.887290000 | 0.150620000 |
| 0.300410000 | 0.780280000 | 0.156420000 |
| 0.731510000 | 0.574720000 | 0.132040000 |
| 0.579190000 | 0.587770000 | 0.138130000 |
| 0.731860000 | 0.578090000 | 0.203660000 |
| 0.763360000 | 0.630620000 | 0.258770000 |
| 0.769680000 | 0.622080000 | 0.330570000 |
| 0.549980000 | 0.538940000 | 0.323320000 |
| 0.986140000 | 0.832030000 | 0.347760000 |
| 0.821350000 | 0.820290000 | 0.341360000 |
| 0.981030000 | 0.833950000 | 0.275610000 |
| 0.514060000 | 0.890380000 | 0.222170000 |
| 0.513810000 | 0.886300000 | 0.150390000 |
| 0.800460000 | 0.780310000 | 0.155970000 |
| 0.625160000 | 0.565020000 | 0.579190000 |
| 0.744790000 | 0.678420000 | 0.571660000 |
| 0.931520000 | 0.522060000 | 0.600580000 |
| 0.935770000 | 0.651670000 | 0.594350000 |
| 0.855200000 | 0.623390000 | 0.659160000 |
| 0.801610000 | 0.607160000 | 0.700800000 |
| 0.680200000 | 0.547160000 | 0.789290000 |
| 0.634440000 | 0.414470000 | 0.555370000 |
| 0.712150000 | 0.415630000 | 0.605050000 |
| 0.225710000 | 0.509670000 | 0.790930000 |
| 0.114300000 | 0.597550000 | 0.679820000 |
| 0.014900000 | 0.588760000 | 0.625230000 |
| 0.038190000 | 0.476760000 | 0.606640000 |
| 0.084990000 | 0.668920000 | 0.562840000 |
| 0.234610000 | 0.715230000 | 0.558730000 |
| 0.142080000 | 0.690950000 | 0.650340000 |
| 0.247850000 | 0.755320000 | 0.613390000 |
| 0.139430000 | 0.460870000 | 0.665770000 |
| 0.379000000 | 0.515270000 | 0.640500000 |
| 0.223880000 | 0.458850000 | 0.638630000 |
| 0.357030000 | 0.476530000 | 0.716790000 |
| 0.200480000 | 0.326450000 | 0.730850000 |
| 0.860510000 | 0.523140000 | 0.645790000 |
| 0.656930000 | 0.614630000 | 0.703970000 |
| 0.815470000 | 0.671500000 | 0.641660000 |
| 0.758300000 | 0.574220000 | 0.605180000 |
| 0.899160000 | 0.493940000 | 0.565990000 |
| 0.840440000 | 0.629700000 | 0.536610000 |
| 0.519220000 | 0.469320000 | 0.656030000 |
| 0.453190000 | 0.644120000 | 0.671200000 |

**References：**

[1] X. He, X. Gao, X. Chen, S. Hu, F. Tan, Y. Xiong, R. Long, M. Liu, E. C. M. Tse, F. Wei, H. Yang, J. Hou, C. Song, X. Guo, *Appl. Catal., B* **2023**, *327*, 122418, <https://doi.org/https://doi.org/10.1016/j.apcatb.2023.122418>.

[2] S. He, J. Yang, S. Liu, X. Wang, J. Qiu, *Adv. Funct. Mater.* **2024**, *34* (17), 2314133, <https://doi.org/https://doi.org/10.1002/adfm.202314133>.

[3] a) G. Kresse, J. Furthmüller, *Sci* **1996**, *6* (1), 15; b) G. Kresse, D. Joubert, *Physical Review B* **1999**, *59* (3), 1758, <https://doi.org/10.1103/PhysRevB.59.1758>.

[4] J. P. Perdew, K. Burke, M. Ernzerhof, *Phys. Rev. Lett.* **1996**, *77* (18), 3865, <https://doi.org/10.1103/PhysRevLett.77.3865>.

[5] M. Methfessel, A. T. Paxton, *Physical Review B* **1989**, *40* (6), 3616, <https://doi.org/10.1103/PhysRevB.40.3616>.

[6] J. Wang, H. Zhang, N. Mu, T. Bo, J. Zhang, X. Tan, W. Zhou, T. Yu, *Applied Catalysis B: Environment and Energy* **2025**, *377*, 125480, <https://doi.org/https://doi.org/10.1016/j.apcatb.2025.125480>.

[7] Y. Nie, Y. Li, C. An, X. Tan, Z. Hu, J. Ye, T. Yu, *Applied Catalysis B: Environment and Energy* **2024**, *345*, 123704, <https://doi.org/https://doi.org/10.1016/j.apcatb.2024.123704>.

[8] K. Das, S. Chakraborty, S. Kediya, A. K. Singh, R. Das, S. Mondal, M. Riyaz, D. Goud, N. Dutta, C. P. Vinod, S. C. Peter, *Angew. Chem. Int. Ed.* **2025**, *64* (28), e202423471, <https://doi.org/https://doi.org/10.1002/anie.202423471>.

[9] S. Chakraborty, R. Das, M. Riyaz, K. Das, A. K. Singh, D. Bagchi, C. P. Vinod, S. C. Peter, *Angew. Chem. Int. Ed.* **2023**, *62* (9), e202216613, <https://doi.org/https://doi.org/10.1002/anie.202216613>.

[10] J. Wang, C. Yang, L. Mao, X. Cai, Z. Geng, H. Zhang, J. Zhang, X. Tan, J. Ye, T. Yu, *Adv. Funct. Mater.* **2023**, *33* (28), 2213901, <https://doi.org/https://doi.org/10.1002/adfm.202213901>.

[11] W. Gao, L. Shi, W. Hou, C. Ding, Q. Liu, R. Long, H. Chi, Y. Zhang, X. Xu, X. Ma, Z. Tang, Y. Yang, X. Wang, Q. Shen, Y. Xiong, J. Wang, Z. Zou, Y. Zhou, *Angew. Chem. Int. Ed.* **2024**, *63* (9), e202317852, <https://doi.org/https://doi.org/10.1002/anie.202317852>.

[12] W. Xie, K. Li, X.-H. Liu, X. Zhang, H. Huang, *Adv. Mater.* **2023**, *35* (3), 2208132, <https://doi.org/https://doi.org/10.1002/adma.202208132>.

[13] Y. Mao, M. Zhang, G. Zhai, S. Si, D. Liu, K. Song, Y. Liu, Z. Wang, Z. Zheng, P. Wang, Y. Dai, H. Cheng, B. Huang, *Adv. Sci.* **2024**, *11* (28), 2401933, <https://doi.org/https://doi.org/10.1002/advs.202401933>.

[14] R. Xu, D.-H. Si, S.-S. Zhao, Q.-J. Wu, X.-S. Wang, T.-F. Liu, H. Zhao, R. Cao, Y.-B. Huang, *Journal of the American Chemical Society* **2023**, *145* (14), 8261, <https://doi.org/10.1021/jacs.3c02370>.

[15] B. Zhao, X. Qiu, Y. Song, S. Li, K. Zhang, Z. Mou, Q. Wang, B. Zhang, Z. Wang, *Small* **2025**, *21* (15), 2500877, <https://doi.org/https://doi.org/10.1002/smll.202500877>.

[16] J. Zhao, H. Chen, B. Wang, M. Ji, D. Su, L. Song, P. Zhang, Y. She, Y.-X. Weng, H. Li, J. Xia, *Applied Catalysis B: Environment and Energy* **2025**, *361*, 124647, <https://doi.org/https://doi.org/10.1016/j.apcatb.2024.124647>.

[17] D. Wen, N. Wang, J. Peng, T. Majima, J. Jiang, *Chin. J. Catal.* **2025**, *69*, 58, <https://doi.org/https://doi.org/10.1016/S1872-2067(24)60183-X>.
